# Longitudinal Changes in Circulating Metabolites and Lipoproteins After Breast Cancer Treatment

**DOI:** 10.3389/fonc.2022.919522

**Published:** 2022-06-16

**Authors:** Guro F. Giskeødegård, Torfinn S. Madssen, Matteo Sangermani, Steinar Lundgren, Torgeir Wethal, Trygve Andreassen, Randi J. Reidunsdatter, Tone F. Bathen

**Affiliations:** ^1^ K.G. Jebsen Center for Genetic Epidemiology, Department of Public Health and Nursing, Norwegian University of Science and Technology (NTNU), Trondheim, Norway; ^2^ Clinic of Surgery, St. Olavs University Hospital, Trondheim, Norway; ^3^ Department of Circulation and Medical Imaging, Norwegian University of Science and Technology (NTNU), Trondheim, Norway; ^4^ Department of Clinical and Molecular Medicine, Norwegian University of Science and Technology (NTNU), Trondheim, Norway; ^5^ Department of Neuromedicine and Movement Science, Norwegian University of Science and Technology (NTNU), Trondheim, Norway; ^6^ Department of Medicine, Stroke Unit, St. Olavs University Hospital, Trondheim, Norway

**Keywords:** radiation, hormone receptor, lipoprotein subfractions, NMR metabolic profiling, breast cancer survival, chemotherapy

## Abstract

The multimodal treatment of breast cancer may induce long term effects on the metabolic profile and increase the risk of future cardiovascular disease. In this study, we characterized longitudinal changes in serum lipoprotein subfractions and metabolites after breast cancer treatment, aiming to determine the long-term effect of different treatment modalities. Further, we investigated the prognostic value of treatment-induced changes in breast cancer-specific and overall 10-year survival. In this study, serum samples from breast cancer patients (n = 250) were collected repeatedly before and after radiotherapy, and serum metabolites and lipoprotein subfractions were quantified by NMR spectroscopy. Longitudinal changes were assessed by univariate and multivariate data analysis methods applicable for repeated measures. Distinct changes were detectable in levels of lipoprotein subfractions and circulating metabolites during the first year, with similar changes despite large differences in treatment regimens. We detect increased free cholesterol and decreased esterified cholesterol levels of HDL subfractions, a switch towards larger LDL particles and higher total LDL-cholesterol, in addition to a switch in the glutamine-glutamate ratio. Non-survivors had different lipid profiles from survivors already at baseline. To conclude, our results show development towards an atherogenic lipid profile in breast cancer patients with different treatment regimens.

## Introduction

Breast cancer is the most common cancer among women, with increasing incidence rates (1). The increased incidence rates together with decreased mortality have resulted in an increasing number of breast cancer survivors, several suffering long-term symptoms after treatment (2–5). Treatment for breast cancer is multimodal, and depending on tumor characteristics and stage, includes surgical removal of the tumor followed by chemotherapy, radiotherapy (RT), endocrine treatment, and targeted treatment for HER-2 (6). Patients with locally advanced tumors will receive chemotherapy prior to surgery to downstage the tumor and make large tumors operable.

The multimodal treatment regimen for breast cancer may result in long-term effects for patients, which may affect both the health and lifespan of breast cancer survivors. Both anthracycline-based chemotherapy and trastuzumab are well-known to have cardiotoxic effects, which may lead to heart failure (4). Similarly, RT may result in cardiac damage, particularly for left-sided breast tumors due to the proximity to the heart (5), however this effect is less pronounced with current RT regimens (7, 8). Endocrine treatment, such as tamoxifen, aromatase inhibitors, and ovarian suppression therapy may cause vasomotor symptoms, genitourinary dryness, and sleep disturbances (9, 10). Changes in cardiovascular risk factors have also been described following adjuvant treatment, and cardiovascular diseases (CVDs) are a competing cause of death in breast cancer patient (11). Several studies have demonstrated a greater risk of death from CVDs in breast cancer survivors compared to women without breast cancer (12–14). There is a need to further our understanding of the systemic effects of breast cancer treatment and its impact on patient outcome.

The measurement of circulating lipoproteins and metabolites may provide valuable insight into the systemic effects of treatment for breast cancer. Lipoproteins are divided into the subclasses very-low density (VLDL), intermediate-density (IDL), low-density (LDL), and high-density lipoproteins (HDL). Lipoproteins are lipid carriers transporting triglycerides, phospholipids, and cholesterol to cells throughout the body, and levels of the different lipoproteins are frequently reported to reflect cardiovascular risk (15). Cholesterol levels in LDL (LDL-C) are shown to be an important cause of atherosclerosis (16, 17). Previous studies have shown increased levels of total cholesterol and LDL-C after chemotherapy for breast cancer (18). Moreover, a favorable effect of tamoxifen has been reported on the lipoprotein profile of breast cancer patients, with reduction in LDL-C, still clinical trials have failed to demonstrate a protective role of tamoxifen on cardiovascular end points (19). Conventional methods for lipoprotein quantification can however only measure total values of LDL and HDL- cholesterol and total triglycerides, not reflecting the delicate density range of the lipoprotein subclasses and the lipids they carry.

Nuclear magnetic resonance (NMR) spectroscopy allows the quantification of a wide range of lipoprotein subfraction parameters, providing a much more detailed picture than traditional lipoprotein measurements. Simultaneously NMR allows accurate quantification of circulating small-molecular metabolites, providing information on the active metabolic processes. Recent studies suggest that smaller LDLs and triglyceride-rich lipoproteins are additional risk factors for CVD (20, 21), demonstrating the importance of more detailed characterization. We have previously described significant changes in lipoprotein parameters after treatment into an unfavorable lipid profile, as well as increased levels of metabolites associated with inflammation and oxidative stress after treatment in a small cohort of breast cancer patients (22).

In the present study, we have characterized longitudinal changes in serum lipoprotein subfractions and metabolites by repeated serum collection for one year after breast cancer RT. The aim of our study was to determine the longitudinal effect of different treatment modalities on circulating lipoprotein subfractions and metabolites, hypothesizing that different treatment modalities would have different effects on serum profiles. Furthermore, we investigated the prognostic value of treatment-induced changes in circulating markers on both breast cancer-specific and overall 10-year survival.

## Materials and Methods

### Study Population and Study Design

The study population included 250 breast cancer patients participating in a prospective longitudinal study investigating side effect and health-related quality of life after RT. Patients referred for post-operative local or locoregional RT at St. Olavs University Hospital, Trondheim, Norway, were included consecutively between 2007-2008. All patients had undergone surgical removal of the tumor prior to RT. Exclusion criteria were metastatic disease, physical or psychological disorders that would interfere with participation, and the inability to read or understand Norwegian language. More information on the study recruitment, follow-up, and compliance have been reported previously (23). The study was approved by the Regional Committee for Medical Research Ethics (REK 2019/13760), and all patients gave informed written consent to participate in the study.

### Treatment Regimen

Patients received standard treatment according to Norwegian guidelines at the time of inclusion (Norwegian Breast Cancer Group, nbcg.no). All patients in this cohort received RT, either local or locoregional, after surgical removal of the tumor. Patients who received chemotherapy had completed this treatment before starting RT. Chemotherapy was administered as six anthracycline-based courses (fluorouracil, epirubicin, and cyclophosphamide), or four anthracycline-based courses followed by four courses of taxanes (docetaxel). Patients with hormone receptor positive tumors (>10% staining) received endocrine treatment by either tamoxifen or aromatase inhibitors depending on menopausal status for a duration of five years, with treatment starting concurrent with or immediately after RT. Patients with HER-2 overexpressing tumors received trastuzumab (Herceptin) for one year, starting at the same time as receiving docetaxel treatment.

For local RT, 50 Gy was delivered to the breast/chest wall in 2 Gy/fraction, five days a week for five weeks. For locoregional RT, additional 46 Gy was delivered to lymph nodes in the periclavicular region with or without the axillae. Patients below 40 years of age with breast-conserving surgery received an additional boost dose of up to 66 Gy to the tumor bed.

**Figure 1:**
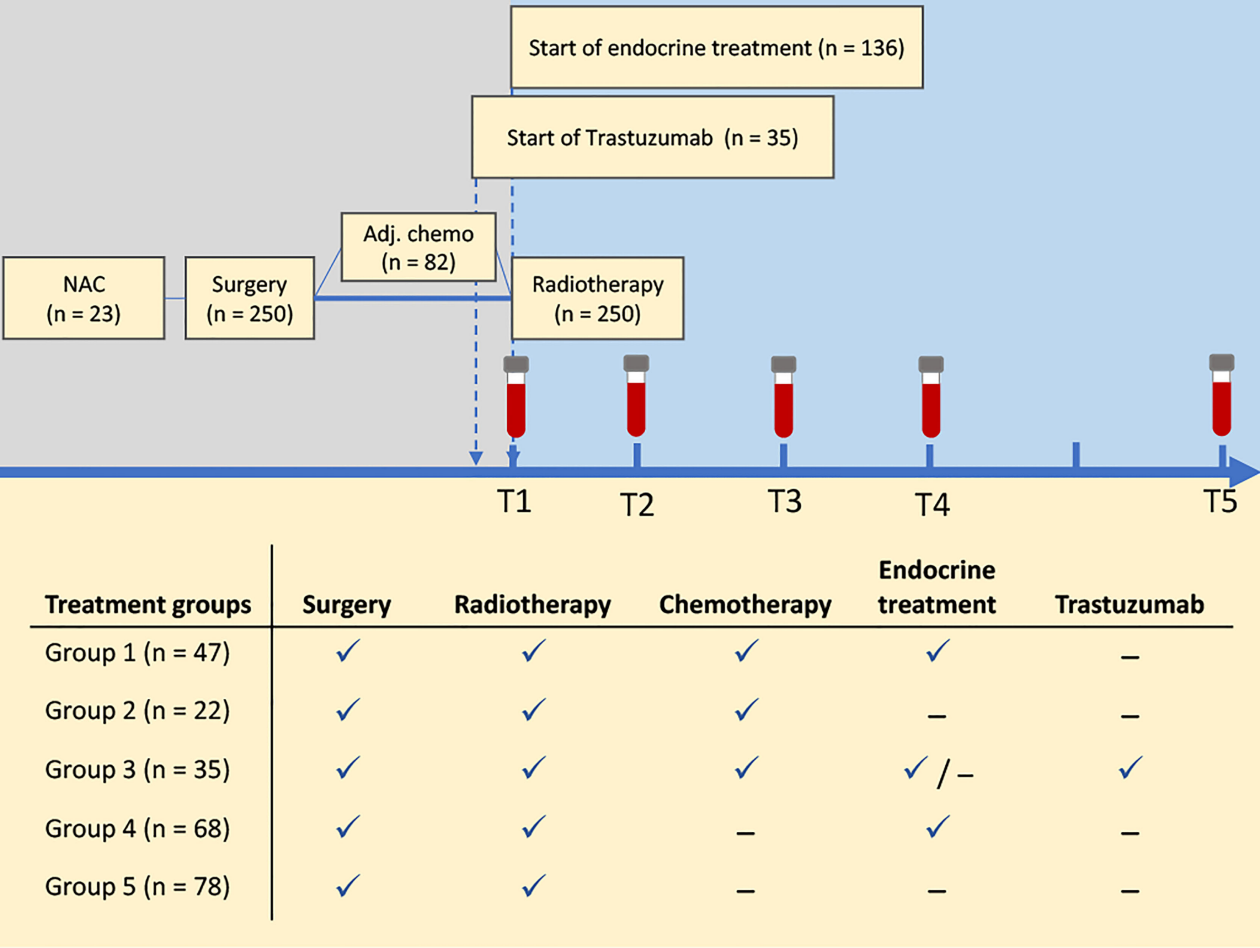
Study overview. Patients were treated according to national guidelines at the time of inclusion. Serum samples were collected at five time points before and after radiation treatment, and follow-up data on recurrence and survival were collected after 10 years. Patients were categorized into five treatment groups for assessment of treatment-related changes in serum metabolite and lipoprotein profiles. NAC, neoadjuvant chemotherapy.

Serum samples were collected in accordance with clinical study controls at the hospital at five time points: before RT (T1, n = 229), immediately after end of RT (T2, n = 211), and after 3 months (T3, n = 198), 6 months (T4, n = 195), and 12 months (T5, n = 146). Additionally, follow-up data on recurrence, overall- and breast cancer specific survival were collected 10 years after inclusion to the study. The treatment regimens and collection of serum samples are summarized in **Figure 1**.

### Lipoprotein and Metabolite Quantification

Serum samples were analysed by NMR spectroscopy for quantification of lipoproteins and metabolites. Samples were thawed at room temperature prior to analysis, before mixing 150 µL serum with 150 µL buffer (20% D_2_O with 0.075 M Na_2_HPO_4_, 6.2 mM NaN, 4.6 mM TSP, pH 7.4). Samples were analysed in 3 mm NMR tubes with a Bruker Avance III 600 MHz spectrometer (Bruker Biospin Gmbh, Germany) equipped with a 5 mm QCI cryoprobe. Experiments were fully automated using a SampleJet with IconNMR on TopSpin 3.1 software. Carr-Purcelli-Meiboom-Gill (CPMG) and nuclear Overhauser effect spectroscopy (NOESY) spectra were acquired with water-suppression at a temperature of 37°C. The spectra were Fourier transformed into 128k real data points after 0.3 Hz exponential line broadening. Quality control (QC) samples were prepared from pooled serum samples from anonymous donors and analyzed daily (approximately one QC sample for every 60^th^ sample).

Lipoprotein parameters were automatically quantified using the commercial Bruker Lipoprotein Subclass Analysis (B.I.LISA™) method from Bruker BioSpin (24, 25). This method provides concentrations of esterified and free cholesterol, triglycerides, and phospholipids in serum (total values), and within each of the main lipoprotein classes: VLDL, IDL, LDL, and HDL. Additionally, concentrations are reported for subclasses based on density range: VLDL 1-5, LDL 1-6, and HDL 1-4. Further, concentrations of apolipoproteins (Apo-A1, Apo-A2, and Apo-B) in serum, LDL, HDL, and in the subclasses HDL 1-4 and LDL 1-6 are provided, amounting to a total of 100 variables.

Metabolites were assigned using Chenomx NMR suite 7.7 (Chenomx Inc., Alberta, Canada), the Human Metabolome Database and previous metabolite identifications by HSQC (26). Metabolite quantification was performed from CPMG spectra in Matlab R2019b. The spectra were first referenced to the left peak of the alanine doublet at 1.47 ppm, and peak aligned using the icoshift algorithm (27) with the spectrum with the highest correlation to the remaining spectra as reference. Metabolite peaks were integrated, and peaks were corrected for the number of protons giving rise to the integrated signal. Peak integrals were then adjusted for T2 relaxation times acquired from three serum samples from anonymous donors, as described previously (28). For metabolites with more than one resonance, either the mean of resonances or the resonance in a non-overlapping spectral region was chosen. The resulting concentration of glucose was set equal to the absolute concentration of glucose available from automatic metabolite quantification by Bruker BioSpin B.I.Quant-PS™ routines. The remaining metabolite concentrations were scaled accordingly using the same scaling factor as for glucose to achieve absolute quantification of all metabolites. Quantification resulted in concentrations of 26 distinct peaks (24 metabolites and two lipid peaks), where the lipid signals arise from the methyl (−CH3) groups at 0.85 ppm (lipid1) and methylene (−CH2−) groups at 1.57 ppm (lipid2), mainly from triglycerides and esterified cholesterol within the lipoprotein particles.

### Statistical Analysis

Differences in clinical variables between survivors and non-survivors were assessed by t-tests for continuous variables and chi-square tests for categorical variables. Tumor size was log transformed before analysis to achieve normally distributed data (assessed by qq-plots). Longitudinal changes in metabolite and lipoprotein profiles were assessed by univariate and multivariate models. Univariate linear mixed effect models (LMMs) were used to assess changes from baseline measurements (T1) to the measurements at each of the timepoints T2-T5. The mixed models included the main effect for timepoints, treatment group/survival group, and the time-group interactions as fixed effects, and a random intercept was included for each patient. The time variable was reference coded to the baseline measurement (T1). For assessment of longitudinal changes with different treatments regimens, patients were divided into five treatment groups (**Figure 1**). Treatment groups were sum coded in statistical analysis to detect possible groups deviating from the average response. For comparison of local and loco-regional RT, treatment was reference coded with local RT as the reference group, while survival analysis was reference coded with survivors as the reference group. Multiple testing correction was performed with the Benjamini-Hochberg procedure adjusting for the number of variables tested, and adjusted p-values < 0.05 were considered significant.

Repeated measures ANOVA simultaneous component analysis+ (RM-ASCA+) was used for multivariate analysis (29). This method extends repeated measures linear mixed models to the multivariate case, by first decomposing the multivariate response matrix into effect matrices according to the specified LMM. The resulting effect matrices are then analyzed by principal component analysis (PCA), and the results are summarized into PCA scores and loadings. The above-mentioned LMMs were used in the RM-ASCA+ analysis. RM-ASCA+ allows the effect matrices from LMMs to be analysed either separately or combined. For instance, the effect matrix of ‘time*group interaction’ can be analysed separately to highlight deviations in time development of a group compared to the reference group. Alternatively, the effect matrix combining ‘group + time*group interaction’ can be analysed to display possible baseline differences between the groups together with their development over time compared to the reference group. An effect matrix combining ‘time + group + time*group interaction’ will show the time development of all groups, including the reference group and display possible baseline differences between them in one plot. Non-parametric bootstrapping was used to construct 95% confidence intervals for the scores and loadings. Bootstrapping was performed by resampling until the original sample size was achieved, and the process was repeated 1000 times. The 2.5^th^ and 97.5^th^ percentiles of the bootstrapped estimates were used as the lower and upper bounds for the intervals. All data analyses were performed in Matlab 2020b. RM-ASCA+ scripts are available from github (https://github.com/ntnu-mr-cancer/RM_ASCA).

## Results

### Patient Characteristics

Clinical variables of the patient cohort are shown in **Table 1**. Patients has a mean age of 58.1 years, and 76.4% of the patients were diagnosed with invasive ductal carcinoma. Non-survivors (overall survival) had higher BMI (mean BMI: 27.8 vs 26.2, p = 0.031) and more advanced cancer disease with significantly larger tumors (median tumor size: 1.75 vs 1.50 cm, p = 0.004), higher tumor stage (p<0.001), and more lymph node involvement (lymphatic involvement: 45.0% vs 26.8%, p = 0.021). Additionally, these patients had more comorbidities including more CVDs at baseline (p = 0.018). Out of 250 patients included in the study, 209 were alive 10 years or more after inclusion, 40 patients were deceased, and one patient was lost to follow-up. Of the 40 non-survivors, 21 died from breast cancer while 19 died of other causes. Eleven survivors had breast cancer recurrence at 10-year follow-up.

**Table 1:** Patient and tumor characteristics at baseline.

		All patients (n = 250)*	10 year survivors (n=209)	10 year non-survivors (n = 40)	p value
Age, mean (SD)	years	58.1 (± 9.8)	57.7 (± 8.7)	60.3 (± 14.2)	0.124
BMI, mean (SD)	kg/m^2^	26.5 (± 4.5)	26.2 (± 4.2)	27.8 (± 5.4)	0.031
Tumor location, n (%)	Left sided Right sided Bilateral	125 (50.0%) 124 (49.6%) 1 (0.4%)	98 (46.9%) 110 (52.6%) 1 (0.5%)	27 (67.5%) 13 (32.5%) 0 (0.0%)	0.055
Histology, n (%)	IDC ILC DCIS/LCIS Other^#^	191 (76.4%) 25 (10.0%) 24 (9.6%) 10 (4.0%)	157 (75.1%) 20 (9.6%) 22 (10.5%) 10 (4.8%)	33 (82.5%) 5 (12.5%) 2 (5.0%) 0 (0.0%)	0.320
Stage, n (%)	0 1 2 3 Unknown	20 (8.0%) 128 (51.2%) 76 (30.4%) 24 (9.6%) 2 (0.8%)	18 (8.6%) 112 (53.6%) 66 (31.6%) 12 (5.7 %) 1 (0.5%)	2 (5.0 %) 15 (37.5 %) 10 (25.0 %) 12 (30.0 %) 1 (2.5%)	<0.001
Tumor size, median (IQR)	cm	1.50 (1.20)	1.50 (1.03)	1.75 (1.75)	0.004
Comorbidities, n (%)	None Cardiovascular Other^¤^	178 (71.2%) 35 (14.0%) 37 (14.8%)	156 (74.6%) 26 (12.4%) 27 (13.0%)	21 (52.5%) 9 (22.5%) 10 (25.0%)	0.018
Esterogen receptor, n (%)	Positive Negative Unknown	190 (76.0 %) 40 (16.0 %) 20 (8.0 %)	160 (76.5 %) 30 (14.3 %) 19 (9.0 %)	30 (75.0 %) 9 (22.5 %) 1 (2.5 %)	0.270
Progesteron receptor, n (%)	Positive Negative Unknown	133 (53.2%) 95 (38.0 %) 22 (8.8 %)	110 (52.6%) 78 (37.3 %) 21 (10.1 %)	23 (57.5%) 16 (40.0 %) 1 (2.5 %)	0.958
HER-2, n (%)	Positive Negative Unknown	49 (19.6%) 177 (70.8 %) 24 (9.6 %)	40 (19.1%) 146 (69.9 %) 23 (11.0 %)	9 (22.5%) 30 (75.0%) 1 (2.5%)	0.829
Lymphatic involvement, n (%)	Positive Negative	74 (29.6%) 176 (70.4%)	56 (26.8%) 153 (73.2%)	18 (45.0%) 22 (55.0%)	0.021
Radiation, n (%)	Local Locoregional	167 (66.8 %) 83 (33.2 %)	144 (68.8 %) 65 (31.1 %)	22 (55.0 %) 18 (45.0 %)	0.087
Chemotherapy, n (%)	No Yes	146 (58.4%) 104 (41.6 %)	125 (60.0 %) 84 (40.2 %)	21 (52.5 %) 19 (47.5 %)	0.390
Endocrine treatment, n (%)	None Tamoxifen AI	114 (45.6 %) 113 (45.2 %) 23 (9.2 %)	99 (47.4 %) 95 (45.4 %) 15 (7.2 %)	14 (35.0 %) 18 (45.0 %) 8 (20.0 %)	0.029
Trastuzumab, n (%)	No Yes	215 (86.0 %) 35 (14.0 %)	179 (85.6 %) 30 (14.4 %)	35 (87.5 %) 5 (12.5 %)	0.766

*One patient was lost-to-follow-up after the first year of sample collection. ^#^Other histologies: tubular (n=4), mucinous (n=3), adenocarcinoma (n=1), metaplastic (n=1), medullary (n=1). ^¤^Other comorbidities: Anxiety/depression (n=9), diabetes (n=4), stroke (n=2), lung disease (n=10), MS Hypothyroidism (n=2), muscles and joints (n=8), narcolepsy (n=1), polio late effects (n=1). AI, aromatase inhibitors.

### Longitudinal Changes After Treatment With Different Treatment Regimens

#### Changes Within Lipoprotein Subfractions

**Figure 2:**
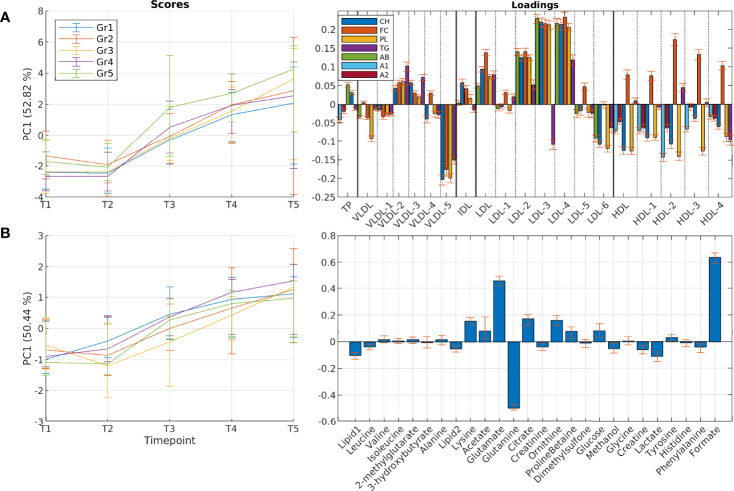
Longitudinal development of circulating lipoprotein subfractions and metabolites in patients receiving different treatment regimens. Results show scores and loadings from RM-ASCA+ analysis including time, treatment, and time-treatment interactions for **(A)** lipoprotein subfractions and **(B)** circulating metabolites. The scores (left) show the overall development for different treatment groups over time (T1-T5), and must be interpreted together with the corresponding loadings (right). An increase in score value means that the treatment groups get increased levels of variables with positive loadings and decreased levels of variables with negative loadings. All treatment groups show clear changes over time both in lipoprotein and metabolite profiles. Treatment is sum coded to detect possible treatment groups deviating from the main trend. A1, apolipoprotein-A1; A2, apolipoprotein-A2; AB, apolipoprotein-B; CH, esterified cholesterol; FC, free cholesterol; Gr, group; PC, principal component; PL, phospholipids; TG, triglycerides.

We detected clear changes in serum lipoprotein parameters and metabolites during the first year after initiation of RT, with similar patterns in all treatment groups (**Figure 2**). For lipoprotein parameters, there was no systematic change evident from before to immediately after RT (T1 and T2, **Figure 2A**), except for decreased levels of the free and esterified cholesterol in LDL6 in univariate testing (**Table S1**). However, measurements taken 3, 6 and 12 months after RT showed clear changes in lipoprotein composition compared to pre-RT samples. Increased levels of total LDL-cholesterol, reductions in total HDL-cholesterol and reduction in total levels of apo-A1 and apo-A2 were evident during the first year after initiation of treatment. When broken down by subfractions, results showed increases in the levels of lipids associated with VLDL2-3 and a decrease in lipids associated with the smallest VLDL particles (VLDL4-5). IDL-cholesterol levels increased, while IDL-triglyceride levels remained constant and decreased at the latest time point (**Table S1**). For LDL subfractions an increase in particle numbers (measured by apo-B levels) and lipids was evident for LDL2-4, however with a significant decrease in LDL3 triglycerides (**Figure 2A** and **Table S1**), while particle numbers and lipid levels for LDL5-6 decreased over time. An interesting pattern was evident for HDL particles, where levels of free cholesterol increased for all subfractions HDL1-4 during the first year. Simultaneously, there was a significant decrease in esterified cholesterol and phospholipid levels for all the HDL subfractions.

#### Differential Changes in Lipoprotein Subfractions Between Treatment Groups

**Figure 3:**
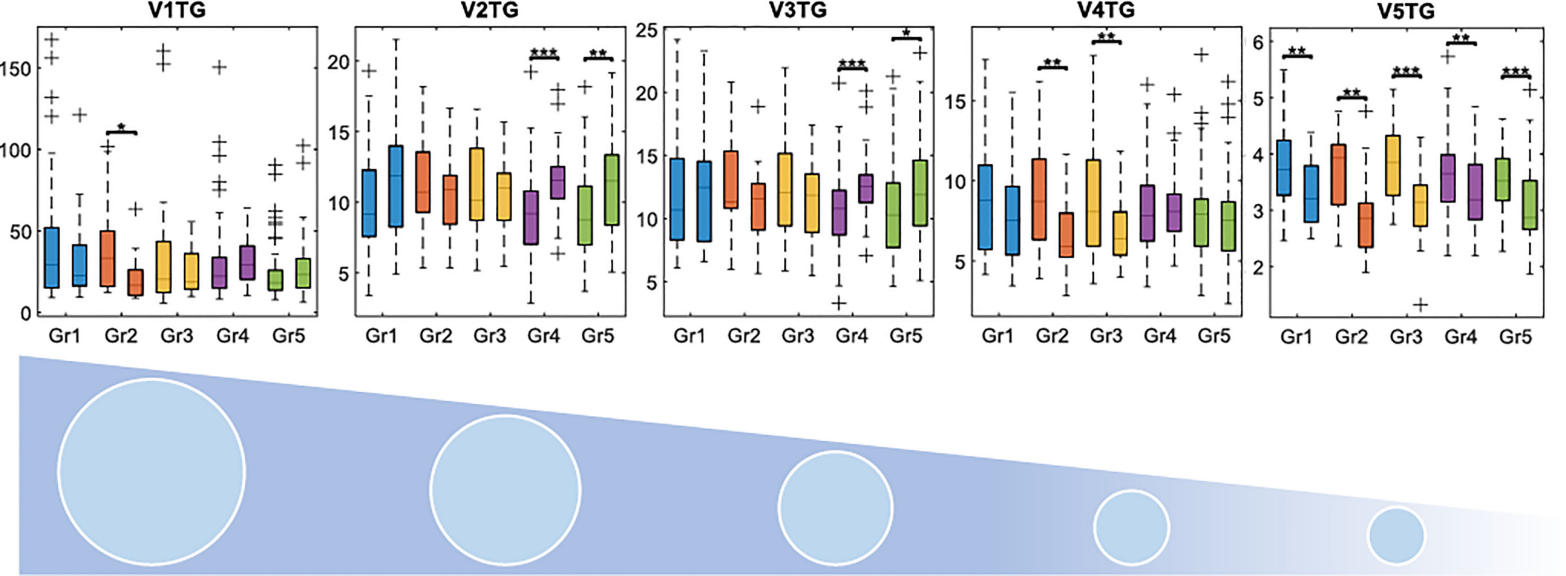
Changes in triglyceride concentrations in the different VLDL subfractions between pre-radiation and one-year follow-up samples. V1 to V5 corresponds to subfractions VLDL1 to VLDL5, where VLDL5 has the highest density. Concentrations are displayed in mg/dL. Gr, group; TG, triglycerides. *adjusted p < 0.05, **adjusted p < 0.01, ***adjusted p < 0.001.

While the overall pattern of change in lipoprotein subfractions was largely shared across different treatment groups, some treatments showed significant differences in lipoprotein changes over time, with the most prominent differences in the triglyceride content (**Table S1**). When comparing VLDLs at the first and last time points (T1 and T5, **Figure 3**), triglyceride levels in VLDL-2 and 3 increased significantly in treatment groups 4 and 5, while increases were only temporary in the remaining treatment groups, and no significant change was apparent in the last time point. VLDL-4 triglyceride levels remained constant, except for treatment groups 2 and 3 where levels decreased significantly. All groups experienced a significant decrease in VLDL-5 triglyceride levels. As groups 4 and 5 were the only groups not receiving chemotherapy before RT, we performed a comparison of patients with and without prior chemotherapy. Results revealed that patients who had received chemotherapy started out with a more dyslipidemic lipid profile at baseline, with higher levels of VLDL-and IDL-related cholesterol and phospholipids, and lower levels of cholesterol and phospholipids in the small HDL4 subfractions, but became more similar to non-recipients over the course of treatment (**Figure S1**).

**Figure 4:**
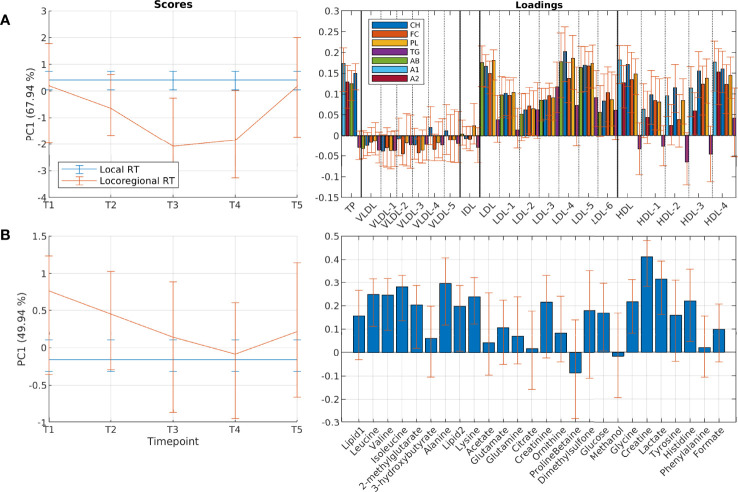
Differences in circulating lipoprotein subfractions and metabolites between patients receiving local and locoregional radiation. Results show scores and loadings from RM-ASCA+ analysis including treatment and time-treatment interactions for **(A)** lipoprotein subfractions and **(B)** circulating metabolites. Treatment is reference coded to local radiation, which therefore is shown as a flat line in the score plot. The scores (left) show the overall development for different treatment groups over time (T1-T5), and must be interpreted together with the corresponding loadings (right). Higher score value means that the group has higher levels of variables with positive loadings and lower levels of variables with negative loadings compared to the group with lower score values. Distinct changes appear in lipoprotein profiles of patients receiving locoregional radiation after 3 and 6 months, however returning to a similar profile as patients that received local radiation after 12 months. A1, apolipoprotein-A1; A2, apolipoprotein-A2; AB, apolipoprotein-B; CH, esterified cholesterol; FC, free cholesterol; PC, principal component; PL, phospholipids; RT, radiotherapy; TG, triglycerides.

Patients who received locoregional RT showed transient reductions in levels of LDL- and HDL lipids compared with patients who received local RT (**Figure 4A**), with total cholesterol levels, total LDL esterified cholesterol, free cholesterol and phospholipid levels, and HDL free cholesterol being significantly lower with locoregional RT, while total triglyceride levels were similar (**Table S2**). This difference started immediately after RT, and progressively became more pronounced until months 6-9, before returning to similar levels as patients receiving local RT after one year.

#### Changes in Circulating Metabolites

For the circulating metabolites, a common multivariate trend was observed across all treatment groups, similarly to the lipoprotein profile (**Figure 2B** and **Table S3**). Levels of lysine, glutamate and formate increased over the course of treatment, while levels of glutamine and lactate decreased. There were no systematic differences in longitudinal changes between the treatment groups. However, patients receiving locoregional RT showed a pattern of higher levels of several metabolites already before RT (**Figure 4B**), with levels of creatinine, glycine, creatine and lactate being significantly higher compared to patients receiving local RT (**Table S4**). Metabolite levels were returned to more similar levels after 3, 6 and 12 months. Similarly, comparison of patients receiving and not receiving chemotherapy before study inclusion showed metabolic differences at baseline, which returned to more similar levels during the first year (**Figure S1**).

### Longitudinal Serum Profiles in Survivors and Non-Survivors

#### Differences Within Lipoprotein Subfractions Between Survivors and Non-Survivors

**Figure 5:**
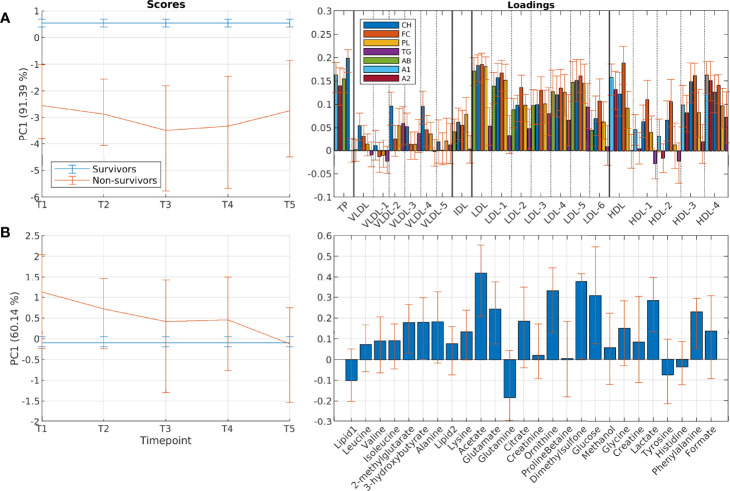
Differences in circulating lipoprotein subfractions and metabolites between 10-year overall survivors and non-survivors. Results show scores and loadings from RM-ASCA+ analysis including group differences and time-group interactions for **(A)** lipoprotein subfractions and **(B)** circulating metabolites. Group variable is reference coded to overall survivors, which therefore is shown as a flat line in the score plot. The scores (left) show the overall development for survival groups over time (T1-T5), and must be interpreted together with the corresponding loadings (right). Distinct differences in the lipoprotein profiles of non-survivors are apparent already at baseline. For instance, survivors have higher score value at all time points for the lipoprotein analysis, which means that the survivors have higher levels of variables with positive loadings and lower levels of variables with negative loadings. A1, apolipoprotein-A1; A2, apolipoprotein-A2; AB, apolipoprotein-B; CH, esterified cholesterol; FC, free cholesterol; PC, principal component; PL, phospholipids; TG, triglycerides.

There were clear differences in lipoprotein profiles between overall survivors and non-survivors at all time points (**Figure 5A**), and the patient groups had significantly different profiles already pre-RT (T1). Non-survivors had lower total cholesterol levels, and lower LDL-cholesterol and HDL-cholesterol, while total triglyceride levels were equal. The same findings were observed when broken down by subfractions, both for larger and smaller subfractions of HDL and LDL. Triglyceride levels of the largest HDL (HDL1) were however higher in non-survivors at baseline, but with a significant decrease over time (**Figure 5A** and **Table S5**).

Separate analysis was performed for breast-cancer specific survival (recurrence and/or death from breast cancer), by excluding patients dying from other causes. Interestingly, the analysis showed similar differences related to breast-cancer specific survival as seen for overall survival (**Figure S2**).

#### Differences in Circulating Metabolites

Non-survivors had different profiles of circulating metabolites compared to 10-year overall survivors (**Figure 5B**). Levels of 2-methylglutarate, 3-hydroxybutyrate, acetate, and glucose were significantly higher at baseline in non-survivors, but decreased to similar levels at later time points (**Figure 5B** and **Table S6**). No differences were observed between survivors and non-survivors when examining breast-cancer specific survival (**Figure S2**).

## Discussion

In this study we reveal distinct changes in serum lipoprotein profiles and levels of circulating metabolites in breast cancer patients during one-year of follow-up after RT. Our results show a development towards an atherogenic lipid profile in all treatment groups after treatment, with decreased levels of esterified cholesterol and increased levels of free cholesterol of all HDL-subfractions, and an increased number of large LDL particles leading to increased overall LDL- cholesterol and triglycerides. At the same time, changes in the glutamine-glutamate metabolism were evident. While some differences were observed between treatment groups, most of the variation was shared across groups despite large differences in treatment regimens. Further, we describe how survivors differ metabolically from non-survivors at different timepoints during adjuvant treatment, finding that the difference in lipid profiles between survivors and non-survivors remain stable over the course of the first year.

All patients in this study had undergone surgical tumor resection followed by RT, and 31% of the patients received RT as the only treatment in addition to surgery. The remaining patients received additional systemic treatment, either before RT (neoadjuvant or adjuvant chemotherapy) or during and after RT (endocrine treatment and trastuzumab), or both. Interestingly, patients experienced similar longitudinal developments despite large differences in treatment regimens. A striking pattern observed across treatment groups was the increase of free cholesterol and decrease of esterified cholesterol within HDL-subfractions during the first year. Cholesterol esterification is an important mechanism to store and transfer cholesterol, and to avoid cellular toxicity from free cholesterol. Free cholesterol is taken up from peripheral tissues by HDL, esterified by lecithin-cholesterol acyltransferase (LCAT), and transported back to the liver for excretion. Previous studies have shown alterations in lipid transport to HDLs in coronary artery disease (CAD) (30), and describe higher levels of free HDL-cholesterol and higher ratio of free-to-esterified HDL-cholesterol in patients with CAD (31). The patterns of increased free cholesterol in HDL-subfractions after treatment will therefore likely contribute to a more atherogenic lipid profile.

The shift towards an atherogenic lipid profile was observed in the cohort as a whole, which suggests that the systemic treatments were not the sole cause of this change. In additional to RT and additional ongoing treatment, other contributing factors could be changes in lifestyle, such as less physical activity, a lifestyle change which is shown to proceed years after breast cancer diagnosis (32). The observed changes in glutamate-glutamine metabolism, with decreased glutamine and increased glutamate levels, may also be explained by such factors. Glutamate and glutamine are non-essential amino acids, and glutamine is the most abundant amino acid found in serum (33). Glutamine can be synthesized from glutamate in a variety of tissues, including liver, muscle, and adipose tissue. Muscular synthesis of glutamine is shown to be reduced during periods with physical inactivity (34). The observed metabolic changes in breast cancer patients could therefore be explained by a combination of ongoing disease and treatment, as well as potentially reduced activity levels, leading to lower muscular intake of glutamate, and in turn lower glutamine synthesis. Importantly, this switch in glutamine-glutamate metabolism could be predictive of a worsening in metabolic health, as high glutamate and low glutamine-to-glutamate ratio has been associated with type 2 diabetes (35). Furthermore, reductions in physical activity levels may also explain the increased levels of free cholesterol in HDL, as LCAT levels are shown to be increased by physical exercise (36). Several studies describe increased mortality in breast cancer survivors compared to the general population, and in particular increased risk of death from CVDs (12–14, 37–39). Although a study on a large cohort of breast cancer survivors concluded no increased risk for heart-specific mortality (40), that study did not adjust for baseline risk factors, and studies adjusting for risk factors show increases in mortality from CVDs after treatment for breast cancer (12–14). Suggested mechanisms include changes in risk factors such as physical inactivity and obesity, in addition to direct effects from the cancer treatment (19, 41). Weight gain is frequently reported after a breast cancer diagnosis (42, 43), and weight gain during adjuvant treatment for breast cancer has been associated with a worse prognosis (44, 45). We have previously reported distinct metabolic profiles before treatment in patients that gained weight during treatment (22). Results from the current study support that changes in lifestyle may contribute to changes in lipoprotein profiles of breast cancer patients, increasing the risk of CVDs in breast cancer survivors.

Patients who had received chemotherapy showed different serum profiles already at start of RT, with higher levels of most of the measured lipoprotein parameters, especially triglycerides and VLDL-related cholesterol and lipids. This could be attributed to a combination of patient selection for chemotherapy, tumor characteristics and residual effects from recently finished chemotherapy, an effect that has been described in previous studies (22, 46, 47). Patients that received chemotherapy had finished treatment at least three weeks before radiation. Chemotherapy has been shown to have a transiently dyslipidemic effect, with changes becoming less apparent over time (22, 46, 48). In line with this, the lipid profile of chemotherapy recipients became more similar to that of non-recipients over the course of treatment.

Locoregional radiation is provided to patients with lymphatic involvement, entailing a larger radiation dose and a larger radiated area. Prior to radiation, patients that would receive local and locoregional radiation had similar lipid profiles. After six to nine months, locoregional radiation recipients showed lower levels of total cholesterol, LDL-cholesterol, and HDL-cholesterol, while triglyceride levels were unaffected. This was also evident when stratified by subfractions; most of the LDL- and HDL-subfractions were affected, while VLDL-lipids remained unchanged. These differences were temporary, and largely vanished 12 months after radiation. The metabolic impact of different doses and radiation volumes was investigated for head- and neck cancer by Jelonek et al., who found that changes in several phospholipids and sphingolipids correlated with radiation dose and volume (49). Although their study did not include measurement of lipoproteins, the results suggest that metabolic effects from radiation are dose- and tissue volume dependent. A previous study showed decreased levels of total- and LDL-cholesterol immediately after RT for breast cancer patients, suggesting that radiation improved the lipid profile of breast cancer patients (50). Our results show that the dose-dependent lowering of cholesterol after radiation is only temporary, and that patients receiving radiation as the only additional treatment to surgery experience the same atherogenic shift as patients with systemic treatments during the first year. Patients still alive 10 years after study inclusion had a different lipoprotein profile from all-cause non-survivors at all timepoints during the first year. This difference was stable over time, thus treatment did not appear to worsen the lipid profile of non-survivors more than for survivors. Interestingly, the same differences between survivors and non-survivors were evident for breast cancer specific mortality, when excluding patients that died of other causes. Overweight and obesity have been associated with poorer all-cause and breast cancer specific survival (51, 52), and in agreement with this non-survivors had a slightly higher BMI than survivors (p = 0.031). The main differences between survivors and non-survivors were lower levels of total LDL- and HDL-cholesterol in non-survivors, while total triglyceride levels and all VLDL-related lipids were similar. When stratified by subfractions, it became apparent that the reduced levels of free and esterified cholesterol in LDLs were evenly distributed across subfractions, while for HDLs it was the smaller subfractions (HDL3-4) that had the most reduced cholesterol levels in non-survivors. In our cohort, non-survivors had both more advanced cancer and more comorbidities, including CVDs at baseline, which may have impacted their lipoprotein profiles either due to generally poorer health status or by medication use. However, only nine patients reported statin use at baseline in our cohort (self-reported), of which three were non-survivors. Thus, statin use does not explain the decreased cholesterol levels in non-survivors. Previous results on the association between cholesterol levels and breast cancer mortality have been conflicting. A trend towards higher risk of recurrence with increased total cholesterol has been described for early-stage breast cancer patients (53), while an inverse association between the HDL-to-total cholesterol ratio and mortality was found for triple negative breast cancers (54). In contrast to this and in agreement with our finding, a large retrospective study found that hyperlipidemia both reduced the risk of developing breast cancer and was associated with reduced mortality rates (55), while a related study describe reduced mortality in several cancers, including breast cancer, in patients with hyperlipidemia (56).

Most of the field of cardio-oncology is currently oriented towards understanding direct cardiotoxic effects from cancer treatment, such as chemotherapy-related cardiotoxicity or radiation-induced cardiac damage. While these factors remain very important and have seen much progress, we believe our findings also highlight the role of general cardiovascular prevention measures, such as lifestyle changes and management of blood pressure and cholesterol levels, in this patient group. Recent epidemiological studies have shown that a diagnosis of cancer is associated with increased long-term cardiovascular risk in many cancer types, and cancer has been suggested to be included as a risk enhancing factor in risk prediction tools for CVD (38, 57).

The longitudinal study design is a main strength of this study, where repeated sampling during the first year during and after treatment for breast cancer provides a detailed image of changes in serum lipoprotein composition and circulating metabolites after diagnosis. Another strength is the use of a multivariate analysis method designed for longitudinal multivariate data, which also takes into account co-variation between the variables. A limitation is the lack of pre-surgery blood samples and lacking information on physical activity during patient follow-up, and our results should be explored further in a patient cohort where this information is available.

To conclude, in this study we show that irradiated breast cancer patients during the first year after diagnosis develop a more atherogenic lipid profile, with similar development despite large differences in treatment regimens. This change towards an atherogenic lipid profile may contribute to the increased risk of CVDs in breast cancer survivors. Corresponding changes in circulating metabolites indicate that this development may be partly explained by changes in physical activity levels. Non-survivors had a different lipid profile than overall survivors already at study inclusion, with lower levels of both LDL- and HDL-cholesterol, suggesting a generally poorer health status in this group. Our results demonstrate that a detailed lipoprotein characterization give valuable insight into the atherogenic changes in lipid profiles of breast cancer patients that may explain the increased risk of CVD and increased mortality in this population.

## Data Availability Statement

The datasets presented in this study can be found in online repositories. The names of the repository/repositories and accession number(s) can be found at: Metabolomics. The data have been deposited to the EMBL-EBI MetaboLights database (58) with the identifier MTBLS4068. The complete dataset can be accessed here https://www.ebi.ac.uk/metabolights/MTBLS4068.

## Ethics Statement

The studies involving human participants were reviewed and approved by Regional Committee for Medical Research Ethics (REK 2019/13760). The patients/participants provided their written informed consent to participate in this study.

## Author Contributions

Conceptualization: GFG, SL, RJR, TFB. Methodology: GFG, TSM, TA, TFB. Formal analysis: GFG, TSM, MS. Investigation: GFG, TA. Data curation: GFG, SL, RJR. Writing- original draft: GFG, TSM. Writing- Review & Editing: GFG, TSM, MS, SL, TW, TA, RJR, TFB. Visualization: GFG, TSM. All authors contributed to the article and approved the submitted version.

## Funding

This work has been supported by the Norwegian Cancer Society (GFG: 6834362 and 202021), the Joint Research Committee between St. Olavs hospital and the Faculty of Medicine and Health Sciences, NTNU (GFG: 28328), and the Swiss National Foundation (MS: grant P400PM_194492).

## Author Disclaimer

The funding bodies had no role in study design, analysis, or data interpretation.

## Conflict of Interest

The authors declare that the research was conducted in the absence of any commercial or financial relationships that could be construed as a potential conflict of interest.

## Publisher’s Note

All claims expressed in this article are solely those of the authors and do not necessarily represent those of their affiliated organizations, or those of the publisher, the editors and the reviewers. Any product that may be evaluated in this article, or claim that may be made by its manufacturer, is not guaranteed or endorsed by the publisher.
