# Supplementary material for: Longitudinal Changes in Circulating Metabolites and Lipoproteins After Breast Cancer Treatment
Source: Front Oncol. 2022 Jun 16;12:919522. doi: 10.3389/fonc.2022.919522 (PMC9245384; doi:10.3389/fonc.2022.919522)
Supplement: Supplementary file 1 [file DataSheet_1.docx]

Supplementary Material

## **1 Supplementary figures**


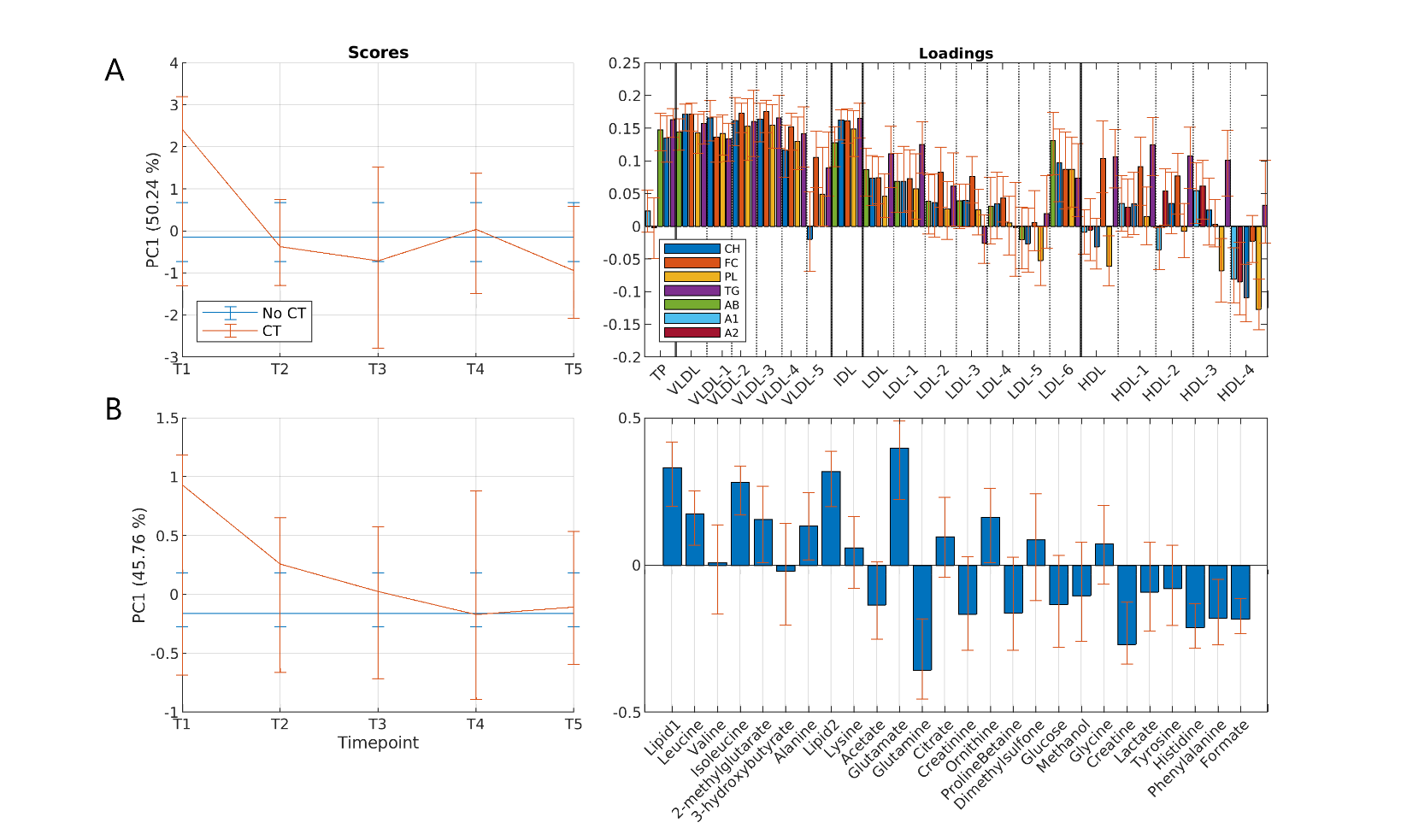


**Figure S1: Longitudinal development of circulating lipoprotein subfractions and metabolites in patients receiving and not receiving chemotherapy**. Results show scores and loadings from RM-ASCA+ analysis of A) lipoprotein subfractions and B) circulating metabolites including treatment and time-treatment interactions to highlight differences between the groups. Treatment is reference coded to patients not receiving chemotherapy, which therefore is shown as a flat line in the score plot. A1, apolipoprotein-A1; A2, aplipoprotein-A2; AB, apolipoprotein-B; CH, esterified cholesterol; CT, chemotherapy; FC, free cholesterol; PC, principal component; PL, phospholipids; TG, triglycerides.


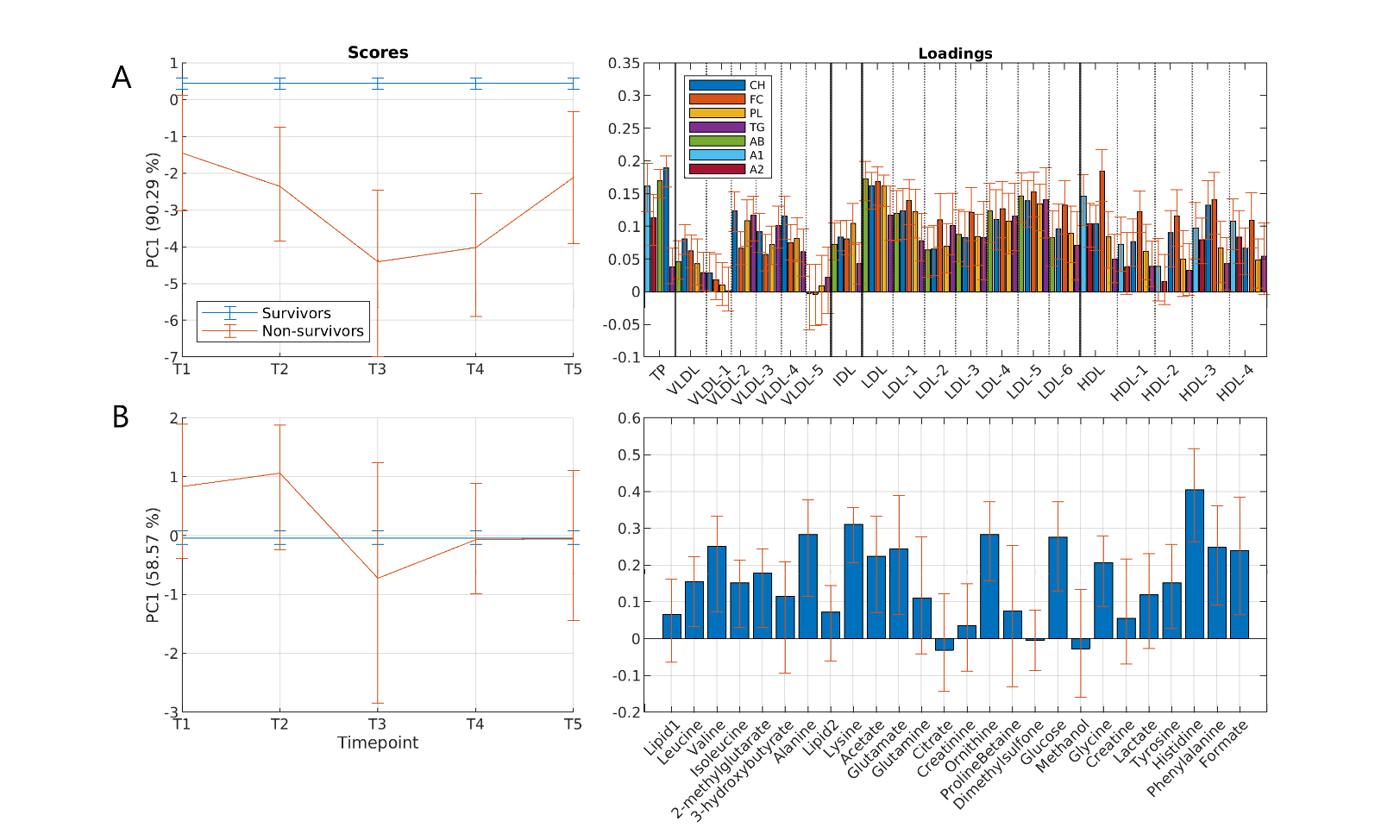


**Figure S2: Differences in circulating lipoprotein subfractions and metabolites between breast cancer survivors and non-survivors, excluding patients that died from other causes.** Results show scores and loadings from RM-ASCA+ analysis including survival group and time-group interactions for A) lipoprotein subfractions and B) circulating metabolites. Group is reference coded to survivors, which therefore is shown as a flat line in the score plot. A1, apolipoprotein-A1; A2, aplipoprotein-A2; AB, apolipoprotein-B; CH, esterified cholesterol; FC, free cholesterol; PC, principal component; PL, phospholipids; TG, triglycerides.

**2 Supplementary tables**

**Table S1: Results from linear mixed model analysis of longitudinal changes in lipoprotein subfractions for different treatment groups.**  The models include main effect for timepoints, treatment group, and the time-group interactions as fixed effects, and a random intercept was included for each patient. The time variable was reference coded to the baseline measurement (T1). Treatment groups were sum coded in statistical analysis to detect possible groups deviating from the average response. q, Benjamini-Hochberg corrected p-values.

|  | **TPA1** | **TPA2** | **TPAB** | **TPCH** | **TPTG** | **VLAB** | **VLCH** | **VLFC** | **VLPL** | **VLTG** | **V1CH** | **V1FC** | **V1PL** |
| --- | --- | --- | --- | --- | --- | --- | --- | --- | --- | --- | --- | --- | --- |
| Intercept | 8.221 | 6.750 | 5.755 | 5.480 | 2.259 | 2.983 | 2.793 | 2.973 | 2.567 | 1.778 | 1.646 | 0.622 | 1.284 |
| q | 0.000 | 0.000 | 0.000 | 0.000 | 0.000 | 0.000 | 0.000 | 0.000 | 0.000 | 0.000 | 0.000 | 0.000 | 0.000 |
| T2 | -0.064 | -0.121 | -0.158 | -0.197 | -0.031 | -0.029 | -0.106 | -0.093 | -0.070 | -0.044 | -0.067 | -0.058 | -0.048 |
| q | 0.829 | 0.431 | 0.075 | 0.027 | 0.663 | 0.657 | 0.121 | 0.137 | 0.370 | 0.518 | 0.279 | 0.412 | 0.480 |
| T3 | 0.283 | 0.328 | 0.158 | 0.175 | -0.008 | -0.054 | 0.028 | -0.083 | -0.153 | 0.003 | -0.057 | -0.043 | -0.034 |
| q | 0.051 | 0.012 | 0.075 | 0.045 | 0.932 | 0.435 | 0.657 | 0.179 | 0.053 | 0.958 | 0.356 | 0.539 | 0.627 |
| T4 | 0.026 | 0.098 | 0.144 | 0.089 | -0.035 | -0.143 | -0.016 | -0.182 | -0.359 | -0.026 | -0.079 | -0.113 | -0.086 |
| q | 0.884 | 0.532 | 0.077 | 0.353 | 0.663 | 0.024 | 0.780 | 0.005 | 0.000 | 0.707 | 0.230 | 0.150 | 0.205 |
| T5 | -0.731 | -0.616 | -0.094 | -0.312 | -0.184 | -0.315 | -0.198 | -0.401 | -0.704 | -0.196 | -0.179 | -0.286 | -0.236 |
| q | 0.000 | 0.000 | 0.312 | 0.001 | 0.022 | 0.000 | 0.013 | 0.000 | 0.000 | 0.013 | 0.031 | 0.000 | 0.003 |
| Group1 | 0.220 | 0.247 | 0.156 | 0.197 | 0.201 | 0.124 | 0.115 | 0.199 | 0.216 | 0.229 | 0.233 | 0.242 | 0.239 |
| q | 0.464 | 0.329 | 0.368 | 0.306 | 0.200 | 0.435 | 0.450 | 0.172 | 0.163 | 0.146 | 0.110 | 0.131 | 0.122 |
| Group2 | -0.120 | -0.070 | 0.187 | 0.134 | 0.181 | 0.164 | 0.214 | 0.154 | 0.146 | 0.159 | 0.184 | 0.156 | 0.186 |
| q | 0.845 | 0.801 | 0.408 | 0.619 | 0.369 | 0.435 | 0.307 | 0.379 | 0.433 | 0.439 | 0.279 | 0.412 | 0.298 |
| Group3 | -0.258 | -0.429 | -0.024 | -0.003 | 0.151 | 0.153 | 0.254 | 0.203 | 0.119 | 0.122 | 0.181 | 0.105 | 0.093 |
| q | 0.464 | 0.116 | 0.879 | 0.987 | 0.371 | 0.435 | 0.152 | 0.189 | 0.446 | 0.489 | 0.230 | 0.510 | 0.568 |
| Group4 | 0.142 | 0.211 | -0.252 | -0.309 | -0.227 | -0.185 | -0.331 | -0.287 | -0.208 | -0.214 | -0.282 | -0.206 | -0.207 |
| q | 0.676 | 0.329 | 0.077 | 0.044 | 0.088 | 0.186 | 0.016 | 0.024 | 0.131 | 0.132 | 0.031 | 0.131 | 0.122 |
| Gr1*T2 | -0.086 | 0.040 | -0.327 | -0.362 | -0.176 | -0.192 | -0.219 | -0.242 | -0.257 | -0.157 | -0.165 | -0.169 | -0.148 |
| q | 0.859 | 0.870 | 0.075 | 0.027 | 0.200 | 0.125 | 0.095 | 0.047 | 0.076 | 0.228 | 0.189 | 0.267 | 0.241 |
| Gr1*T3 | -0.140 | -0.091 | -0.254 | -0.393 | -0.147 | -0.086 | -0.176 | -0.230 | -0.239 | -0.176 | -0.217 | -0.240 | -0.187 |
| q | 0.829 | 0.801 | 0.084 | 0.027 | 0.277 | 0.492 | 0.175 | 0.062 | 0.107 | 0.194 | 0.109 | 0.131 | 0.172 |
| Gr1*T4 | -0.091 | -0.112 | -0.306 | -0.379 | -0.129 | -0.036 | -0.117 | -0.194 | -0.154 | -0.160 | -0.234 | -0.216 | -0.187 |
| q | 0.859 | 0.792 | 0.075 | 0.027 | 0.360 | 0.759 | 0.384 | 0.130 | 0.320 | 0.228 | 0.092 | 0.150 | 0.172 |
| Gr1*T5 | -0.090 | -0.149 | -0.294 | -0.347 | -0.059 | -0.012 | -0.082 | -0.126 | -0.066 | -0.099 | -0.124 | -0.082 | -0.062 |
| q | 0.859 | 0.750 | 0.086 | 0.079 | 0.702 | 0.921 | 0.565 | 0.347 | 0.681 | 0.518 | 0.356 | 0.582 | 0.651 |
| Gr2*T2 | -0.037 | -0.083 | -0.070 | -0.004 | -0.215 | -0.165 | -0.168 | -0.149 | -0.133 | -0.202 | -0.209 | -0.181 | -0.232 |
| q | 0.884 | 0.801 | 0.782 | 0.987 | 0.213 | 0.344 | 0.307 | 0.310 | 0.433 | 0.228 | 0.189 | 0.368 | 0.172 |
| Gr2*T3 | -0.412 | -0.521 | -0.350 | -0.242 | -0.310 | -0.328 | -0.332 | -0.242 | -0.174 | -0.265 | -0.216 | -0.156 | -0.219 |
| q | 0.447 | 0.171 | 0.077 | 0.306 | 0.085 | 0.039 | 0.056 | 0.137 | 0.370 | 0.146 | 0.189 | 0.412 | 0.205 |
| Gr2*T4 | -0.091 | -0.105 | -0.278 | -0.082 | -0.344 | -0.392 | -0.342 | -0.255 | -0.212 | -0.262 | -0.260 | -0.158 | -0.279 |
| q | 0.859 | 0.801 | 0.154 | 0.787 | 0.051 | 0.014 | 0.052 | 0.130 | 0.290 | 0.146 | 0.138 | 0.412 | 0.122 |
| Gr2*T5 | -0.397 | -0.275 | -0.437 | -0.305 | -0.333 | -0.294 | -0.282 | -0.230 | -0.187 | -0.275 | -0.248 | -0.180 | -0.258 |
| q | 0.464 | 0.532 | 0.075 | 0.271 | 0.088 | 0.122 | 0.152 | 0.179 | 0.379 | 0.175 | 0.189 | 0.412 | 0.187 |
| Gr3*T2 | 0.032 | 0.093 | -0.062 | -0.051 | -0.168 | -0.150 | -0.187 | -0.181 | -0.121 | -0.150 | -0.184 | -0.124 | -0.127 |
| q | 0.884 | 0.801 | 0.782 | 0.797 | 0.277 | 0.344 | 0.194 | 0.179 | 0.433 | 0.316 | 0.189 | 0.412 | 0.388 |
| Gr3*T3 | 0.265 | 0.331 | -0.061 | 0.063 | -0.072 | -0.101 | -0.129 | -0.109 | 0.031 | -0.058 | -0.052 | 0.063 | 0.033 |
| q | 0.567 | 0.339 | 0.782 | 0.787 | 0.663 | 0.492 | 0.396 | 0.398 | 0.855 | 0.707 | 0.716 | 0.668 | 0.795 |
| Gr3*T4 | 0.362 | 0.417 | 0.052 | 0.063 | -0.008 | -0.049 | -0.082 | -0.061 | 0.007 | 0.008 | -0.011 | 0.027 | 0.064 |
| q | 0.447 | 0.200 | 0.789 | 0.787 | 0.948 | 0.742 | 0.565 | 0.613 | 0.957 | 0.958 | 0.930 | 0.838 | 0.651 |
| Gr3*T5 | 0.359 | 0.444 | 0.026 | 0.121 | -0.327 | -0.342 | -0.290 | -0.345 | -0.340 | -0.307 | -0.294 | -0.287 | -0.269 |
| q | 0.464 | 0.200 | 0.879 | 0.619 | 0.051 | 0.024 | 0.081 | 0.024 | 0.065 | 0.093 | 0.090 | 0.131 | 0.122 |
| Gr4*T2 | 0.028 | -0.015 | 0.231 | 0.178 | 0.331 | 0.292 | 0.330 | 0.311 | 0.260 | 0.298 | 0.325 | 0.259 | 0.305 |
| q | 0.884 | 0.928 | 0.077 | 0.271 | 0.004 | 0.007 | 0.004 | 0.004 | 0.049 | 0.013 | 0.005 | 0.052 | 0.007 |
| Gr4*T3 | -0.110 | -0.182 | 0.247 | 0.110 | 0.283 | 0.275 | 0.285 | 0.280 | 0.187 | 0.255 | 0.251 | 0.162 | 0.207 |
| q | 0.829 | 0.532 | 0.077 | 0.576 | 0.022 | 0.014 | 0.016 | 0.013 | 0.163 | 0.046 | 0.039 | 0.259 | 0.122 |
| Gr4*T4 | -0.355 | -0.392 | 0.251 | 0.098 | 0.258 | 0.280 | 0.270 | 0.255 | 0.173 | 0.204 | 0.245 | 0.135 | 0.197 |
| q | 0.253 | 0.116 | 0.077 | 0.611 | 0.034 | 0.012 | 0.019 | 0.022 | 0.179 | 0.121 | 0.039 | 0.346 | 0.122 |
| Gr4*T5 | -0.167 | -0.243 | 0.339 | 0.199 | 0.460 | 0.463 | 0.432 | 0.460 | 0.425 | 0.431 | 0.396 | 0.307 | 0.361 |
| q | 0.724 | 0.411 | 0.075 | 0.271 | 0.000 | 0.000 | 0.001 | 0.000 | 0.001 | 0.001 | 0.002 | 0.045 | 0.005 |

Table S1, continued:

|  | **V1TG** | **V2CH** | **V2FC** | **V2PL** | **V2TG** | **V3CH** | **V3FC** | **V3PL** | **V3TG** | **V4CH** | **V4FC** | **V4PL** | **V4TG** |
| --- | --- | --- | --- | --- | --- | --- | --- | --- | --- | --- | --- | --- | --- |
| Intercept | 1.162 | 2.682 | 2.192 | 2.972 | 3.142 | 2.277 | 1.815 | 2.006 | 3.063 | 3.053 | 1.985 | 3.131 | 2.884 |
| q | 0.000 | 0.000 | 0.000 | 0.000 | 0.000 | 0.000 | 0.000 | 0.000 | 0.000 | 0.000 | 0.000 | 0.000 | 0.000 |
| T2 | -0.019 | -0.126 | -0.074 | -0.150 | -0.085 | -0.206 | -0.136 | -0.130 | -0.149 | -0.178 | -0.102 | -0.096 | -0.097 |
| q | 0.770 | 0.142 | 0.290 | 0.105 | 0.359 | 0.008 | 0.061 | 0.086 | 0.079 | 0.035 | 0.154 | 0.180 | 0.183 |
| T3 | -0.052 | 0.190 | 0.163 | 0.366 | 0.468 | 0.177 | 0.076 | 0.159 | 0.256 | -0.079 | 0.097 | -0.032 | -0.059 |
| q | 0.460 | 0.026 | 0.037 | 0.000 | 0.000 | 0.020 | 0.275 | 0.045 | 0.002 | 0.417 | 0.191 | 0.677 | 0.454 |
| T4 | -0.079 | 0.181 | 0.229 | 0.339 | 0.546 | 0.194 | 0.095 | 0.115 | 0.305 | -0.237 | 0.105 | -0.129 | -0.141 |
| q | 0.286 | 0.031 | 0.003 | 0.000 | 0.000 | 0.016 | 0.196 | 0.122 | 0.000 | 0.003 | 0.154 | 0.080 | 0.048 |
| T5 | -0.196 | -0.061 | 0.158 | -0.008 | 0.275 | -0.010 | -0.056 | -0.178 | 0.097 | -0.578 | -0.071 | -0.385 | -0.382 |
| q | 0.019 | 0.484 | 0.064 | 0.963 | 0.007 | 0.892 | 0.411 | 0.045 | 0.306 | 0.000 | 0.389 | 0.000 | 0.000 |
| Group1 | 0.253 | 0.039 | 0.119 | -0.014 | -0.020 | 0.076 | 0.155 | 0.083 | 0.048 | -0.021 | 0.071 | 0.020 | 0.081 |
| q | 0.129 | 0.797 | 0.411 | 0.963 | 0.897 | 0.618 | 0.287 | 0.636 | 0.795 | 0.923 | 0.646 | 0.884 | 0.593 |
| Group2 | 0.135 | 0.248 | 0.229 | 0.351 | 0.337 | 0.200 | 0.186 | 0.223 | 0.221 | 0.137 | 0.175 | 0.148 | 0.119 |
| q | 0.460 | 0.243 | 0.278 | 0.121 | 0.159 | 0.349 | 0.309 | 0.295 | 0.306 | 0.545 | 0.418 | 0.530 | 0.572 |
| Group3 | 0.067 | 0.289 | 0.233 | 0.227 | 0.243 | 0.234 | 0.228 | 0.184 | 0.228 | 0.223 | 0.217 | 0.193 | 0.170 |
| q | 0.684 | 0.142 | 0.214 | 0.212 | 0.224 | 0.215 | 0.196 | 0.295 | 0.258 | 0.273 | 0.234 | 0.322 | 0.413 |
| Group4 | -0.159 | -0.374 | -0.317 | -0.336 | -0.331 | -0.317 | -0.306 | -0.255 | -0.308 | -0.229 | -0.244 | -0.193 | -0.184 |
| q | 0.246 | 0.012 | 0.031 | 0.020 | 0.029 | 0.020 | 0.028 | 0.079 | 0.039 | 0.179 | 0.112 | 0.180 | 0.210 |
| Gr1*T2 | -0.123 | -0.217 | -0.141 | -0.204 | -0.190 | -0.174 | -0.174 | -0.173 | -0.200 | -0.258 | -0.163 | -0.220 | -0.211 |
| q | 0.367 | 0.177 | 0.290 | 0.212 | 0.274 | 0.227 | 0.196 | 0.237 | 0.223 | 0.154 | 0.227 | 0.112 | 0.129 |
| Gr1*T3 | -0.168 | -0.174 | -0.160 | -0.149 | -0.101 | -0.188 | -0.194 | -0.146 | -0.159 | -0.092 | -0.067 | -0.020 | -0.083 |
| q | 0.253 | 0.254 | 0.278 | 0.399 | 0.608 | 0.215 | 0.171 | 0.295 | 0.306 | 0.547 | 0.620 | 0.884 | 0.572 |
| Gr1*T4 | -0.190 | -0.115 | -0.130 | -0.031 | 0.019 | -0.112 | -0.142 | -0.009 | -0.017 | 0.011 | -0.016 | 0.091 | 0.051 |
| q | 0.241 | 0.471 | 0.340 | 0.939 | 0.897 | 0.432 | 0.275 | 0.947 | 0.931 | 0.928 | 0.885 | 0.539 | 0.682 |
| Gr1*T5 | -0.107 | -0.038 | -0.095 | 0.084 | 0.111 | -0.093 | -0.111 | -0.014 | -0.013 | -0.070 | -0.064 | 0.030 | -0.018 |
| q | 0.479 | 0.797 | 0.518 | 0.805 | 0.613 | 0.584 | 0.411 | 0.947 | 0.931 | 0.720 | 0.649 | 0.884 | 0.890 |
| Gr2*T2 | -0.199 | -0.169 | -0.232 | -0.266 | -0.259 | -0.166 | -0.185 | -0.195 | -0.194 | -0.023 | -0.125 | -0.083 | -0.098 |
| q | 0.266 | 0.388 | 0.214 | 0.212 | 0.252 | 0.369 | 0.275 | 0.295 | 0.307 | 0.923 | 0.469 | 0.669 | 0.572 |
| Gr2*T3 | -0.188 | -0.349 | -0.371 | -0.422 | -0.463 | -0.393 | -0.332 | -0.424 | -0.434 | -0.305 | -0.420 | -0.393 | -0.355 |
| q | 0.294 | 0.117 | 0.051 | 0.080 | 0.044 | 0.038 | 0.072 | 0.044 | 0.039 | 0.192 | 0.025 | 0.030 | 0.048 |
| Gr2*T4 | -0.229 | -0.250 | -0.342 | -0.315 | -0.334 | -0.312 | -0.304 | -0.364 | -0.347 | -0.299 | -0.396 | -0.416 | -0.371 |
| q | 0.246 | 0.225 | 0.066 | 0.200 | 0.169 | 0.113 | 0.093 | 0.063 | 0.104 | 0.192 | 0.026 | 0.028 | 0.048 |
| Gr2*T5 | -0.232 | -0.298 | -0.321 | -0.343 | -0.421 | -0.284 | -0.286 | -0.329 | -0.361 | -0.183 | -0.300 | -0.278 | -0.277 |
| q | 0.266 | 0.218 | 0.135 | 0.203 | 0.125 | 0.215 | 0.171 | 0.122 | 0.133 | 0.473 | 0.154 | 0.180 | 0.183 |
| Gr3*T2 | -0.133 | -0.155 | -0.172 | -0.053 | -0.078 | -0.142 | -0.171 | -0.118 | -0.096 | -0.126 | -0.201 | -0.144 | -0.125 |
| q | 0.383 | 0.386 | 0.278 | 0.879 | 0.720 | 0.372 | 0.266 | 0.472 | 0.613 | 0.514 | 0.191 | 0.380 | 0.454 |
| Gr3*T3 | -0.001 | -0.121 | -0.148 | -0.074 | -0.134 | -0.170 | -0.152 | -0.078 | -0.191 | -0.152 | -0.167 | -0.181 | -0.185 |
| q | 0.992 | 0.484 | 0.340 | 0.822 | 0.579 | 0.349 | 0.301 | 0.665 | 0.306 | 0.473 | 0.303 | 0.299 | 0.279 |
| Gr3*T4 | 0.051 | -0.095 | -0.057 | 0.002 | -0.054 | -0.053 | -0.061 | -0.009 | -0.068 | -0.113 | -0.096 | -0.114 | -0.093 |
| q | 0.743 | 0.565 | 0.680 | 0.991 | 0.812 | 0.735 | 0.634 | 0.947 | 0.748 | 0.545 | 0.543 | 0.530 | 0.572 |
| Gr3*T5 | -0.254 | -0.240 | -0.270 | -0.266 | -0.297 | -0.229 | -0.309 | -0.276 | -0.277 | -0.175 | -0.261 | -0.304 | -0.344 |
| q | 0.217 | 0.225 | 0.135 | 0.212 | 0.195 | 0.215 | 0.072 | 0.122 | 0.177 | 0.437 | 0.154 | 0.080 | 0.048 |
| Gr4*T2 | 0.264 | 0.320 | 0.346 | 0.347 | 0.336 | 0.294 | 0.321 | 0.300 | 0.303 | 0.182 | 0.271 | 0.233 | 0.229 |
| q | 0.033 | 0.025 | 0.006 | 0.020 | 0.029 | 0.020 | 0.008 | 0.033 | 0.036 | 0.215 | 0.025 | 0.057 | 0.048 |
| Gr4*T3 | 0.190 | 0.249 | 0.312 | 0.275 | 0.324 | 0.317 | 0.322 | 0.275 | 0.358 | 0.183 | 0.287 | 0.279 | 0.311 |
| q | 0.217 | 0.112 | 0.020 | 0.096 | 0.044 | 0.020 | 0.009 | 0.045 | 0.015 | 0.222 | 0.025 | 0.028 | 0.015 |
| Gr4*T4 | 0.166 | 0.206 | 0.267 | 0.185 | 0.202 | 0.247 | 0.274 | 0.208 | 0.247 | 0.196 | 0.266 | 0.276 | 0.277 |
| q | 0.241 | 0.159 | 0.038 | 0.212 | 0.214 | 0.056 | 0.028 | 0.116 | 0.090 | 0.199 | 0.026 | 0.028 | 0.028 |
| Gr4*T5 | 0.343 | 0.401 | 0.436 | 0.434 | 0.471 | 0.399 | 0.461 | 0.447 | 0.482 | 0.327 | 0.394 | 0.430 | 0.489 |
| q | 0.016 | 0.012 | 0.002 | 0.010 | 0.006 | 0.008 | 0.000 | 0.002 | 0.002 | 0.038 | 0.004 | 0.001 | 0.000 |

Table S1, continued:

|  | **V5CH** | **V5FC** | **V5PL** | **V5TG** | **IDAB** | **IDCH** | **IDFC** | **IDPL** | **IDTG** | **LDAB** | **LDCH** | **LDFC** | **LDPL** |
| --- | --- | --- | --- | --- | --- | --- | --- | --- | --- | --- | --- | --- | --- |
| Intercept | 2.735 | 2.073 | 4.329 | 5.722 | 1.737 | 1.765 | 1.508 | 0.898 | 1.007 | 5.142 | 4.101 | 4.929 | 4.834 |
| q | 0.000 | 0.000 | 0.000 | 0.000 | 0.000 | 0.000 | 0.000 | 0.000 | 0.000 | 0.000 | 0.000 | 0.000 | 0.000 |
| T2 | 0.070 | -0.034 | 0.088 | 0.101 | -0.059 | -0.129 | -0.123 | -0.135 | -0.011 | -0.122 | -0.154 | -0.129 | -0.135 |
| q | 0.583 | 0.799 | 0.509 | 0.252 | 0.449 | 0.067 | 0.075 | 0.100 | 0.906 | 0.218 | 0.064 | 0.121 | 0.136 |
| T3 | -0.678 | -0.546 | -0.624 | -0.429 | 0.107 | 0.170 | 0.131 | 0.200 | 0.033 | 0.192 | 0.283 | 0.415 | 0.268 |
| q | 0.000 | 0.000 | 0.000 | 0.000 | 0.215 | 0.020 | 0.072 | 0.028 | 0.736 | 0.095 | 0.000 | 0.000 | 0.001 |
| T4 | -1.010 | -0.824 | -0.941 | -0.648 | 0.061 | 0.220 | 0.151 | 0.112 | -0.015 | 0.159 | 0.323 | 0.551 | 0.256 |
| q | 0.000 | 0.000 | 0.000 | 0.000 | 0.449 | 0.005 | 0.039 | 0.197 | 0.906 | 0.133 | 0.000 | 0.000 | 0.001 |
| T5 | -1.005 | -0.997 | -1.018 | -0.786 | -0.213 | 0.051 | -0.029 | -0.247 | -0.195 | -0.097 | 0.146 | 0.409 | 0.046 |
| q | 0.000 | 0.000 | 0.000 | 0.000 | 0.012 | 0.497 | 0.695 | 0.013 | 0.023 | 0.420 | 0.143 | 0.000 | 0.728 |
| Group1 | -0.094 | 0.265 | -0.027 | 0.069 | 0.110 | 0.118 | 0.126 | 0.201 | 0.207 | 0.120 | 0.125 | 0.104 | 0.074 |
| q | 0.660 | 0.095 | 0.876 | 0.711 | 0.500 | 0.411 | 0.384 | 0.231 | 0.196 | 0.540 | 0.607 | 0.709 | 0.743 |
| Group2 | -0.185 | -0.031 | -0.094 | 0.011 | 0.198 | 0.227 | 0.210 | 0.189 | 0.207 | 0.147 | 0.128 | 0.098 | 0.109 |
| q | 0.515 | 0.955 | 0.685 | 0.950 | 0.449 | 0.327 | 0.344 | 0.360 | 0.341 | 0.540 | 0.633 | 0.776 | 0.728 |
| Group3 | 0.248 | 0.135 | 0.325 | 0.212 | 0.035 | 0.143 | 0.148 | 0.078 | 0.145 | -0.169 | -0.178 | -0.135 | -0.206 |
| q | 0.336 | 0.500 | 0.139 | 0.309 | 0.868 | 0.410 | 0.384 | 0.655 | 0.427 | 0.495 | 0.459 | 0.648 | 0.432 |
| Group4 | -0.050 | -0.143 | -0.129 | -0.078 | -0.108 | -0.259 | -0.259 | -0.273 | -0.245 | -0.171 | -0.210 | -0.192 | -0.138 |
| q | 0.713 | 0.322 | 0.511 | 0.658 | 0.449 | 0.056 | 0.063 | 0.076 | 0.073 | 0.405 | 0.188 | 0.236 | 0.523 |
| Gr1*T2 | -0.190 | -0.249 | -0.266 | -0.235 | -0.161 | -0.175 | -0.177 | -0.277 | -0.196 | -0.321 | -0.279 | -0.246 | -0.280 |
| q | 0.432 | 0.128 | 0.190 | 0.172 | 0.366 | 0.247 | 0.226 | 0.088 | 0.166 | 0.128 | 0.080 | 0.121 | 0.136 |
| Gr1*T3 | 0.074 | -0.196 | 0.011 | 0.060 | -0.030 | -0.125 | -0.127 | -0.292 | -0.165 | -0.288 | -0.326 | -0.287 | -0.275 |
| q | 0.702 | 0.272 | 0.939 | 0.711 | 0.868 | 0.384 | 0.367 | 0.079 | 0.240 | 0.138 | 0.054 | 0.079 | 0.136 |
| Gr1*T4 | 0.133 | -0.232 | 0.133 | 0.089 | -0.025 | -0.112 | -0.112 | -0.216 | -0.114 | -0.319 | -0.329 | -0.292 | -0.269 |
| q | 0.583 | 0.175 | 0.564 | 0.658 | 0.868 | 0.410 | 0.384 | 0.197 | 0.427 | 0.128 | 0.054 | 0.079 | 0.136 |
| Gr1*T5 | 0.123 | -0.103 | 0.125 | 0.114 | -0.039 | -0.145 | -0.136 | -0.119 | -0.022 | -0.320 | -0.355 | -0.334 | -0.276 |
| q | 0.660 | 0.711 | 0.604 | 0.639 | 0.868 | 0.384 | 0.384 | 0.509 | 0.906 | 0.158 | 0.064 | 0.079 | 0.174 |
| Gr2*T2 | 0.267 | 0.011 | 0.185 | 0.045 | -0.172 | -0.182 | -0.173 | -0.142 | -0.230 | 0.012 | 0.028 | 0.055 | 0.061 |
| q | 0.432 | 0.973 | 0.553 | 0.808 | 0.449 | 0.365 | 0.344 | 0.478 | 0.196 | 0.945 | 0.861 | 0.806 | 0.811 |
| Gr2*T3 | 0.258 | 0.077 | 0.156 | -0.061 | -0.466 | -0.461 | -0.461 | -0.360 | -0.350 | -0.211 | -0.199 | -0.211 | -0.182 |
| q | 0.432 | 0.799 | 0.568 | 0.768 | 0.012 | 0.017 | 0.019 | 0.090 | 0.057 | 0.420 | 0.446 | 0.376 | 0.523 |
| Gr2*T4 | 0.058 | -0.070 | -0.071 | -0.261 | -0.470 | -0.425 | -0.420 | -0.227 | -0.345 | -0.099 | -0.044 | -0.017 | -0.049 |
| q | 0.794 | 0.799 | 0.770 | 0.252 | 0.012 | 0.020 | 0.022 | 0.277 | 0.057 | 0.626 | 0.822 | 0.914 | 0.811 |
| Gr2*T5 | 0.272 | 0.009 | 0.131 | -0.114 | -0.418 | -0.378 | -0.366 | -0.245 | -0.313 | -0.374 | -0.285 | -0.294 | -0.284 |
| q | 0.434 | 0.973 | 0.671 | 0.666 | 0.054 | 0.065 | 0.072 | 0.284 | 0.140 | 0.191 | 0.264 | 0.236 | 0.318 |
| Gr3*T2 | -0.083 | -0.161 | -0.188 | -0.213 | -0.132 | -0.156 | -0.156 | -0.066 | -0.135 | 0.049 | 0.073 | 0.029 | 0.108 |
| q | 0.702 | 0.458 | 0.509 | 0.263 | 0.449 | 0.365 | 0.344 | 0.672 | 0.421 | 0.775 | 0.659 | 0.872 | 0.694 |
| Gr3*T3 | -0.104 | -0.005 | -0.149 | -0.178 | -0.021 | -0.091 | -0.068 | 0.107 | -0.006 | 0.095 | 0.108 | 0.049 | 0.146 |
| q | 0.702 | 0.973 | 0.564 | 0.356 | 0.880 | 0.534 | 0.673 | 0.547 | 0.963 | 0.626 | 0.633 | 0.806 | 0.593 |
| Gr3*T4 | -0.295 | -0.071 | -0.293 | -0.181 | 0.053 | -0.026 | -0.024 | 0.046 | 0.021 | 0.117 | 0.103 | 0.054 | 0.122 |
| q | 0.336 | 0.799 | 0.217 | 0.343 | 0.865 | 0.845 | 0.856 | 0.758 | 0.906 | 0.540 | 0.633 | 0.806 | 0.674 |
| Gr3*T5 | -0.367 | -0.386 | -0.504 | -0.462 | -0.154 | -0.163 | -0.188 | -0.190 | -0.366 | 0.210 | 0.291 | 0.259 | 0.301 |
| q | 0.222 | 0.057 | 0.024 | 0.012 | 0.449 | 0.384 | 0.333 | 0.324 | 0.040 | 0.420 | 0.162 | 0.231 | 0.173 |
| Gr4*T2 | -0.066 | 0.159 | 0.065 | 0.145 | 0.255 | 0.287 | 0.292 | 0.276 | 0.341 | 0.109 | 0.061 | 0.045 | 0.023 |
| q | 0.702 | 0.307 | 0.685 | 0.319 | 0.051 | 0.020 | 0.021 | 0.068 | 0.005 | 0.523 | 0.659 | 0.806 | 0.832 |
| Gr4*T3 | 0.010 | 0.119 | 0.117 | 0.187 | 0.202 | 0.281 | 0.272 | 0.161 | 0.271 | 0.094 | 0.076 | 0.104 | 0.040 |
| q | 0.941 | 0.497 | 0.564 | 0.252 | 0.157 | 0.023 | 0.032 | 0.277 | 0.040 | 0.540 | 0.637 | 0.635 | 0.811 |
| Gr4*T4 | 0.184 | 0.224 | 0.211 | 0.238 | 0.223 | 0.286 | 0.288 | 0.186 | 0.235 | 0.111 | 0.086 | 0.064 | 0.067 |
| q | 0.432 | 0.128 | 0.250 | 0.111 | 0.100 | 0.020 | 0.022 | 0.197 | 0.057 | 0.523 | 0.633 | 0.776 | 0.728 |
| Gr4*T5 | 0.132 | 0.342 | 0.278 | 0.360 | 0.356 | 0.388 | 0.400 | 0.308 | 0.460 | 0.178 | 0.066 | 0.076 | 0.035 |
| q | 0.583 | 0.027 | 0.169 | 0.012 | 0.012 | 0.006 | 0.004 | 0.068 | 0.000 | 0.405 | 0.659 | 0.776 | 0.811 |

Table S1, continued:

|  | **LDTG** | **L1AB** | **L1CH** | **L1FC** | **L1PL** | **L1TG** | **L2AB** | **L2CH** | **L2FC** | **L2PL** | **L2TG** | **L3AB** | **L3CH** |
| --- | --- | --- | --- | --- | --- | --- | --- | --- | --- | --- | --- | --- | --- |
| Intercept | 4.210 | 3.794 | 3.084 | 3.173 | 3.602 | 3.492 | 4.410 | 3.898 | 4.658 | 4.175 | 3.645 | 2.992 | 2.513 |
| q | 0.000 | 0.000 | 0.000 | 0.000 | 0.000 | 0.000 | 0.000 | 0.000 | 0.000 | 0.000 | 0.000 | 0.000 | 0.000 |
| T2 | -0.027 | -0.105 | -0.137 | -0.138 | -0.119 | 0.006 | 0.017 | -0.020 | -0.096 | -0.018 | 0.113 | -0.009 | -0.036 |
| q | 0.878 | 0.254 | 0.098 | 0.246 | 0.232 | 0.978 | 0.876 | 0.900 | 0.463 | 0.900 | 0.238 | 0.911 | 0.894 |
| T3 | 0.285 | -0.045 | -0.059 | 0.083 | -0.062 | 0.033 | 0.253 | 0.203 | 0.263 | 0.224 | 0.266 | 0.622 | 0.567 |
| q | 0.001 | 0.736 | 0.566 | 0.438 | 0.659 | 0.801 | 0.003 | 0.022 | 0.002 | 0.007 | 0.001 | 0.000 | 0.000 |
| T4 | 0.376 | -0.132 | -0.149 | 0.051 | -0.172 | 0.055 | 0.508 | 0.426 | 0.508 | 0.437 | 0.300 | 0.933 | 0.868 |
| q | 0.000 | 0.201 | 0.084 | 0.684 | 0.044 | 0.601 | 0.000 | 0.000 | 0.000 | 0.000 | 0.000 | 0.000 | 0.000 |
| T5 | 0.253 | -0.286 | -0.282 | -0.110 | -0.326 | 0.030 | 0.770 | 0.650 | 0.604 | 0.651 | 0.216 | 1.159 | 1.103 |
| q | 0.007 | 0.000 | 0.000 | 0.423 | 0.000 | 0.840 | 0.000 | 0.000 | 0.000 | 0.000 | 0.025 | 0.000 | 0.000 |
| Group1 | -0.045 | -0.002 | 0.021 | -0.003 | -0.008 | 0.006 | -0.045 | -0.001 | 0.061 | -0.033 | -0.141 | -0.016 | 0.021 |
| q | 0.902 | 0.989 | 0.968 | 0.980 | 0.993 | 0.978 | 0.876 | 0.995 | 0.803 | 0.900 | 0.508 | 0.911 | 0.903 |
| Group2 | 0.270 | 0.045 | 0.042 | 0.063 | 0.046 | 0.169 | 0.068 | 0.040 | 0.069 | 0.039 | 0.212 | 0.141 | 0.108 |
| q | 0.293 | 0.886 | 0.968 | 0.855 | 0.904 | 0.592 | 0.876 | 0.900 | 0.816 | 0.900 | 0.430 | 0.696 | 0.879 |
| Group3 | -0.006 | 0.136 | 0.145 | 0.150 | 0.123 | 0.130 | -0.005 | -0.010 | 0.042 | -0.007 | -0.008 | -0.156 | -0.146 |
| q | 0.970 | 0.736 | 0.566 | 0.679 | 0.705 | 0.604 | 0.977 | 0.989 | 0.847 | 0.976 | 0.962 | 0.688 | 0.637 |
| Group4 | -0.016 | -0.155 | -0.227 | -0.229 | -0.171 | -0.072 | -0.112 | -0.152 | -0.216 | -0.131 | 0.076 | -0.101 | -0.138 |
| q | 0.935 | 0.477 | 0.165 | 0.328 | 0.411 | 0.769 | 0.662 | 0.744 | 0.384 | 0.859 | 0.715 | 0.688 | 0.548 |
| Gr1*T2 | -0.124 | -0.259 | -0.286 | -0.246 | -0.276 | -0.152 | -0.035 | -0.074 | -0.096 | -0.066 | -0.118 | -0.024 | -0.029 |
| q | 0.592 | 0.201 | 0.084 | 0.250 | 0.100 | 0.448 | 0.876 | 0.900 | 0.660 | 0.900 | 0.556 | 0.911 | 0.903 |
| Gr1*T3 | 0.173 | -0.105 | -0.193 | -0.156 | -0.126 | 0.119 | 0.062 | -0.042 | -0.064 | -0.004 | 0.228 | -0.120 | -0.190 |
| q | 0.371 | 0.736 | 0.225 | 0.438 | 0.623 | 0.592 | 0.876 | 0.900 | 0.795 | 0.976 | 0.231 | 0.688 | 0.511 |
| Gr1*T4 | 0.110 | -0.204 | -0.253 | -0.183 | -0.194 | 0.038 | -0.038 | -0.108 | -0.151 | -0.072 | 0.218 | -0.117 | -0.175 |
| q | 0.638 | 0.254 | 0.122 | 0.423 | 0.352 | 0.855 | 0.876 | 0.900 | 0.463 | 0.900 | 0.238 | 0.688 | 0.548 |
| Gr1*T5 | 0.094 | -0.246 | -0.282 | -0.245 | -0.217 | 0.004 | -0.235 | -0.291 | -0.311 | -0.234 | 0.049 | -0.260 | -0.298 |
| q | 0.736 | 0.254 | 0.128 | 0.350 | 0.352 | 0.978 | 0.579 | 0.288 | 0.265 | 0.495 | 0.886 | 0.407 | 0.317 |
| Gr2*T2 | -0.254 | 0.122 | 0.140 | 0.120 | 0.130 | -0.173 | 0.034 | 0.070 | 0.018 | 0.069 | -0.123 | -0.040 | -0.026 |
| q | 0.293 | 0.736 | 0.566 | 0.684 | 0.705 | 0.530 | 0.876 | 0.900 | 0.918 | 0.900 | 0.660 | 0.911 | 0.903 |
| Gr2*T3 | -0.656 | -0.052 | 0.003 | -0.100 | -0.041 | -0.490 | -0.193 | -0.087 | -0.211 | -0.104 | -0.530 | -0.314 | -0.251 |
| q | 0.002 | 0.871 | 0.986 | 0.748 | 0.904 | 0.009 | 0.662 | 0.900 | 0.463 | 0.900 | 0.015 | 0.407 | 0.511 |
| Gr2*T4 | -0.707 | -0.054 | 0.021 | -0.034 | -0.044 | -0.599 | -0.182 | -0.066 | -0.145 | -0.094 | -0.663 | -0.193 | -0.104 |
| q | 0.001 | 0.871 | 0.968 | 0.856 | 0.904 | 0.001 | 0.662 | 0.900 | 0.653 | 0.900 | 0.001 | 0.665 | 0.879 |
| Gr2*T5 | -0.701 | -0.031 | 0.029 | -0.075 | -0.010 | -0.460 | -0.195 | -0.110 | -0.249 | -0.128 | -0.547 | -0.292 | -0.222 |
| q | 0.002 | 0.891 | 0.968 | 0.855 | 0.993 | 0.031 | 0.662 | 0.900 | 0.463 | 0.900 | 0.025 | 0.488 | 0.548 |
| Gr3*T2 | -0.030 | -0.094 | -0.075 | -0.092 | -0.060 | -0.127 | -0.063 | -0.044 | -0.097 | -0.039 | -0.043 | 0.064 | 0.070 |
| q | 0.922 | 0.736 | 0.874 | 0.739 | 0.904 | 0.592 | 0.876 | 0.900 | 0.692 | 0.900 | 0.886 | 0.911 | 0.900 |
| Gr3*T3 | -0.144 | -0.090 | -0.039 | -0.040 | -0.036 | -0.185 | -0.194 | -0.139 | -0.211 | -0.140 | -0.030 | 0.035 | 0.055 |
| q | 0.592 | 0.738 | 0.968 | 0.855 | 0.904 | 0.448 | 0.662 | 0.900 | 0.463 | 0.900 | 0.926 | 0.911 | 0.903 |
| Gr3*T4 | 0.112 | -0.110 | -0.132 | -0.120 | -0.109 | -0.049 | -0.073 | -0.078 | -0.037 | -0.069 | 0.081 | 0.060 | 0.040 |
| q | 0.667 | 0.736 | 0.566 | 0.684 | 0.705 | 0.855 | 0.876 | 0.900 | 0.847 | 0.900 | 0.758 | 0.911 | 0.903 |
| Gr3*T5 | -0.045 | -0.034 | -0.005 | 0.039 | -0.001 | -0.230 | 0.169 | 0.192 | 0.193 | 0.188 | -0.010 | 0.284 | 0.291 |
| q | 0.902 | 0.886 | 0.986 | 0.855 | 0.993 | 0.343 | 0.662 | 0.744 | 0.463 | 0.850 | 0.962 | 0.407 | 0.364 |
| Gr4*T2 | 0.267 | 0.063 | 0.050 | 0.056 | 0.043 | 0.269 | 0.056 | 0.026 | 0.086 | 0.024 | 0.173 | 0.033 | 0.014 |
| q | 0.059 | 0.738 | 0.899 | 0.802 | 0.904 | 0.031 | 0.876 | 0.900 | 0.660 | 0.900 | 0.276 | 0.911 | 0.903 |
| Gr4*T3 | 0.336 | 0.053 | 0.032 | 0.042 | 0.029 | 0.316 | 0.145 | 0.097 | 0.148 | 0.094 | 0.170 | 0.162 | 0.133 |
| q | 0.020 | 0.812 | 0.968 | 0.855 | 0.904 | 0.016 | 0.662 | 0.900 | 0.463 | 0.900 | 0.299 | 0.545 | 0.548 |
| Gr4*T4 | 0.327 | 0.193 | 0.180 | 0.143 | 0.183 | 0.399 | 0.225 | 0.188 | 0.170 | 0.186 | 0.288 | 0.154 | 0.130 |
| q | 0.020 | 0.254 | 0.211 | 0.438 | 0.319 | 0.001 | 0.308 | 0.445 | 0.463 | 0.495 | 0.049 | 0.545 | 0.548 |
| Gr4*T5 | 0.399 | 0.192 | 0.152 | 0.146 | 0.149 | 0.456 | 0.118 | 0.083 | 0.148 | 0.072 | 0.350 | 0.017 | -0.018 |
| q | 0.009 | 0.254 | 0.392 | 0.438 | 0.471 | 0.001 | 0.662 | 0.900 | 0.463 | 0.900 | 0.026 | 0.911 | 0.903 |

Table S1, continued:

|  | **L3FC** | **L3PL** | **L3TG** | **L4AB** | **L4CH** | **L4FC** | **L4PL** | **L4TG** | **L5AB** | **L5CH** | **L5FC** | **L5PL** | **L5TG** |
| --- | --- | --- | --- | --- | --- | --- | --- | --- | --- | --- | --- | --- | --- |
| Intercept | 3.560 | 2.906 | 5.524 | 0.858 | 0.759 | 2.067 | 1.007 | 1.512 | 1.874 | 1.518 | 2.532 | 1.767 | 1.707 |
| q | 0.000 | 0.000 | 0.000 | 0.000 | 0.000 | 0.000 | 0.000 | 0.000 | 0.000 | 0.000 | 0.000 | 0.000 | 0.000 |
| T2 | -0.102 | -0.023 | 0.131 | 0.013 | -0.050 | -0.092 | -0.034 | 0.083 | -0.061 | -0.067 | -0.155 | -0.013 | 0.002 |
| q | 0.318 | 0.914 | 0.290 | 0.980 | 0.812 | 0.383 | 0.843 | 0.521 | 0.727 | 0.733 | 0.325 | 0.947 | 0.984 |
| T3 | 0.520 | 0.565 | -0.187 | 0.798 | 0.696 | 0.760 | 0.737 | 0.554 | 0.227 | 0.234 | 0.303 | 0.241 | 0.197 |
| q | 0.000 | 0.000 | 0.067 | 0.000 | 0.000 | 0.000 | 0.000 | 0.000 | 0.202 | 0.148 | 0.018 | 0.119 | 0.224 |
| T4 | 0.836 | 0.845 | -0.417 | 0.971 | 0.891 | 1.007 | 0.886 | 0.644 | 0.000 | 0.024 | 0.252 | 0.022 | 0.034 |
| q | 0.000 | 0.000 | 0.000 | 0.000 | 0.000 | 0.000 | 0.000 | 0.000 | 1.000 | 0.826 | 0.063 | 0.947 | 0.824 |
| T5 | 0.994 | 1.064 | -0.603 | 0.917 | 0.868 | 0.975 | 0.863 | 0.523 | -0.532 | -0.486 | -0.182 | -0.461 | -0.427 |
| q | 0.000 | 0.000 | 0.000 | 0.000 | 0.000 | 0.000 | 0.000 | 0.000 | 0.000 | 0.000 | 0.325 | 0.001 | 0.001 |
| Group1 | 0.085 | -0.022 | -0.082 | -0.024 | -0.006 | 0.063 | -0.010 | -0.247 | 0.061 | 0.099 | 0.110 | 0.042 | -0.130 |
| q | 0.867 | 0.950 | 0.822 | 0.980 | 0.989 | 0.773 | 0.945 | 0.257 | 0.883 | 0.733 | 0.651 | 0.947 | 0.656 |
| Group2 | 0.077 | 0.112 | 0.055 | 0.215 | 0.173 | 0.230 | 0.167 | 0.255 | 0.141 | 0.113 | 0.117 | 0.115 | 0.304 |
| q | 0.874 | 0.822 | 0.869 | 0.639 | 0.797 | 0.378 | 0.640 | 0.369 | 0.727 | 0.733 | 0.687 | 0.843 | 0.457 |
| Group3 | -0.069 | -0.152 | -0.194 | -0.374 | -0.354 | -0.359 | -0.401 | -0.186 | -0.475 | -0.501 | -0.421 | -0.527 | -0.198 |
| q | 0.874 | 0.677 | 0.476 | 0.163 | 0.247 | 0.079 | 0.087 | 0.472 | 0.093 | 0.057 | 0.099 | 0.036 | 0.654 |
| Group4 | -0.204 | -0.106 | 0.211 | 0.057 | 0.034 | -0.047 | 0.082 | 0.217 | 0.112 | 0.091 | 0.023 | 0.145 | 0.076 |
| q | 0.318 | 0.679 | 0.290 | 0.931 | 0.989 | 0.777 | 0.721 | 0.257 | 0.727 | 0.733 | 0.864 | 0.603 | 0.714 |
| Gr1*T2 | -0.108 | -0.008 | -0.228 | 0.004 | -0.002 | -0.054 | 0.023 | 0.027 | -0.176 | -0.182 | -0.176 | -0.131 | -0.178 |
| q | 0.709 | 0.953 | 0.290 | 0.980 | 0.989 | 0.777 | 0.926 | 0.874 | 0.727 | 0.613 | 0.527 | 0.765 | 0.656 |
| Gr1*T3 | -0.247 | -0.140 | 0.203 | -0.223 | -0.327 | -0.241 | -0.297 | 0.237 | -0.163 | -0.221 | -0.243 | -0.150 | 0.089 |
| q | 0.280 | 0.658 | 0.359 | 0.551 | 0.282 | 0.264 | 0.257 | 0.369 | 0.727 | 0.613 | 0.429 | 0.725 | 0.739 |
| Gr1*T4 | -0.246 | -0.118 | 0.106 | -0.062 | -0.142 | -0.199 | -0.143 | 0.318 | -0.028 | -0.094 | -0.157 | 0.013 | 0.199 |
| q | 0.280 | 0.679 | 0.701 | 0.963 | 0.797 | 0.378 | 0.640 | 0.224 | 0.927 | 0.754 | 0.603 | 0.947 | 0.654 |
| Gr1*T5 | -0.335 | -0.239 | 0.126 | -0.110 | -0.166 | -0.185 | -0.151 | 0.266 | -0.073 | -0.127 | -0.130 | -0.016 | 0.162 |
| q | 0.191 | 0.440 | 0.701 | 0.874 | 0.797 | 0.422 | 0.642 | 0.369 | 0.898 | 0.733 | 0.687 | 0.947 | 0.656 |
| Gr2*T2 | -0.007 | -0.026 | 0.059 | -0.062 | -0.020 | -0.145 | -0.038 | -0.159 | -0.050 | -0.054 | -0.041 | -0.049 | -0.173 |
| q | 0.966 | 0.950 | 0.869 | 0.979 | 0.989 | 0.553 | 0.926 | 0.587 | 0.917 | 0.826 | 0.864 | 0.947 | 0.656 |
| Gr2*T3 | -0.257 | -0.268 | -0.141 | -0.481 | -0.356 | -0.462 | -0.367 | -0.622 | -0.282 | -0.234 | -0.262 | -0.249 | -0.477 |
| q | 0.318 | 0.440 | 0.701 | 0.163 | 0.468 | 0.077 | 0.257 | 0.027 | 0.650 | 0.613 | 0.484 | 0.603 | 0.224 |
| Gr2*T4 | -0.061 | -0.143 | -0.307 | -0.285 | -0.131 | -0.265 | -0.180 | -0.632 | -0.156 | -0.065 | -0.070 | -0.126 | -0.436 |
| q | 0.874 | 0.684 | 0.290 | 0.551 | 0.812 | 0.378 | 0.640 | 0.027 | 0.727 | 0.826 | 0.864 | 0.843 | 0.276 |
| Gr2*T5 | -0.281 | -0.242 | -0.297 | -0.468 | -0.372 | -0.474 | -0.323 | -0.595 | -0.355 | -0.270 | -0.395 | -0.339 | -0.558 |
| q | 0.318 | 0.658 | 0.359 | 0.260 | 0.493 | 0.097 | 0.424 | 0.071 | 0.626 | 0.613 | 0.365 | 0.578 | 0.224 |
| Gr3*T2 | 0.034 | 0.080 | 0.048 | 0.109 | 0.119 | 0.206 | 0.178 | 0.119 | 0.239 | 0.268 | 0.220 | 0.292 | 0.153 |
| q | 0.939 | 0.865 | 0.869 | 0.874 | 0.812 | 0.383 | 0.640 | 0.647 | 0.650 | 0.612 | 0.484 | 0.496 | 0.656 |
| Gr3*T3 | -0.023 | 0.059 | 0.088 | 0.209 | 0.162 | 0.176 | 0.270 | 0.039 | 0.382 | 0.427 | 0.269 | 0.451 | 0.227 |
| q | 0.963 | 0.914 | 0.822 | 0.666 | 0.797 | 0.422 | 0.424 | 0.874 | 0.377 | 0.266 | 0.459 | 0.202 | 0.654 |
| Gr3*T4 | 0.012 | 0.053 | 0.055 | 0.183 | 0.103 | 0.305 | 0.219 | 0.199 | 0.383 | 0.391 | 0.333 | 0.414 | 0.229 |
| q | 0.966 | 0.914 | 0.869 | 0.680 | 0.814 | 0.215 | 0.530 | 0.500 | 0.377 | 0.295 | 0.332 | 0.226 | 0.654 |
| Gr3*T5 | 0.253 | 0.301 | 0.032 | 0.294 | 0.264 | 0.437 | 0.365 | 0.164 | 0.422 | 0.455 | 0.456 | 0.487 | 0.180 |
| q | 0.318 | 0.339 | 0.926 | 0.551 | 0.636 | 0.077 | 0.257 | 0.586 | 0.377 | 0.266 | 0.194 | 0.202 | 0.656 |
| Gr4*T2 | 0.042 | 0.007 | -0.002 | 0.007 | -0.029 | 0.010 | -0.054 | 0.039 | -0.040 | -0.052 | -0.045 | -0.078 | 0.098 |
| q | 0.874 | 0.953 | 0.984 | 0.980 | 0.989 | 0.940 | 0.843 | 0.874 | 0.917 | 0.826 | 0.864 | 0.843 | 0.701 |
| Gr4*T3 | 0.189 | 0.131 | -0.002 | 0.126 | 0.100 | 0.134 | 0.056 | 0.185 | -0.128 | -0.158 | -0.038 | -0.167 | 0.040 |
| q | 0.318 | 0.658 | 0.984 | 0.719 | 0.812 | 0.422 | 0.843 | 0.428 | 0.727 | 0.613 | 0.864 | 0.603 | 0.877 |
| Gr4*T4 | 0.132 | 0.127 | 0.152 | 0.010 | -0.021 | -0.010 | -0.030 | 0.121 | -0.232 | -0.263 | -0.180 | -0.273 | -0.025 |
| q | 0.501 | 0.658 | 0.476 | 0.980 | 0.989 | 0.940 | 0.926 | 0.586 | 0.593 | 0.405 | 0.484 | 0.350 | 0.915 |
| Gr4*T5 | 0.054 | -0.034 | 0.140 | -0.151 | -0.199 | -0.155 | -0.275 | 0.037 | -0.118 | -0.181 | -0.140 | -0.199 | 0.126 |
| q | 0.874 | 0.943 | 0.569 | 0.680 | 0.636 | 0.422 | 0.257 | 0.874 | 0.727 | 0.613 | 0.618 | 0.603 | 0.656 |

Table S1, continued:

|  | **L6AB** | **L6CH** | **L6FC** | **L6PL** | **L6TG** | **HDA1** | **HDA2** | **HDCH** | **HDFC** | **HDPL** | **HDTG** | **H1A1** | **H1A2** |
| --- | --- | --- | --- | --- | --- | --- | --- | --- | --- | --- | --- | --- | --- |
| Intercept | 3.234 | 3.114 | 3.857 | 3.145 | 4.170 | 7.735 | 7.028 | 4.634 | 7.308 | 4.788 | 4.149 | 1.894 | 0.974 |
| q | 0.000 | 0.000 | 0.000 | 0.000 | 0.000 | 0.000 | 0.000 | 0.000 | 0.000 | 0.000 | 0.000 | 0.000 | 0.000 |
| T2 | -0.199 | -0.216 | -0.287 | -0.202 | -0.152 | -0.051 | -0.094 | -0.091 | -0.100 | -0.002 | -0.008 | 0.016 | 0.035 |
| q | 0.033 | 0.038 | 0.003 | 0.067 | 0.281 | 0.882 | 0.583 | 0.497 | 0.467 | 0.993 | 0.915 | 0.934 | 0.911 |
| T3 | -0.264 | -0.247 | 0.010 | -0.267 | -0.049 | 0.200 | 0.243 | -0.082 | 0.372 | 0.010 | 0.177 | 0.051 | 0.090 |
| q | 0.004 | 0.025 | 0.972 | 0.013 | 0.887 | 0.366 | 0.114 | 0.509 | 0.000 | 0.993 | 0.040 | 0.934 | 0.626 |
| T4 | -0.431 | -0.481 | -0.046 | -0.511 | -0.189 | -0.105 | -0.026 | -0.406 | 0.455 | -0.338 | 0.114 | -0.093 | -0.066 |
| q | 0.000 | 0.000 | 0.920 | 0.000 | 0.129 | 0.571 | 0.865 | 0.000 | 0.000 | 0.003 | 0.190 | 0.764 | 0.626 |
| T5 | -0.823 | -0.968 | -0.479 | -1.015 | -0.642 | -0.871 | -0.737 | -1.068 | 0.058 | -0.989 | -0.048 | -0.590 | -0.520 |
| q | 0.000 | 0.000 | 0.000 | 0.000 | 0.000 | 0.000 | 0.000 | 0.000 | 0.714 | 0.000 | 0.632 | 0.000 | 0.000 |
| Group1 | 0.289 | 0.279 | 0.165 | 0.275 | 0.140 | 0.192 | 0.254 | 0.205 | 0.209 | 0.075 | -0.030 | 0.164 | 0.153 |
| q | 0.073 | 0.142 | 0.605 | 0.155 | 0.772 | 0.571 | 0.283 | 0.436 | 0.436 | 0.904 | 0.899 | 0.764 | 0.626 |
| Group2 | 0.035 | 0.022 | 0.039 | 0.001 | 0.084 | -0.156 | -0.077 | -0.205 | -0.008 | -0.179 | 0.220 | -0.037 | 0.048 |
| q | 0.871 | 0.980 | 0.972 | 0.994 | 0.887 | 0.748 | 0.842 | 0.497 | 0.967 | 0.819 | 0.352 | 0.934 | 0.950 |
| Group3 | 0.072 | -0.004 | 0.025 | -0.018 | -0.052 | -0.299 | -0.438 | -0.228 | 0.025 | -0.206 | 0.115 | -0.025 | -0.154 |
| q | 0.785 | 0.982 | 0.972 | 0.950 | 0.975 | 0.450 | 0.114 | 0.436 | 0.967 | 0.643 | 0.611 | 0.934 | 0.626 |
| Group4 | -0.239 | -0.208 | -0.246 | -0.174 | -0.015 | 0.200 | 0.236 | 0.117 | -0.161 | 0.238 | 0.045 | 0.053 | 0.139 |
| q | 0.086 | 0.217 | 0.190 | 0.390 | 0.976 | 0.558 | 0.249 | 0.509 | 0.478 | 0.454 | 0.808 | 0.934 | 0.626 |
| Gr1*T2 | -0.293 | -0.310 | -0.240 | -0.308 | -0.218 | -0.069 | 0.034 | -0.169 | -0.222 | -0.070 | 0.014 | -0.125 | -0.010 |
| q | 0.093 | 0.142 | 0.423 | 0.155 | 0.477 | 0.882 | 0.868 | 0.497 | 0.395 | 0.904 | 0.915 | 0.934 | 0.991 |
| Gr1*T3 | -0.334 | -0.392 | -0.332 | -0.362 | -0.122 | -0.082 | -0.081 | -0.221 | -0.247 | -0.002 | 0.268 | -0.047 | 0.024 |
| q | 0.073 | 0.052 | 0.163 | 0.093 | 0.887 | 0.882 | 0.842 | 0.436 | 0.335 | 0.993 | 0.090 | 0.934 | 0.983 |
| Gr1*T4 | -0.409 | -0.441 | -0.346 | -0.405 | -0.252 | -0.077 | -0.116 | -0.235 | -0.251 | 0.002 | 0.268 | -0.052 | -0.006 |
| q | 0.028 | 0.035 | 0.163 | 0.067 | 0.384 | 0.882 | 0.817 | 0.436 | 0.335 | 0.993 | 0.091 | 0.934 | 0.991 |
| Gr1*T5 | -0.221 | -0.250 | -0.252 | -0.194 | 0.025 | -0.059 | -0.159 | -0.143 | -0.095 | 0.111 | 0.226 | -0.027 | -0.063 |
| q | 0.369 | 0.417 | 0.524 | 0.590 | 0.976 | 0.908 | 0.751 | 0.564 | 0.746 | 0.904 | 0.250 | 0.934 | 0.923 |
| Gr2*T2 | -0.047 | 0.015 | 0.022 | 0.029 | -0.093 | 0.012 | -0.081 | 0.174 | -0.021 | 0.155 | -0.368 | 0.008 | -0.103 |
| q | 0.868 | 0.980 | 0.972 | 0.950 | 0.887 | 0.963 | 0.845 | 0.529 | 0.967 | 0.876 | 0.082 | 0.961 | 0.883 |
| Gr2*T3 | 0.053 | 0.131 | 0.019 | 0.149 | -0.006 | -0.323 | -0.473 | 0.123 | -0.199 | -0.036 | -0.727 | -0.079 | -0.260 |
| q | 0.868 | 0.892 | 0.972 | 0.813 | 0.976 | 0.571 | 0.227 | 0.655 | 0.547 | 0.993 | 0.000 | 0.934 | 0.626 |
| Gr2*T4 | 0.125 | 0.240 | 0.250 | 0.234 | 0.023 | -0.025 | -0.107 | 0.328 | 0.027 | 0.142 | -0.860 | 0.023 | -0.173 |
| q | 0.730 | 0.492 | 0.605 | 0.571 | 0.976 | 0.961 | 0.842 | 0.436 | 0.967 | 0.904 | 0.000 | 0.934 | 0.626 |
| Gr2*T5 | -0.167 | -0.098 | -0.185 | -0.109 | -0.248 | -0.319 | -0.266 | -0.004 | -0.410 | -0.110 | -0.561 | -0.291 | -0.289 |
| q | 0.723 | 0.917 | 0.807 | 0.894 | 0.701 | 0.571 | 0.583 | 0.987 | 0.305 | 0.904 | 0.015 | 0.764 | 0.626 |
| Gr3*T2 | -0.117 | -0.050 | -0.039 | -0.046 | -0.010 | 0.074 | 0.099 | 0.091 | -0.126 | 0.064 | -0.098 | -0.075 | -0.071 |
| q | 0.730 | 0.932 | 0.972 | 0.919 | 0.976 | 0.882 | 0.842 | 0.677 | 0.665 | 0.934 | 0.631 | 0.934 | 0.911 |
| Gr3*T3 | -0.048 | 0.088 | 0.007 | 0.080 | -0.113 | 0.234 | 0.341 | 0.219 | -0.059 | 0.226 | -0.131 | 0.106 | 0.172 |
| q | 0.868 | 0.917 | 0.972 | 0.894 | 0.887 | 0.571 | 0.311 | 0.497 | 0.906 | 0.643 | 0.556 | 0.934 | 0.626 |
| Gr3*T4 | 0.003 | 0.072 | 0.048 | 0.083 | 0.099 | 0.385 | 0.427 | 0.250 | 0.099 | 0.274 | 0.188 | 0.211 | 0.268 |
| q | 0.988 | 0.917 | 0.972 | 0.894 | 0.887 | 0.450 | 0.227 | 0.436 | 0.746 | 0.569 | 0.352 | 0.764 | 0.626 |
| Gr3*T5 | -0.163 | -0.031 | 0.104 | -0.049 | -0.119 | 0.390 | 0.416 | 0.317 | 0.150 | 0.237 | -0.149 | 0.158 | 0.180 |
| q | 0.648 | 0.980 | 0.920 | 0.919 | 0.887 | 0.450 | 0.249 | 0.436 | 0.655 | 0.643 | 0.527 | 0.934 | 0.626 |
| Gr4*T2 | 0.213 | 0.123 | 0.102 | 0.118 | 0.147 | -0.029 | -0.028 | -0.139 | 0.134 | -0.115 | 0.343 | 0.053 | 0.115 |
| q | 0.190 | 0.676 | 0.807 | 0.719 | 0.701 | 0.943 | 0.868 | 0.509 | 0.547 | 0.876 | 0.013 | 0.934 | 0.626 |
| Gr4*T3 | 0.083 | -0.049 | -0.006 | -0.044 | 0.076 | -0.152 | -0.207 | -0.268 | 0.120 | -0.219 | 0.361 | -0.061 | -0.033 |
| q | 0.730 | 0.920 | 0.972 | 0.919 | 0.887 | 0.663 | 0.481 | 0.436 | 0.623 | 0.569 | 0.013 | 0.934 | 0.950 |
| Gr4*T4 | 0.068 | -0.065 | -0.114 | -0.063 | 0.028 | -0.386 | -0.388 | -0.371 | -0.142 | -0.349 | 0.244 | -0.190 | -0.164 |
| q | 0.785 | 0.917 | 0.807 | 0.894 | 0.976 | 0.245 | 0.123 | 0.127 | 0.547 | 0.171 | 0.090 | 0.764 | 0.626 |
| Gr4*T5 | 0.316 | 0.192 | 0.122 | 0.193 | 0.243 | -0.223 | -0.217 | -0.225 | 0.007 | -0.248 | 0.268 | 0.027 | -0.002 |
| q | 0.073 | 0.457 | 0.807 | 0.505 | 0.384 | 0.571 | 0.481 | 0.436 | 0.967 | 0.569 | 0.090 | 0.934 | 0.991 |

Table S1, continued:

|  | **H1CH** | **H1FC** | **H1PL** | **H1TG** | **H2A1** | **H2A2** | **H2CH** | **H2FC** | **H2PL** | **H2TG** | **H3A1** | **H3A2** | **H3CH** |
| --- | --- | --- | --- | --- | --- | --- | --- | --- | --- | --- | --- | --- | --- |
| Intercept | 1.949 | 2.911 | 2.056 | 2.181 | 3.569 | 1.033 | 3.639 | 3.367 | 3.655 | 3.236 | 7.016 | 3.547 | 5.911 |
| q | 0.000 | 0.000 | 0.000 | 0.000 | 0.000 | 0.000 | 0.000 | 0.000 | 0.000 | 0.000 | 0.000 | 0.000 | 0.000 |
| T2 | -0.083 | -0.106 | -0.024 | -0.019 | 0.085 | 0.028 | -0.067 | -0.079 | 0.077 | 0.010 | 0.019 | -0.013 | -0.118 |
| q | 0.539 | 0.417 | 0.905 | 0.791 | 0.593 | 0.876 | 0.940 | 0.824 | 0.837 | 0.914 | 0.991 | 0.916 | 0.516 |
| T3 | -0.072 | 0.381 | -0.013 | 0.085 | -0.024 | 0.138 | -0.010 | 0.734 | -0.004 | 0.254 | 0.206 | 0.361 | 0.231 |
| q | 0.539 | 0.000 | 0.905 | 0.442 | 0.913 | 0.447 | 0.983 | 0.000 | 0.991 | 0.001 | 0.231 | 0.001 | 0.172 |
| T4 | -0.273 | 0.478 | -0.207 | 0.048 | -0.373 | -0.066 | -0.271 | 0.956 | -0.329 | 0.284 | -0.066 | 0.212 | 0.006 |
| q | 0.002 | 0.000 | 0.035 | 0.601 | 0.000 | 0.710 | 0.039 | 0.000 | 0.005 | 0.000 | 0.894 | 0.064 | 0.986 |
| T5 | -0.756 | 0.076 | -0.699 | -0.139 | -0.954 | -0.546 | -0.946 | 0.577 | -0.967 | 0.196 | -0.713 | -0.405 | -0.694 |
| q | 0.000 | 0.545 | 0.000 | 0.145 | 0.000 | 0.000 | 0.000 | 0.000 | 0.000 | 0.028 | 0.000 | 0.001 | 0.000 |
| Group1 | 0.201 | 0.222 | 0.141 | 0.086 | 0.149 | 0.181 | 0.228 | 0.098 | 0.104 | -0.081 | 0.229 | 0.164 | 0.216 |
| q | 0.477 | 0.363 | 0.895 | 0.614 | 0.593 | 0.515 | 0.603 | 0.824 | 0.837 | 0.623 | 0.490 | 0.422 | 0.516 |
| Group2 | -0.061 | 0.059 | -0.052 | 0.156 | -0.084 | 0.103 | -0.097 | 0.120 | -0.074 | 0.276 | -0.027 | 0.220 | -0.060 |
| q | 0.909 | 0.811 | 0.905 | 0.501 | 0.836 | 0.770 | 0.940 | 0.824 | 0.851 | 0.210 | 0.991 | 0.419 | 0.986 |
| Group3 | -0.021 | -0.041 | -0.034 | 0.129 | -0.299 | -0.135 | -0.079 | -0.068 | -0.106 | 0.177 | -0.189 | -0.251 | -0.212 |
| q | 0.931 | 0.811 | 0.905 | 0.506 | 0.308 | 0.663 | 0.940 | 0.854 | 0.837 | 0.346 | 0.695 | 0.318 | 0.523 |
| Group4 | -0.032 | -0.125 | 0.062 | 0.007 | 0.329 | 0.122 | 0.065 | -0.025 | 0.253 | 0.002 | 0.179 | 0.122 | 0.091 |
| q | 0.930 | 0.494 | 0.905 | 0.949 | 0.082 | 0.576 | 0.940 | 0.985 | 0.407 | 0.988 | 0.507 | 0.482 | 0.824 |
| Gr1*T2 | -0.222 | -0.153 | -0.145 | -0.048 | 0.001 | 0.042 | -0.135 | 0.066 | 0.002 | 0.087 | 0.013 | 0.082 | -0.021 |
| q | 0.426 | 0.494 | 0.895 | 0.789 | 0.997 | 0.876 | 0.940 | 0.854 | 0.991 | 0.566 | 0.991 | 0.821 | 0.986 |
| Gr1*T3 | -0.255 | -0.228 | -0.099 | 0.129 | 0.093 | -0.003 | -0.128 | 0.003 | 0.125 | 0.351 | -0.002 | 0.037 | -0.117 |
| q | 0.359 | 0.363 | 0.895 | 0.487 | 0.813 | 0.986 | 0.940 | 0.986 | 0.837 | 0.020 | 0.991 | 0.897 | 0.824 |
| Gr1*T4 | -0.253 | -0.229 | -0.104 | 0.075 | 0.088 | 0.005 | -0.137 | -0.003 | 0.103 | 0.313 | -0.008 | 0.094 | -0.111 |
| q | 0.359 | 0.363 | 0.895 | 0.649 | 0.813 | 0.986 | 0.940 | 0.986 | 0.837 | 0.043 | 0.991 | 0.813 | 0.824 |
| Gr1*T5 | -0.173 | -0.138 | -0.029 | 0.050 | 0.164 | -0.049 | -0.005 | -0.022 | 0.203 | 0.259 | 0.096 | 0.057 | 0.032 |
| q | 0.539 | 0.577 | 0.905 | 0.791 | 0.617 | 0.876 | 0.983 | 0.985 | 0.837 | 0.153 | 0.902 | 0.879 | 0.986 |
| Gr2*T2 | 0.078 | -0.084 | 0.059 | -0.295 | -0.050 | -0.126 | 0.083 | -0.108 | 0.048 | -0.368 | -0.129 | -0.272 | -0.012 |
| q | 0.865 | 0.798 | 0.905 | 0.145 | 0.913 | 0.770 | 0.940 | 0.854 | 0.949 | 0.055 | 0.902 | 0.395 | 0.986 |
| Gr2*T3 | 0.138 | -0.223 | 0.042 | -0.503 | -0.270 | -0.328 | -0.075 | -0.542 | -0.177 | -0.717 | -0.544 | -0.718 | -0.390 |
| q | 0.658 | 0.494 | 0.905 | 0.015 | 0.590 | 0.447 | 0.940 | 0.049 | 0.837 | 0.000 | 0.213 | 0.008 | 0.472 |
| Gr2*T4 | 0.211 | 0.019 | 0.106 | -0.624 | -0.200 | -0.268 | 0.052 | -0.177 | -0.130 | -0.883 | -0.354 | -0.525 | -0.082 |
| q | 0.539 | 0.914 | 0.905 | 0.001 | 0.593 | 0.515 | 0.940 | 0.824 | 0.837 | 0.000 | 0.490 | 0.064 | 0.986 |
| Gr2*T5 | -0.091 | -0.403 | -0.166 | -0.473 | -0.327 | -0.343 | -0.283 | -0.521 | -0.329 | -0.562 | -0.511 | -0.596 | -0.369 |
| q | 0.865 | 0.294 | 0.895 | 0.032 | 0.556 | 0.471 | 0.940 | 0.111 | 0.767 | 0.011 | 0.266 | 0.064 | 0.516 |
| Gr3*T2 | -0.022 | -0.081 | -0.040 | -0.145 | 0.039 | -0.129 | -0.051 | -0.130 | -0.092 | -0.160 | -0.084 | -0.020 | 0.004 |
| q | 0.931 | 0.798 | 0.905 | 0.487 | 0.913 | 0.723 | 0.940 | 0.824 | 0.851 | 0.346 | 0.902 | 0.916 | 0.986 |
| Gr3*T3 | 0.175 | 0.070 | 0.141 | -0.156 | 0.320 | 0.190 | 0.110 | -0.026 | 0.111 | -0.228 | 0.211 | 0.313 | 0.230 |
| q | 0.539 | 0.798 | 0.895 | 0.487 | 0.376 | 0.576 | 0.940 | 0.985 | 0.837 | 0.210 | 0.700 | 0.318 | 0.579 |
| Gr3*T4 | 0.161 | 0.152 | 0.193 | 0.160 | 0.385 | 0.291 | 0.305 | 0.174 | 0.353 | 0.200 | 0.398 | 0.453 | 0.349 |
| q | 0.539 | 0.545 | 0.895 | 0.487 | 0.274 | 0.447 | 0.603 | 0.824 | 0.453 | 0.256 | 0.266 | 0.064 | 0.472 |
| Gr3*T5 | 0.157 | 0.214 | 0.147 | -0.152 | 0.216 | 0.105 | 0.143 | 0.253 | 0.086 | -0.146 | 0.113 | 0.271 | 0.280 |
| q | 0.561 | 0.494 | 0.895 | 0.498 | 0.593 | 0.783 | 0.940 | 0.627 | 0.851 | 0.440 | 0.902 | 0.395 | 0.516 |
| Gr4*T2 | 0.010 | 0.130 | 0.013 | 0.322 | 0.012 | 0.143 | 0.005 | 0.095 | 0.021 | 0.360 | 0.136 | 0.156 | 0.006 |
| q | 0.931 | 0.494 | 0.916 | 0.016 | 0.976 | 0.576 | 0.983 | 0.824 | 0.971 | 0.007 | 0.764 | 0.450 | 0.986 |
| Gr4*T3 | -0.132 | 0.056 | -0.103 | 0.322 | -0.155 | -0.033 | -0.116 | 0.083 | -0.110 | 0.356 | -0.006 | -0.047 | -0.108 |
| q | 0.539 | 0.798 | 0.895 | 0.019 | 0.593 | 0.876 | 0.940 | 0.854 | 0.837 | 0.010 | 0.991 | 0.879 | 0.824 |
| Gr4*T4 | -0.174 | -0.139 | -0.193 | 0.222 | -0.271 | -0.143 | -0.237 | -0.191 | -0.255 | 0.249 | -0.221 | -0.216 | -0.302 |
| q | 0.487 | 0.494 | 0.707 | 0.133 | 0.308 | 0.576 | 0.603 | 0.627 | 0.468 | 0.063 | 0.504 | 0.344 | 0.439 |
| Gr4*T5 | 0.015 | 0.040 | -0.022 | 0.334 | -0.150 | 0.043 | -0.062 | -0.063 | -0.097 | 0.257 | -0.075 | -0.052 | -0.222 |
| q | 0.931 | 0.811 | 0.905 | 0.022 | 0.593 | 0.876 | 0.940 | 0.854 | 0.837 | 0.078 | 0.902 | 0.879 | 0.516 |

Table S1, continued:

|  | **H3FC** | **H3PL** | **H3TG** | **H4A1** | **H4A2** | **H4CH** | **H4FC** | **H4PL** | **H4TG** |
| --- | --- | --- | --- | --- | --- | --- | --- | --- | --- |
| Intercept | 3.709 | 4.415 | 3.889 | 7.320 | 6.575 | 4.864 | 4.317 | 4.601 | 6.043 |
| q | 0.000 | 0.000 | 0.000 | 0.000 | 0.000 | 0.000 | 0.000 | 0.000 | 0.000 |
| T2 | -0.059 | 0.063 | -0.003 | -0.092 | -0.130 | -0.086 | -0.171 | -0.008 | -0.069 |
| q | 0.868 | 0.750 | 0.969 | 0.459 | 0.316 | 0.433 | 0.058 | 0.922 | 0.586 |
| T3 | 0.637 | 0.100 | 0.140 | 0.155 | 0.113 | -0.006 | 0.353 | -0.017 | -0.145 |
| q | 0.000 | 0.615 | 0.112 | 0.311 | 0.368 | 0.948 | 0.000 | 0.922 | 0.298 |
| T4 | 0.725 | -0.280 | 0.082 | -0.113 | -0.154 | -0.265 | 0.406 | -0.323 | -0.434 |
| q | 0.000 | 0.067 | 0.371 | 0.440 | 0.275 | 0.015 | 0.000 | 0.001 | 0.000 |
| T5 | 0.334 | -0.984 | -0.052 | -0.567 | -0.612 | -0.626 | 0.210 | -0.697 | -0.696 |
| q | 0.001 | 0.000 | 0.594 | 0.000 | 0.000 | 0.000 | 0.042 | 0.000 | 0.000 |
| Group1 | -0.013 | 0.021 | -0.074 | 0.079 | 0.153 | 0.073 | -0.001 | -0.024 | -0.021 |
| q | 0.928 | 0.904 | 0.656 | 0.665 | 0.403 | 0.679 | 0.992 | 0.922 | 0.883 |
| Group2 | 0.030 | -0.108 | 0.300 | -0.230 | -0.233 | -0.280 | -0.111 | -0.237 | 0.096 |
| q | 0.928 | 0.777 | 0.189 | 0.459 | 0.368 | 0.290 | 0.752 | 0.429 | 0.692 |
| Group3 | -0.195 | -0.248 | 0.144 | -0.381 | -0.469 | -0.373 | -0.178 | -0.328 | -0.057 |
| q | 0.507 | 0.503 | 0.536 | 0.166 | 0.066 | 0.106 | 0.472 | 0.173 | 0.760 |
| Group4 | 0.078 | 0.324 | -0.005 | 0.203 | 0.211 | 0.204 | 0.038 | 0.260 | 0.079 |
| q | 0.868 | 0.141 | 0.969 | 0.372 | 0.311 | 0.272 | 0.900 | 0.167 | 0.692 |
| Gr1*T2 | 0.089 | 0.077 | 0.083 | -0.039 | 0.003 | -0.046 | 0.012 | 0.016 | -0.109 |
| q | 0.868 | 0.823 | 0.594 | 0.844 | 0.985 | 0.802 | 0.967 | 0.922 | 0.643 |
| Gr1*T3 | -0.023 | 0.130 | 0.327 | -0.158 | -0.206 | -0.176 | -0.159 | -0.041 | 0.083 |
| q | 0.928 | 0.750 | 0.039 | 0.516 | 0.368 | 0.433 | 0.472 | 0.922 | 0.692 |
| Gr1*T4 | 0.051 | 0.169 | 0.335 | -0.116 | -0.190 | -0.149 | -0.116 | 0.020 | 0.192 |
| q | 0.928 | 0.640 | 0.039 | 0.664 | 0.368 | 0.471 | 0.630 | 0.922 | 0.489 |
| Gr1*T5 | -0.106 | 0.222 | 0.300 | -0.106 | -0.230 | -0.132 | -0.191 | 0.071 | 0.219 |
| q | 0.868 | 0.615 | 0.117 | 0.665 | 0.368 | 0.570 | 0.472 | 0.922 | 0.489 |
| Gr2*T2 | -0.043 | 0.089 | -0.379 | 0.121 | 0.109 | 0.214 | 0.091 | 0.198 | -0.239 |
| q | 0.928 | 0.823 | 0.063 | 0.665 | 0.653 | 0.433 | 0.814 | 0.556 | 0.489 |
| Gr2*T3 | -0.472 | -0.277 | -0.763 | -0.116 | -0.126 | 0.110 | -0.167 | 0.052 | -0.527 |
| q | 0.072 | 0.615 | 0.000 | 0.665 | 0.636 | 0.679 | 0.595 | 0.922 | 0.039 |
| Gr2*T4 | -0.077 | -0.076 | -0.930 | 0.198 | 0.274 | 0.387 | 0.173 | 0.271 | -0.626 |
| q | 0.928 | 0.837 | 0.000 | 0.523 | 0.368 | 0.163 | 0.595 | 0.419 | 0.010 |
| Gr2*T5 | -0.323 | -0.241 | -0.578 | 0.049 | 0.166 | 0.222 | -0.023 | 0.155 | -0.296 |
| q | 0.422 | 0.640 | 0.014 | 0.844 | 0.592 | 0.471 | 0.967 | 0.757 | 0.489 |
| Gr3*T2 | 0.024 | 0.031 | -0.128 | 0.221 | 0.245 | 0.244 | 0.123 | 0.207 | 0.095 |
| q | 0.928 | 0.904 | 0.536 | 0.459 | 0.368 | 0.311 | 0.630 | 0.450 | 0.692 |
| Gr3*T3 | 0.108 | 0.303 | -0.109 | 0.292 | 0.373 | 0.285 | 0.034 | 0.310 | 0.188 |
| q | 0.868 | 0.503 | 0.594 | 0.372 | 0.231 | 0.277 | 0.942 | 0.262 | 0.527 |
| Gr3*T4 | 0.220 | 0.376 | 0.216 | 0.210 | 0.213 | 0.133 | 0.064 | 0.136 | 0.166 |
| q | 0.507 | 0.426 | 0.256 | 0.459 | 0.368 | 0.570 | 0.884 | 0.729 | 0.568 |
| Gr3*T5 | 0.442 | 0.233 | -0.130 | 0.405 | 0.446 | 0.378 | 0.340 | 0.274 | -0.071 |
| q | 0.072 | 0.615 | 0.562 | 0.232 | 0.169 | 0.157 | 0.156 | 0.390 | 0.760 |
| Gr4*T2 | -0.035 | -0.089 | 0.346 | -0.198 | -0.227 | -0.285 | -0.148 | -0.258 | 0.173 |
| q | 0.928 | 0.777 | 0.014 | 0.411 | 0.311 | 0.125 | 0.472 | 0.184 | 0.489 |
| Gr4*T3 | -0.069 | -0.237 | 0.311 | -0.243 | -0.316 | -0.322 | -0.037 | -0.301 | 0.156 |
| q | 0.912 | 0.503 | 0.037 | 0.359 | 0.169 | 0.106 | 0.900 | 0.165 | 0.489 |
| Gr4*T4 | -0.351 | -0.403 | 0.223 | -0.368 | -0.412 | -0.370 | -0.232 | -0.344 | 0.157 |
| q | 0.051 | 0.141 | 0.117 | 0.129 | 0.066 | 0.057 | 0.169 | 0.092 | 0.489 |
| Gr4*T5 | -0.253 | -0.289 | 0.219 | -0.372 | -0.384 | -0.395 | -0.246 | -0.371 | 0.120 |
| q | 0.282 | 0.482 | 0.167 | 0.156 | 0.126 | 0.057 | 0.173 | 0.092 | 0.611 |

**Table S2: Results from linear mixed model analysis of longitudinal changes in lipoprotein subfractions for local and locoregional radiation.**  The models include main effect for timepoints, treatment group, and the time-group interactions as fixed effects, and a random intercept was included for each patient. The time variable was reference coded to the baseline measurement (T1), and treatment was reference coded with local radiation as the reference group. RT,radiotherapy; q, Benjamini-Hochberg corrected p-values.

|  | **TPA1** | **TPA2** | **TPAB** | **TPCH** | **TPTG** | **VLAB** | **VLCH** | **VLFC** | **VLPL** | **VLTG** | **V1CH** | **V1FC** | **V1PL** |
| --- | --- | --- | --- | --- | --- | --- | --- | --- | --- | --- | --- | --- | --- |
| Intercept | 8.249 | 6.767 | 5.667 | 5.402 | 2.108 | 2.841 | 2.616 | 2.812 | 2.428 | 1.633 | 1.484 | 0.491 | 1.145 |
| q | 0.000 | 0.000 | 0.000 | 0.000 | 0.000 | 0.000 | 0.000 | 0.000 | 0.000 | 0.000 | 0.000 | 0.000 | 0.000 |
| T2 | 0.001 | -0.079 | -0.003 | -0.040 | 0.106 | 0.102 | 0.062 | 0.064 | 0.071 | 0.080 | 0.077 | 0.049 | 0.075 |
| q | 0.996 | 0.534 | 0.968 | 0.610 | 0.278 | 0.217 | 0.370 | 0.367 | 0.413 | 0.340 | 0.413 | 0.598 | 0.460 |
| T3 | 0.509 | 0.563 | 0.413 | 0.418 | 0.132 | 0.085 | 0.201 | 0.076 | -0.039 | 0.137 | 0.059 | 0.041 | 0.057 |
| q | 0.000 | 0.000 | 0.000 | 0.000 | 0.214 | 0.239 | 0.010 | 0.367 | 0.630 | 0.162 | 0.511 | 0.598 | 0.479 |
| T4 | 0.158 | 0.208 | 0.349 | 0.283 | 0.074 | -0.030 | 0.122 | -0.061 | -0.270 | 0.056 | 0.029 | -0.044 | -0.004 |
| q | 0.282 | 0.118 | 0.000 | 0.001 | 0.373 | 0.625 | 0.127 | 0.367 | 0.000 | 0.485 | 0.727 | 0.598 | 0.948 |
| T5 | -0.683 | -0.586 | 0.076 | -0.182 | -0.027 | -0.188 | -0.040 | -0.255 | -0.619 | -0.048 | -0.015 | -0.174 | -0.105 |
| q | 0.000 | 0.000 | 0.427 | 0.062 | 0.717 | 0.038 | 0.589 | 0.002 | 0.000 | 0.571 | 0.837 | 0.110 | 0.460 |
| Radiation | 0.054 | 0.130 | 0.124 | 0.114 | 0.200 | 0.208 | 0.247 | 0.224 | 0.196 | 0.199 | 0.213 | 0.165 | 0.179 |
| q | 0.939 | 0.534 | 0.427 | 0.480 | 0.278 | 0.217 | 0.127 | 0.154 | 0.211 | 0.340 | 0.385 | 0.543 | 0.460 |
| RT*T2 | -0.174 | -0.154 | -0.298 | -0.336 | -0.158 | -0.178 | -0.256 | -0.227 | -0.213 | -0.140 | -0.174 | -0.107 | -0.137 |
| q | 0.535 | 0.534 | 0.037 | 0.023 | 0.278 | 0.217 | 0.074 | 0.116 | 0.179 | 0.340 | 0.385 | 0.598 | 0.460 |
| RT*T3 | -0.486 | -0.481 | -0.438 | -0.468 | -0.159 | -0.152 | -0.197 | -0.208 | -0.185 | -0.165 | -0.131 | -0.121 | -0.111 |
| q | 0.040 | 0.043 | 0.003 | 0.002 | 0.278 | 0.239 | 0.143 | 0.151 | 0.211 | 0.340 | 0.413 | 0.598 | 0.479 |
| RT*T4 | -0.458 | -0.408 | -0.374 | -0.433 | -0.072 | -0.072 | -0.130 | -0.113 | -0.088 | -0.036 | -0.083 | -0.048 | -0.046 |
| q | 0.046 | 0.086 | 0.010 | 0.004 | 0.593 | 0.625 | 0.320 | 0.367 | 0.582 | 0.752 | 0.579 | 0.681 | 0.759 |
| RT*T5 | -0.041 | -0.080 | -0.156 | -0.137 | -0.115 | -0.064 | -0.150 | -0.109 | 0.004 | -0.116 | -0.168 | -0.080 | -0.108 |
| q | 0.945 | 0.714 | 0.406 | 0.461 | 0.449 | 0.625 | 0.320 | 0.367 | 0.974 | 0.485 | 0.413 | 0.598 | 0.479 |

Table S2, continued:

|  | **V1TG** | **V2CH** | **V2FC** | **V2PL** | **V2TG** | **V3CH** | **V3FC** | **V3PL** | **V3TG** | **V4CH** | **V4FC** | **V4PL** | **V4TG** |
| --- | --- | --- | --- | --- | --- | --- | --- | --- | --- | --- | --- | --- | --- |
| Intercept | 1.036 | 2.508 | 2.020 | 2.800 | 2.977 | 2.112 | 1.648 | 1.849 | 2.914 | 2.919 | 1.825 | 2.996 | 2.761 |
| q | 0.000 | 0.000 | 0.000 | 0.000 | 0.000 | 0.000 | 0.000 | 0.000 | 0.000 | 0.000 | 0.000 | 0.000 | 0.000 |
| T2 | 0.088 | 0.049 | 0.074 | 0.012 | 0.071 | -0.047 | 0.012 | 0.010 | -0.004 | -0.021 | 0.045 | 0.038 | 0.018 |
| q | 0.544 | 0.512 | 0.317 | 0.976 | 0.505 | 0.509 | 0.855 | 0.885 | 0.988 | 0.774 | 0.490 | 0.704 | 0.979 |
| T3 | 0.041 | 0.376 | 0.335 | 0.555 | 0.666 | 0.382 | 0.246 | 0.340 | 0.468 | 0.106 | 0.279 | 0.145 | 0.116 |
| q | 0.657 | 0.000 | 0.000 | 0.000 | 0.000 | 0.000 | 0.001 | 0.000 | 0.000 | 0.217 | 0.000 | 0.106 | 0.252 |
| T4 | -0.006 | 0.307 | 0.338 | 0.415 | 0.615 | 0.309 | 0.196 | 0.198 | 0.388 | -0.082 | 0.241 | -0.007 | -0.054 |
| q | 0.931 | 0.000 | 0.000 | 0.000 | 0.000 | 0.000 | 0.012 | 0.019 | 0.000 | 0.338 | 0.001 | 0.923 | 0.606 |
| T5 | -0.056 | 0.076 | 0.339 | 0.080 | 0.393 | 0.154 | 0.115 | -0.052 | 0.237 | -0.494 | 0.100 | -0.280 | -0.278 |
| q | 0.629 | 0.465 | 0.000 | 0.569 | 0.000 | 0.108 | 0.202 | 0.743 | 0.020 | 0.000 | 0.230 | 0.001 | 0.001 |
| Radiation | 0.175 | 0.230 | 0.232 | 0.208 | 0.189 | 0.245 | 0.231 | 0.224 | 0.196 | 0.229 | 0.252 | 0.216 | 0.182 |
| q | 0.544 | 0.168 | 0.184 | 0.225 | 0.276 | 0.108 | 0.156 | 0.187 | 0.245 | 0.155 | 0.124 | 0.189 | 0.305 |
| RT*T2 | -0.106 | -0.294 | -0.193 | -0.261 | -0.232 | -0.270 | -0.207 | -0.208 | -0.222 | -0.325 | -0.229 | -0.224 | -0.166 |
| q | 0.561 | 0.060 | 0.192 | 0.171 | 0.237 | 0.074 | 0.156 | 0.187 | 0.167 | 0.035 | 0.110 | 0.135 | 0.302 |
| RT*T3 | -0.120 | -0.219 | -0.164 | -0.225 | -0.216 | -0.226 | -0.173 | -0.200 | -0.228 | -0.251 | -0.181 | -0.196 | -0.195 |
| q | 0.561 | 0.168 | 0.241 | 0.225 | 0.261 | 0.108 | 0.202 | 0.187 | 0.167 | 0.137 | 0.201 | 0.189 | 0.252 |
| RT*T4 | -0.040 | -0.142 | -0.049 | -0.031 | 0.012 | -0.097 | -0.045 | -0.024 | -0.002 | -0.236 | -0.120 | -0.098 | -0.014 |
| q | 0.807 | 0.410 | 0.695 | 0.976 | 0.936 | 0.496 | 0.778 | 0.885 | 0.988 | 0.140 | 0.337 | 0.586 | 0.982 |
| RT*T5 | -0.132 | -0.118 | -0.192 | 0.002 | -0.031 | -0.184 | -0.164 | -0.068 | -0.086 | -0.052 | -0.188 | -0.039 | -0.003 |
| q | 0.561 | 0.465 | 0.232 | 0.992 | 0.936 | 0.231 | 0.251 | 0.778 | 0.713 | 0.774 | 0.201 | 0.851 | 0.982 |

Table S2, continued:

|  | **V5CH** | **V5FC** | **V5PL** | **V5TG** | **IDAB** | **IDCH** | **IDFC** | **IDPL** | **IDTG** | **LDAB** | **LDCH** | **LDFC** | **LDPL** |
| --- | --- | --- | --- | --- | --- | --- | --- | --- | --- | --- | --- | --- | --- |
| Intercept | 2.752 | 1.978 | 4.278 | 5.630 | 1.601 | 1.606 | 1.353 | 0.762 | 0.851 | 5.131 | 4.110 | 4.932 | 4.865 |
| q | 0.000 | 0.000 | 0.000 | 0.000 | 0.000 | 0.000 | 0.000 | 0.000 | 0.000 | 0.000 | 0.000 | 0.000 | 0.000 |
| T2 | 0.082 | 0.072 | 0.157 | 0.192 | 0.085 | 0.029 | 0.027 | -0.007 | 0.120 | -0.020 | -0.081 | -0.048 | -0.071 |
| q | 0.563 | 0.422 | 0.121 | 0.016 | 0.263 | 0.670 | 0.686 | 0.924 | 0.232 | 0.977 | 0.338 | 0.559 | 0.411 |
| T3 | -0.762 | -0.520 | -0.653 | -0.380 | 0.289 | 0.367 | 0.320 | 0.387 | 0.175 | 0.396 | 0.472 | 0.606 | 0.436 |
| q | 0.000 | 0.000 | 0.000 | 0.000 | 0.000 | 0.000 | 0.000 | 0.000 | 0.049 | 0.000 | 0.000 | 0.000 | 0.000 |
| T4 | -0.970 | -0.731 | -0.873 | -0.553 | 0.219 | 0.383 | 0.310 | 0.241 | 0.086 | 0.327 | 0.462 | 0.680 | 0.396 |
| q | 0.000 | 0.000 | 0.000 | 0.000 | 0.006 | 0.000 | 0.000 | 0.006 | 0.303 | 0.000 | 0.000 | 0.000 | 0.000 |
| T5 | -1.105 | -0.950 | -1.037 | -0.737 | -0.060 | 0.243 | 0.155 | -0.134 | -0.051 | 0.005 | 0.237 | 0.536 | 0.103 |
| q | 0.000 | 0.000 | 0.000 | 0.000 | 0.445 | 0.005 | 0.102 | 0.173 | 0.568 | 0.977 | 0.010 | 0.000 | 0.311 |
| Radiation | -0.033 | 0.143 | 0.055 | 0.130 | 0.234 | 0.237 | 0.231 | 0.201 | 0.202 | 0.004 | -0.037 | -0.015 | -0.061 |
| q | 0.803 | 0.396 | 0.760 | 0.486 | 0.111 | 0.105 | 0.118 | 0.173 | 0.303 | 0.977 | 0.796 | 0.918 | 0.738 |
| RT*T2 | -0.084 | -0.164 | -0.134 | -0.121 | -0.232 | -0.246 | -0.229 | -0.193 | -0.145 | -0.242 | -0.185 | -0.210 | -0.194 |
| q | 0.715 | 0.396 | 0.583 | 0.486 | 0.103 | 0.079 | 0.102 | 0.173 | 0.303 | 0.125 | 0.214 | 0.134 | 0.213 |
| RT*T3 | 0.111 | -0.071 | 0.044 | -0.060 | -0.243 | -0.220 | -0.209 | -0.290 | -0.170 | -0.430 | -0.382 | -0.366 | -0.366 |
| q | 0.669 | 0.652 | 0.768 | 0.644 | 0.103 | 0.105 | 0.118 | 0.075 | 0.303 | 0.005 | 0.009 | 0.009 | 0.011 |
| RT*T4 | -0.066 | -0.126 | -0.065 | -0.072 | -0.213 | -0.187 | -0.179 | -0.203 | -0.068 | -0.397 | -0.333 | -0.304 | -0.362 |
| q | 0.741 | 0.422 | 0.760 | 0.644 | 0.116 | 0.141 | 0.152 | 0.173 | 0.571 | 0.009 | 0.019 | 0.030 | 0.011 |
| RT*T5 | 0.231 | 0.038 | 0.136 | 0.067 | -0.137 | -0.219 | -0.198 | -0.084 | -0.091 | -0.071 | -0.103 | -0.185 | -0.043 |
| q | 0.334 | 0.792 | 0.583 | 0.644 | 0.338 | 0.129 | 0.152 | 0.628 | 0.568 | 0.919 | 0.529 | 0.235 | 0.764 |

Table S2, continued:

|  | **LDTG** | **L1AB** | **L1CH** | **L1FC** | **L1PL** | **L1TG** | **L2AB** | **L2CH** | **L2FC** | **L2PL** | **L2TG** | **L3AB** | **L3CH** |
| --- | --- | --- | --- | --- | --- | --- | --- | --- | --- | --- | --- | --- | --- |
| Intercept | 4.108 | 3.726 | 3.027 | 3.112 | 3.548 | 3.375 | 4.417 | 3.917 | 4.629 | 4.197 | 3.581 | 3.016 | 2.545 |
| q | 0.000 | 0.000 | 0.000 | 0.000 | 0.000 | 0.000 | 0.000 | 0.000 | 0.000 | 0.000 | 0.000 | 0.000 | 0.000 |
| T2 | 0.105 | 0.017 | -0.023 | -0.022 | -0.006 | 0.141 | 0.026 | -0.015 | -0.039 | -0.011 | 0.217 | 0.008 | -0.026 |
| q | 0.241 | 0.798 | 0.726 | 0.734 | 0.931 | 0.078 | 0.892 | 0.933 | 0.688 | 0.888 | 0.012 | 0.923 | 0.783 |
| T3 | 0.498 | 0.075 | 0.041 | 0.208 | 0.040 | 0.195 | 0.344 | 0.277 | 0.424 | 0.295 | 0.409 | 0.744 | 0.681 |
| q | 0.000 | 0.447 | 0.693 | 0.005 | 0.743 | 0.021 | 0.000 | 0.001 | 0.000 | 0.000 | 0.000 | 0.000 | 0.000 |
| T4 | 0.559 | 0.060 | 0.029 | 0.224 | 0.014 | 0.257 | 0.603 | 0.504 | 0.623 | 0.518 | 0.499 | 1.025 | 0.945 |
| q | 0.000 | 0.474 | 0.726 | 0.004 | 0.930 | 0.002 | 0.000 | 0.000 | 0.000 | 0.000 | 0.000 | 0.000 | 0.000 |
| T5 | 0.413 | -0.211 | -0.222 | -0.026 | -0.277 | 0.191 | 0.888 | 0.747 | 0.748 | 0.739 | 0.336 | 1.314 | 1.245 |
| q | 0.000 | 0.021 | 0.012 | 0.734 | 0.001 | 0.047 | 0.000 | 0.000 | 0.000 | 0.000 | 0.001 | 0.000 | 0.000 |
| Radiation | 0.157 | 0.117 | 0.081 | 0.091 | 0.086 | 0.177 | -0.036 | -0.062 | 0.021 | -0.063 | 0.109 | -0.056 | -0.075 |
| q | 0.319 | 0.474 | 0.693 | 0.634 | 0.743 | 0.269 | 0.892 | 0.824 | 0.887 | 0.814 | 0.508 | 0.781 | 0.747 |
| RT*T2 | -0.215 | -0.308 | -0.294 | -0.293 | -0.299 | -0.207 | -0.005 | -0.004 | -0.099 | -0.018 | -0.204 | -0.054 | -0.038 |
| q | 0.182 | 0.021 | 0.026 | 0.023 | 0.026 | 0.132 | 0.971 | 0.977 | 0.596 | 0.888 | 0.201 | 0.781 | 0.783 |
| RT*T3 | -0.235 | -0.233 | -0.204 | -0.226 | -0.206 | -0.144 | -0.065 | -0.068 | -0.203 | -0.066 | -0.168 | -0.155 | -0.151 |
| q | 0.181 | 0.104 | 0.167 | 0.095 | 0.171 | 0.283 | 0.892 | 0.824 | 0.256 | 0.814 | 0.297 | 0.409 | 0.473 |
| RT*T4 | -0.232 | -0.431 | -0.406 | -0.381 | -0.422 | -0.251 | -0.141 | -0.130 | -0.191 | -0.141 | -0.331 | -0.162 | -0.138 |
| q | 0.181 | 0.002 | 0.003 | 0.004 | 0.001 | 0.078 | 0.524 | 0.558 | 0.256 | 0.479 | 0.032 | 0.409 | 0.473 |
| RT*T5 | -0.091 | -0.104 | -0.095 | -0.131 | -0.069 | -0.115 | -0.197 | -0.172 | -0.225 | -0.157 | -0.062 | -0.290 | -0.280 |
| q | 0.567 | 0.474 | 0.693 | 0.448 | 0.743 | 0.403 | 0.398 | 0.490 | 0.256 | 0.479 | 0.688 | 0.135 | 0.144 |

Table S2, continued:

|  | **L3FC** | **L3PL** | **L3TG** | **L4AB** | **L4CH** | **L4FC** | **L4PL** | **L4TG** | **L5AB** | **L5CH** | **L5FC** | **L5PL** | **L5TG** |
| --- | --- | --- | --- | --- | --- | --- | --- | --- | --- | --- | --- | --- | --- |
| Intercept | 3.573 | 2.948 | 5.549 | 0.923 | 0.836 | 2.105 | 1.095 | 1.535 | 1.938 | 1.608 | 2.602 | 1.875 | 1.677 |
| q | 0.000 | 0.000 | 0.000 | 0.000 | 0.000 | 0.000 | 0.000 | 0.000 | 0.000 | 0.000 | 0.000 | 0.000 | 0.000 |
| T2 | -0.066 | -0.018 | 0.194 | 0.016 | -0.059 | -0.074 | -0.052 | 0.119 | -0.041 | -0.067 | -0.133 | -0.024 | 0.093 |
| q | 0.513 | 0.815 | 0.028 | 0.878 | 0.631 | 0.490 | 0.657 | 0.474 | 0.762 | 0.682 | 0.321 | 0.828 | 0.461 |
| T3 | 0.677 | 0.668 | -0.148 | 1.006 | 0.921 | 0.966 | 0.914 | 0.671 | 0.380 | 0.371 | 0.480 | 0.355 | 0.325 |
| q | 0.000 | 0.000 | 0.110 | 0.000 | 0.000 | 0.000 | 0.000 | 0.000 | 0.004 | 0.004 | 0.000 | 0.006 | 0.007 |
| T4 | 0.921 | 0.923 | -0.255 | 1.094 | 1.009 | 1.091 | 0.988 | 0.734 | 0.059 | 0.051 | 0.294 | 0.046 | 0.139 |
| q | 0.000 | 0.000 | 0.006 | 0.000 | 0.000 | 0.000 | 0.000 | 0.000 | 0.762 | 0.728 | 0.018 | 0.828 | 0.341 |
| T5 | 1.156 | 1.185 | -0.626 | 1.042 | 0.991 | 1.096 | 0.935 | 0.512 | -0.572 | -0.538 | -0.186 | -0.536 | -0.425 |
| q | 0.000 | 0.000 | 0.000 | 0.000 | 0.000 | 0.000 | 0.000 | 0.000 | 0.000 | 0.000 | 0.230 | 0.000 | 0.002 |
| Radiation | -0.058 | -0.091 | 0.002 | -0.121 | -0.147 | -0.079 | -0.155 | -0.056 | -0.050 | -0.110 | -0.094 | -0.133 | 0.050 |
| q | 0.695 | 0.639 | 0.989 | 0.553 | 0.469 | 0.636 | 0.433 | 0.715 | 0.762 | 0.682 | 0.647 | 0.593 | 0.751 |
| RT*T2 | -0.080 | -0.039 | -0.185 | -0.038 | -0.025 | -0.057 | -0.028 | -0.101 | -0.106 | -0.051 | -0.099 | -0.053 | -0.212 |
| q | 0.629 | 0.815 | 0.258 | 0.878 | 0.890 | 0.710 | 0.868 | 0.677 | 0.762 | 0.793 | 0.647 | 0.828 | 0.347 |
| RT*T3 | -0.215 | -0.131 | -0.160 | -0.373 | -0.436 | -0.347 | -0.351 | -0.092 | -0.422 | -0.401 | -0.420 | -0.365 | -0.250 |
| q | 0.224 | 0.485 | 0.326 | 0.087 | 0.039 | 0.053 | 0.086 | 0.677 | 0.099 | 0.114 | 0.056 | 0.166 | 0.341 |
| RT*T4 | -0.146 | -0.144 | -0.381 | -0.268 | -0.276 | -0.174 | -0.258 | -0.110 | -0.252 | -0.192 | -0.183 | -0.195 | -0.236 |
| q | 0.442 | 0.485 | 0.019 | 0.243 | 0.233 | 0.391 | 0.229 | 0.677 | 0.437 | 0.675 | 0.454 | 0.593 | 0.341 |
| RT*T5 | -0.271 | -0.236 | 0.146 | -0.169 | -0.180 | -0.186 | -0.116 | 0.193 | 0.137 | 0.132 | 0.077 | 0.183 | 0.150 |
| q | 0.167 | 0.231 | 0.387 | 0.553 | 0.469 | 0.391 | 0.657 | 0.540 | 0.762 | 0.682 | 0.701 | 0.593 | 0.512 |

Table S2, continued:

|  | **L6AB** | **L6CH** | **L6FC** | **L6PL** | **L6TG** | **HDA1** | **HDA2** | **HDCH** | **HDFC** | **HDPL** | **HDTG** | **H1A1** | **H1A2** |
| --- | --- | --- | --- | --- | --- | --- | --- | --- | --- | --- | --- | --- | --- |
| Intercept | 3.138 | 3.048 | 3.821 | 3.092 | 4.097 | 7.794 | 7.045 | 4.710 | 7.269 | 4.861 | 4.005 | 1.867 | 0.923 |
| q | 0.000 | 0.000 | 0.000 | 0.000 | 0.000 | 0.000 | 0.000 | 0.000 | 0.000 | 0.000 | 0.000 | 0.000 | 0.000 |
| T2 | -0.067 | -0.105 | -0.195 | -0.098 | -0.035 | -0.020 | -0.054 | -0.084 | 0.037 | -0.005 | 0.129 | 0.076 | 0.106 |
| q | 0.472 | 0.320 | 0.073 | 0.367 | 0.679 | 0.998 | 0.697 | 0.593 | 0.693 | 0.962 | 0.188 | 0.562 | 0.318 |
| T3 | -0.115 | -0.123 | 0.170 | -0.154 | 0.099 | 0.397 | 0.460 | 0.007 | 0.596 | 0.077 | 0.349 | 0.116 | 0.182 |
| q | 0.292 | 0.320 | 0.126 | 0.213 | 0.385 | 0.002 | 0.000 | 0.945 | 0.000 | 0.650 | 0.000 | 0.376 | 0.093 |
| T4 | -0.350 | -0.419 | -0.005 | -0.458 | -0.126 | 0.000 | 0.087 | -0.363 | 0.565 | -0.288 | 0.290 | -0.046 | 0.023 |
| q | 0.000 | 0.000 | 0.959 | 0.000 | 0.293 | 0.998 | 0.565 | 0.002 | 0.000 | 0.019 | 0.001 | 0.718 | 0.787 |
| T5 | -0.743 | -0.940 | -0.431 | -1.006 | -0.620 | -0.892 | -0.711 | -1.174 | 0.199 | -1.094 | 0.104 | -0.571 | -0.460 |
| q | 0.000 | 0.000 | 0.000 | 0.000 | 0.000 | 0.000 | 0.000 | 0.000 | 0.071 | 0.000 | 0.301 | 0.000 | 0.000 |
| Radiation | 0.140 | 0.100 | 0.034 | 0.080 | 0.139 | 0.007 | 0.132 | -0.056 | 0.057 | -0.035 | 0.249 | 0.056 | 0.143 |
| q | 0.361 | 0.538 | 0.959 | 0.579 | 0.399 | 0.998 | 0.565 | 0.811 | 0.693 | 0.921 | 0.188 | 0.748 | 0.376 |
| RT*T2 | -0.217 | -0.215 | -0.190 | -0.206 | -0.230 | -0.128 | -0.147 | -0.111 | -0.269 | -0.103 | -0.186 | -0.113 | -0.135 |
| q | 0.276 | 0.320 | 0.377 | 0.347 | 0.293 | 0.869 | 0.565 | 0.671 | 0.086 | 0.705 | 0.238 | 0.594 | 0.431 |
| RT*T3 | -0.335 | -0.350 | -0.361 | -0.337 | -0.347 | -0.459 | -0.467 | -0.350 | -0.436 | -0.280 | -0.122 | -0.179 | -0.215 |
| q | 0.066 | 0.089 | 0.073 | 0.113 | 0.073 | 0.064 | 0.051 | 0.120 | 0.008 | 0.251 | 0.376 | 0.419 | 0.283 |
| RT*T4 | -0.171 | -0.202 | -0.163 | -0.191 | -0.167 | -0.433 | -0.420 | -0.347 | -0.285 | -0.352 | -0.204 | -0.226 | -0.280 |
| q | 0.361 | 0.320 | 0.443 | 0.365 | 0.385 | 0.069 | 0.073 | 0.120 | 0.086 | 0.135 | 0.232 | 0.376 | 0.139 |
| RT*T5 | -0.003 | 0.059 | 0.010 | 0.102 | 0.089 | 0.083 | -0.064 | 0.207 | -0.194 | 0.200 | -0.151 | 0.044 | -0.076 |
| q | 0.985 | 0.745 | 0.959 | 0.579 | 0.654 | 0.998 | 0.770 | 0.506 | 0.284 | 0.526 | 0.352 | 0.778 | 0.702 |

Table S2, continued:

|  | **H1CH** | **H1FC** | **H1PL** | **H1TG** | **H2A1** | **H2A2** | **H2CH** | **H2FC** | **H2PL** | **H2TG** | **H3A1** | **H3A2** | **H3CH** |
| --- | --- | --- | --- | --- | --- | --- | --- | --- | --- | --- | --- | --- | --- |
| Intercept | 1.935 | 2.859 | 2.041 | 2.032 | 3.600 | 0.947 | 3.635 | 3.288 | 3.643 | 3.068 | 6.978 | 3.464 | 5.924 |
| q | 0.000 | 0.000 | 0.000 | 0.000 | 0.000 | 0.000 | 0.000 | 0.000 | 0.000 | 0.000 | 0.000 | 0.000 | 0.000 |
| T2 | -0.012 | 0.006 | 0.038 | 0.122 | 0.107 | 0.101 | -0.032 | 0.005 | 0.103 | 0.132 | 0.101 | 0.073 | -0.074 |
| q | 0.881 | 0.939 | 0.738 | 0.164 | 0.473 | 0.333 | 0.840 | 0.955 | 0.535 | 0.144 | 0.496 | 0.511 | 0.615 |
| T3 | -0.026 | 0.547 | 0.031 | 0.241 | 0.045 | 0.257 | 0.118 | 0.970 | 0.092 | 0.427 | 0.430 | 0.578 | 0.444 |
| q | 0.881 | 0.000 | 0.738 | 0.005 | 0.731 | 0.010 | 0.437 | 0.000 | 0.546 | 0.000 | 0.000 | 0.000 | 0.000 |
| T4 | -0.218 | 0.556 | -0.157 | 0.207 | -0.294 | 0.030 | -0.216 | 1.035 | -0.265 | 0.434 | 0.083 | 0.339 | 0.110 |
| q | 0.025 | 0.000 | 0.161 | 0.016 | 0.014 | 0.764 | 0.141 | 0.000 | 0.043 | 0.000 | 0.501 | 0.003 | 0.535 |
| T5 | -0.770 | 0.204 | -0.712 | 0.024 | -0.997 | -0.477 | -0.966 | 0.765 | -0.991 | 0.353 | -0.625 | -0.326 | -0.694 |
| q | 0.000 | 0.060 | 0.000 | 0.770 | 0.000 | 0.000 | 0.000 | 0.000 | 0.000 | 0.000 | 0.000 | 0.010 | 0.000 |
| Radiation | 0.023 | 0.077 | 0.045 | 0.250 | 0.045 | 0.194 | 0.031 | 0.176 | 0.086 | 0.273 | 0.145 | 0.186 | 0.037 |
| q | 0.881 | 0.618 | 0.738 | 0.138 | 0.773 | 0.220 | 0.845 | 0.293 | 0.659 | 0.133 | 0.496 | 0.294 | 0.819 |
| RT*T2 | -0.175 | -0.204 | -0.157 | -0.189 | -0.077 | -0.114 | -0.088 | -0.157 | -0.078 | -0.136 | -0.159 | -0.139 | -0.132 |
| q | 0.345 | 0.206 | 0.421 | 0.188 | 0.731 | 0.554 | 0.784 | 0.371 | 0.667 | 0.375 | 0.496 | 0.511 | 0.615 |
| RT*T3 | -0.216 | -0.311 | -0.191 | -0.147 | -0.234 | -0.251 | -0.330 | -0.342 | -0.276 | -0.100 | -0.440 | -0.361 | -0.452 |
| q | 0.260 | 0.060 | 0.347 | 0.283 | 0.382 | 0.200 | 0.193 | 0.080 | 0.277 | 0.470 | 0.056 | 0.082 | 0.050 |
| RT*T4 | -0.272 | -0.224 | -0.260 | -0.212 | -0.320 | -0.255 | -0.278 | -0.196 | -0.305 | -0.133 | -0.403 | -0.297 | -0.369 |
| q | 0.140 | 0.196 | 0.161 | 0.164 | 0.184 | 0.200 | 0.270 | 0.299 | 0.252 | 0.375 | 0.072 | 0.153 | 0.114 |
| RT*T5 | 0.074 | -0.174 | 0.066 | -0.166 | 0.133 | -0.050 | 0.147 | -0.326 | 0.128 | -0.170 | -0.060 | -0.041 | 0.066 |
| q | 0.881 | 0.330 | 0.738 | 0.283 | 0.706 | 0.764 | 0.669 | 0.120 | 0.659 | 0.374 | 0.773 | 0.831 | 0.819 |

Table S2, continued:

|  | **H3FC** | **H3PL** | **H3TG** | **H4A1** | **H4A2** | **H4CH** | **H4FC** | **H4PL** | **H4TG** |
| --- | --- | --- | --- | --- | --- | --- | --- | --- | --- |
| Intercept | 3.732 | 4.475 | 3.735 | 7.459 | 6.701 | 5.029 | 4.398 | 4.761 | 6.013 |
| q | 0.000 | 0.000 | 0.000 | 0.000 | 0.000 | 0.000 | 0.000 | 0.000 | 0.000 |
| T2 | -0.026 | 0.044 | 0.118 | -0.127 | -0.170 | -0.157 | -0.180 | -0.081 | 0.031 |
| q | 0.836 | 0.864 | 0.257 | 0.326 | 0.157 | 0.219 | 0.037 | 0.473 | 0.721 |
| T3 | 0.859 | 0.197 | 0.301 | 0.271 | 0.228 | 0.035 | 0.501 | -0.014 | -0.060 |
| q | 0.000 | 0.181 | 0.001 | 0.023 | 0.094 | 0.784 | 0.000 | 0.977 | 0.715 |
| T4 | 0.796 | -0.219 | 0.241 | -0.065 | -0.110 | -0.267 | 0.432 | -0.329 | -0.290 |
| q | 0.000 | 0.181 | 0.008 | 0.645 | 0.420 | 0.016 | 0.000 | 0.002 | 0.004 |
| T5 | 0.405 | -1.085 | 0.058 | -0.665 | -0.696 | -0.769 | 0.218 | -0.865 | -0.713 |
| q | 0.000 | 0.000 | 0.579 | 0.000 | 0.000 | 0.000 | 0.034 | 0.000 | 0.000 |
| Radiation | 0.030 | -0.005 | 0.233 | -0.103 | -0.043 | -0.155 | -0.070 | -0.162 | 0.067 |
| q | 0.836 | 0.979 | 0.257 | 0.645 | 0.883 | 0.448 | 0.616 | 0.456 | 0.719 |
| RT*T2 | -0.123 | -0.057 | -0.141 | -0.065 | -0.065 | -0.009 | -0.085 | -0.005 | -0.196 |
| q | 0.540 | 0.864 | 0.433 | 0.704 | 0.883 | 0.954 | 0.616 | 0.977 | 0.399 |
| RT*T3 | -0.394 | -0.304 | -0.089 | -0.343 | -0.345 | -0.243 | -0.285 | -0.166 | -0.076 |
| q | 0.029 | 0.233 | 0.579 | 0.131 | 0.149 | 0.284 | 0.064 | 0.456 | 0.719 |
| RT*T4 | -0.265 | -0.357 | -0.143 | -0.305 | -0.317 | -0.223 | -0.160 | -0.206 | -0.209 |
| q | 0.166 | 0.181 | 0.433 | 0.167 | 0.157 | 0.296 | 0.346 | 0.429 | 0.399 |
| RT*T5 | -0.157 | 0.226 | -0.054 | 0.106 | 0.027 | 0.162 | -0.075 | 0.235 | 0.133 |
| q | 0.530 | 0.445 | 0.723 | 0.645 | 0.892 | 0.462 | 0.616 | 0.429 | 0.715 |

**Table S3: Results from linear mixed model analysis of longitudinal changes in circulating metabolites for different treatment groups.**  The models include main effect for timepoints, treatment group, and the time-group interactions as fixed effects, and a random intercept was included for each patient. The time variable was reference coded to the baseline measurement (T1). Treatment groups were sum coded in statistical analysis to detect possible groups deviating from the average response. q, Benjamini-Hochberg corrected p-values.

|  | **Lipid1** | **Leucine** | **Valine** | **Iso-**  **leucine** | **2-methyl-**  **glutarate** | **3-hydroxy-**  **butyrate** | **Alanine** | **Lipid2** | **Lysine** | **Acetate** | **Glutamate** | **Glutamine** | **Citrate** |
| --- | --- | --- | --- | --- | --- | --- | --- | --- | --- | --- | --- | --- | --- |
| Intercept | 3.532 | 4.334 | 4.409 | 3.529 | 4.024 | 4.070 | 4.908 | 1.894 | 7.102 | 3.109 | 4.733 | 4.394 | 4.527 |
| q | 0.000 | 0.000 | 0.000 | 0.000 | 0.000 | 0.000 | 0.000 | 0.000 | 0.000 | 0.000 | 0.000 | 0.000 | 0.000 |
| T2 | -0.081 | -0.021 | 0.200 | 0.022 | 0.005 | -0.128 | 0.183 | -0.050 | -0.035 | -0.096 | -0.078 | -0.168 | -0.247 |
| q | 0.359 | 0.902 | 0.028 | 0.802 | 0.930 | 0.495 | 0.059 | 0.421 | 0.898 | 0.546 | 0.442 | 0.259 | 0.037 |
| T3 | -0.055 | -0.023 | 0.151 | 0.023 | 0.029 | 0.179 | 0.073 | -0.067 | 0.245 | 0.034 | 0.306 | -0.235 | -0.024 |
| q | 0.502 | 0.902 | 0.213 | 0.802 | 0.859 | 0.150 | 0.610 | 0.329 | 0.011 | 0.760 | 0.002 | 0.091 | 0.877 |
| T4 | -0.210 | -0.090 | 0.106 | -0.029 | -0.010 | 0.014 | 0.057 | -0.139 | 0.257 | 0.168 | 0.686 | -0.646 | 0.105 |
| q | 0.009 | 0.648 | 0.435 | 0.802 | 0.930 | 0.937 | 0.666 | 0.066 | 0.009 | 0.130 | 0.000 | 0.000 | 0.544 |
| T5 | -0.510 | -0.209 | 0.027 | -0.104 | -0.130 | -0.294 | -0.095 | -0.297 | 0.172 | 0.087 | 0.901 | -1.356 | 0.265 |
| q | 0.000 | 0.043 | 0.854 | 0.477 | 0.196 | 0.006 | 0.505 | 0.000 | 0.206 | 0.573 | 0.000 | 0.000 | 0.041 |
| Group1 | 0.198 | 0.059 | -0.023 | 0.130 | 0.018 | 0.077 | 0.073 | 0.200 | 0.018 | -0.129 | 0.081 | -0.091 | 0.092 |
| q | 0.232 | 0.902 | 0.913 | 0.591 | 0.930 | 0.934 | 0.670 | 0.234 | 0.926 | 0.562 | 0.641 | 0.746 | 0.698 |
| Group2 | 0.177 | 0.168 | 0.071 | 0.165 | 0.207 | -0.051 | 0.240 | 0.149 | 0.062 | 0.098 | 0.199 | -0.167 | 0.115 |
| q | 0.415 | 0.805 | 0.854 | 0.591 | 0.327 | 0.934 | 0.338 | 0.405 | 0.898 | 0.746 | 0.424 | 0.717 | 0.698 |
| Group3 | 0.119 | 0.070 | -0.016 | 0.122 | 0.031 | -0.079 | -0.057 | 0.109 | 0.036 | -0.062 | 0.251 | -0.126 | -0.066 |
| q | 0.502 | 0.902 | 0.913 | 0.699 | 0.930 | 0.934 | 0.770 | 0.462 | 0.926 | 0.760 | 0.225 | 0.744 | 0.813 |
| Group4 | -0.208 | -0.060 | 0.085 | -0.112 | -0.012 | 0.108 | -0.027 | -0.161 | 0.041 | 0.001 | -0.258 | 0.090 | -0.053 |
| q | 0.147 | 0.902 | 0.774 | 0.591 | 0.930 | 0.934 | 0.849 | 0.234 | 0.898 | 0.988 | 0.105 | 0.744 | 0.813 |
| Gr1*T2 | -0.236 | -0.061 | 0.117 | -0.050 | 0.153 | 0.050 | 0.077 | -0.137 | 0.248 | 0.165 | 0.157 | -0.026 | 0.121 |
| q | 0.147 | 0.902 | 0.774 | 0.802 | 0.327 | 0.934 | 0.670 | 0.297 | 0.247 | 0.548 | 0.435 | 0.912 | 0.698 |
| Gr1*T3 | -0.108 | 0.015 | 0.233 | 0.034 | 0.277 | -0.051 | 0.246 | -0.127 | 0.229 | 0.248 | 0.092 | -0.344 | -0.161 |
| q | 0.502 | 0.941 | 0.333 | 0.805 | 0.144 | 0.934 | 0.245 | 0.329 | 0.272 | 0.268 | 0.641 | 0.259 | 0.606 |
| Gr1*T4 | -0.070 | 0.020 | 0.247 | 0.040 | 0.306 | -0.124 | 0.336 | -0.107 | 0.259 | 0.242 | 0.045 | -0.081 | -0.148 |
| q | 0.660 | 0.941 | 0.333 | 0.802 | 0.144 | 0.934 | 0.081 | 0.405 | 0.247 | 0.268 | 0.780 | 0.765 | 0.653 |
| Gr1*T5 | -0.026 | 0.008 | 0.105 | -0.008 | 0.267 | 0.102 | 0.106 | -0.020 | 0.107 | 0.283 | -0.206 | -0.052 | -0.349 |
| q | 0.868 | 0.953 | 0.774 | 0.951 | 0.196 | 0.934 | 0.666 | 0.901 | 0.898 | 0.268 | 0.424 | 0.883 | 0.307 |
| Gr2*T2 | -0.170 | -0.097 | -0.024 | -0.107 | -0.274 | -0.014 | -0.304 | -0.180 | -0.123 | -0.143 | -0.089 | 0.026 | -0.137 |
| q | 0.415 | 0.902 | 0.913 | 0.742 | 0.196 | 0.937 | 0.245 | 0.297 | 0.898 | 0.659 | 0.713 | 0.912 | 0.698 |
| Gr2*T3 | -0.315 | -0.049 | -0.049 | -0.122 | -0.295 | 0.015 | -0.383 | -0.192 | -0.177 | -0.115 | -0.271 | 0.313 | -0.052 |
| q | 0.147 | 0.902 | 0.874 | 0.716 | 0.196 | 0.937 | 0.139 | 0.297 | 0.723 | 0.746 | 0.340 | 0.506 | 0.877 |
| Gr2*T4 | -0.395 | -0.212 | -0.165 | -0.254 | -0.324 | -0.137 | -0.511 | -0.280 | -0.352 | -0.094 | -0.508 | 0.225 | -0.289 |
| q | 0.075 | 0.648 | 0.774 | 0.477 | 0.196 | 0.934 | 0.039 | 0.157 | 0.247 | 0.760 | 0.060 | 0.692 | 0.530 |
| Gr2*T5 | -0.408 | -0.176 | -0.123 | -0.224 | -0.316 | -0.075 | -0.685 | -0.248 | -0.116 | -0.201 | -0.085 | 0.116 | -0.023 |
| q | 0.106 | 0.805 | 0.774 | 0.549 | 0.196 | 0.934 | 0.017 | 0.234 | 0.898 | 0.607 | 0.745 | 0.765 | 0.938 |
| Gr3*T2 | -0.171 | -0.234 | -0.195 | -0.267 | -0.190 | -0.075 | -0.250 | -0.177 | -0.364 | -0.125 | -0.412 | -0.140 | -0.311 |
| q | 0.363 | 0.532 | 0.512 | 0.274 | 0.327 | 0.934 | 0.283 | 0.263 | 0.149 | 0.659 | 0.073 | 0.744 | 0.360 |
| Gr3*T3 | -0.078 | -0.159 | -0.089 | -0.189 | -0.164 | -0.077 | -0.120 | -0.052 | -0.204 | -0.066 | -0.492 | 0.271 | -0.192 |
| q | 0.660 | 0.805 | 0.774 | 0.549 | 0.379 | 0.934 | 0.666 | 0.740 | 0.523 | 0.769 | 0.043 | 0.506 | 0.606 |
| Gr3*T4 | 0.025 | -0.040 | -0.086 | -0.108 | -0.177 | 0.260 | -0.007 | 0.015 | -0.027 | 0.031 | -0.185 | 0.301 | 0.014 |
| q | 0.868 | 0.902 | 0.774 | 0.716 | 0.327 | 0.569 | 0.969 | 0.901 | 0.926 | 0.900 | 0.435 | 0.458 | 0.938 |
| Gr3*T5 | -0.269 | -0.130 | -0.098 | -0.226 | -0.242 | -0.137 | 0.125 | -0.296 | 0.002 | 0.092 | -0.181 | 0.133 | 0.218 |
| q | 0.173 | 0.879 | 0.774 | 0.477 | 0.251 | 0.934 | 0.666 | 0.103 | 0.991 | 0.760 | 0.442 | 0.746 | 0.606 |
| Gr4*T2 | 0.346 | 0.219 | 0.045 | 0.240 | 0.139 | 0.098 | 0.334 | 0.289 | 0.201 | 0.152 | 0.227 | 0.075 | 0.201 |
| q | 0.009 | 0.307 | 0.854 | 0.166 | 0.327 | 0.934 | 0.039 | 0.010 | 0.272 | 0.548 | 0.203 | 0.765 | 0.530 |
| Gr4*T3 | 0.208 | -0.029 | -0.178 | 0.058 | 0.062 | 0.020 | 0.083 | 0.157 | -0.060 | 0.006 | 0.276 | -0.293 | 0.190 |
| q | 0.147 | 0.902 | 0.435 | 0.802 | 0.794 | 0.937 | 0.666 | 0.234 | 0.898 | 0.988 | 0.148 | 0.259 | 0.530 |
| Gr4*T4 | 0.215 | -0.033 | -0.150 | 0.040 | 0.038 | -0.011 | 0.093 | 0.156 | -0.022 | 0.046 | 0.252 | -0.402 | 0.334 |
| q | 0.147 | 0.902 | 0.512 | 0.802 | 0.907 | 0.937 | 0.666 | 0.234 | 0.926 | 0.769 | 0.166 | 0.091 | 0.121 |
| Gr4*T5 | 0.449 | 0.159 | 0.092 | 0.267 | 0.270 | 0.099 | 0.428 | 0.361 | 0.050 | 0.117 | 0.275 | -0.195 | 0.059 |
| q | 0.003 | 0.648 | 0.774 | 0.166 | 0.144 | 0.934 | 0.020 | 0.004 | 0.898 | 0.659 | 0.166 | 0.592 | 0.838 |

Table S3, continued:

|  | **Creatinine** | **Ornithine** | **Proline-**  **Betaine** | **Dimethyl-**  **sulfone** | **Glucose** | **Methanol** | **Glycine** | **Creatine** | **Lactate** | **Tyrosine** | **Histidine** | **Phenyl-**  **alanine** | **Formate** |
| --- | --- | --- | --- | --- | --- | --- | --- | --- | --- | --- | --- | --- | --- |
| Intercept | 1.172 | 8.464 | 3.690 | 0.467 | 3.615 | 3.762 | 4.010 | 2.792 | 3.410 | 4.547 | 6.720 | 5.445 | 3.036 |
| q | 0.000 | 0.000 | 0.000 | 0.000 | 0.000 | 0.000 | 0.000 | 0.000 | 0.000 | 0.000 | 0.000 | 0.000 | 0.000 |
| T2 | -0.172 | -0.151 | -0.018 | 0.010 | -0.196 | 0.014 | -0.314 | -0.042 | -0.178 | 0.365 | 0.235 | 0.033 | -0.018 |
| q | 0.010 | 0.381 | 0.954 | 0.941 | 0.114 | 0.974 | 0.000 | 0.619 | 0.088 | 0.000 | 0.038 | 0.912 | 0.899 |
| T3 | -0.102 | 0.292 | 0.159 | -0.021 | 0.060 | -0.097 | -0.069 | -0.116 | -0.331 | 0.227 | 0.238 | -0.033 | 0.855 |
| q | 0.168 | 0.014 | 0.296 | 0.941 | 0.782 | 0.834 | 0.679 | 0.116 | 0.000 | 0.049 | 0.038 | 0.912 | 0.000 |
| T4 | -0.133 | 0.233 | 0.317 | -0.063 | 0.098 | -0.141 | -0.049 | -0.179 | -0.352 | 0.410 | 0.299 | 0.085 | 1.183 |
| q | 0.071 | 0.075 | 0.004 | 0.941 | 0.526 | 0.461 | 0.708 | 0.026 | 0.000 | 0.000 | 0.013 | 0.691 | 0.000 |
| T5 | -0.159 | 0.166 | 0.033 | 0.063 | -0.016 | 0.003 | -0.175 | -0.131 | -0.316 | 0.121 | -0.162 | -0.193 | 1.188 |
| q | 0.047 | 0.381 | 0.954 | 0.941 | 0.948 | 0.974 | 0.093 | 0.115 | 0.001 | 0.530 | 0.245 | 0.238 | 0.000 |
| Group1 | -0.050 | 0.088 | -0.166 | 0.212 | -0.194 | -0.029 | -0.062 | -0.119 | -0.040 | -0.073 | -0.022 | -0.122 | -0.322 |
| q | 0.663 | 0.799 | 0.606 | 0.557 | 0.526 | 0.974 | 0.746 | 0.358 | 0.979 | 0.905 | 0.957 | 0.731 | 0.130 |
| Group2 | -0.095 | 0.100 | 0.013 | -0.086 | 0.013 | -0.007 | 0.120 | -0.087 | 0.124 | -0.025 | -0.280 | -0.074 | -0.091 |
| q | 0.598 | 0.799 | 0.954 | 0.941 | 0.948 | 0.974 | 0.708 | 0.619 | 0.787 | 0.971 | 0.274 | 0.912 | 0.827 |
| Group3 | -0.102 | 0.043 | 0.082 | -0.087 | 0.064 | -0.055 | 0.067 | -0.182 | -0.147 | 0.076 | 0.036 | 0.056 | 0.167 |
| q | 0.517 | 0.874 | 0.954 | 0.941 | 0.875 | 0.974 | 0.746 | 0.210 | 0.669 | 0.905 | 0.938 | 0.912 | 0.588 |
| Group4 | 0.226 | -0.147 | -0.007 | -0.074 | 0.166 | -0.135 | -0.045 | 0.406 | 0.174 | 0.005 | 0.210 | 0.221 | 0.217 |
| q | 0.005 | 0.634 | 0.954 | 0.941 | 0.526 | 0.834 | 0.763 | 0.000 | 0.290 | 0.971 | 0.233 | 0.292 | 0.273 |
| Gr1*T2 | 0.063 | 0.041 | 0.220 | 0.115 | 0.135 | 0.175 | 0.046 | 0.256 | -0.032 | -0.083 | 0.343 | -0.013 | 0.614 |
| q | 0.663 | 0.874 | 0.558 | 0.941 | 0.782 | 0.834 | 0.782 | 0.064 | 0.979 | 0.905 | 0.121 | 0.935 | 0.010 |
| Gr1*T3 | 0.034 | -0.202 | 0.253 | -0.036 | 0.192 | 0.098 | -0.139 | 0.274 | -0.004 | 0.161 | 0.432 | 0.227 | 0.344 |
| q | 0.810 | 0.634 | 0.451 | 0.941 | 0.526 | 0.896 | 0.679 | 0.057 | 0.979 | 0.676 | 0.042 | 0.470 | 0.216 |
| Gr1*T4 | 0.074 | -0.088 | 0.333 | -0.230 | 0.207 | 0.047 | -0.077 | 0.273 | 0.122 | 0.035 | 0.474 | 0.018 | 0.265 |
| q | 0.663 | 0.799 | 0.288 | 0.557 | 0.526 | 0.974 | 0.723 | 0.058 | 0.787 | 0.971 | 0.038 | 0.935 | 0.407 |
| Gr1*T5 | 0.032 | -0.394 | 0.082 | -0.575 | 0.086 | 0.044 | -0.179 | 0.182 | -0.081 | -0.015 | 0.231 | 0.209 | 0.258 |
| q | 0.817 | 0.273 | 0.954 | 0.004 | 0.875 | 0.974 | 0.679 | 0.284 | 0.979 | 0.971 | 0.446 | 0.691 | 0.519 |
| Gr2*T2 | 0.083 | 0.095 | -0.032 | -0.014 | 0.012 | 0.122 | 0.151 | -0.042 | -0.077 | 0.349 | 0.017 | 0.140 | -0.145 |
| q | 0.663 | 0.821 | 0.954 | 0.941 | 0.948 | 0.896 | 0.701 | 0.839 | 0.979 | 0.312 | 0.978 | 0.835 | 0.751 |
| Gr2*T3 | 0.044 | 0.001 | 0.030 | -0.014 | 0.049 | 0.147 | 0.109 | 0.022 | -0.045 | 0.085 | -0.084 | 0.024 | 0.009 |
| q | 0.810 | 0.995 | 0.954 | 0.941 | 0.948 | 0.896 | 0.723 | 0.882 | 0.979 | 0.933 | 0.883 | 0.935 | 0.972 |
| Gr2*T4 | 0.031 | -0.201 | -0.226 | 0.043 | 0.084 | 0.061 | 0.198 | -0.115 | -0.294 | 0.894 | 0.465 | 0.814 | 0.212 |
| q | 0.819 | 0.753 | 0.682 | 0.941 | 0.875 | 0.974 | 0.679 | 0.616 | 0.387 | 0.001 | 0.121 | 0.002 | 0.588 |
| Gr2*T5 | 0.096 | 0.074 | -0.048 | 0.805 | -0.014 | -0.050 | -0.062 | 0.041 | -0.195 | 0.018 | -0.182 | 0.067 | -0.102 |
| q | 0.663 | 0.874 | 0.954 | 0.001 | 0.948 | 0.974 | 0.782 | 0.842 | 0.787 | 0.971 | 0.692 | 0.933 | 0.830 |
| Gr3*T2 | 0.068 | -0.302 | -0.511 | -0.030 | -0.268 | -0.340 | -0.124 | 0.061 | -0.036 | -0.353 | -0.200 | -0.298 | -0.543 |
| q | 0.663 | 0.395 | 0.053 | 0.941 | 0.526 | 0.366 | 0.702 | 0.720 | 0.979 | 0.219 | 0.500 | 0.393 | 0.052 |
| Gr3*T3 | 0.082 | -0.221 | -0.145 | -0.016 | -0.257 | -0.151 | -0.089 | 0.081 | -0.029 | -0.262 | -0.004 | -0.283 | -0.615 |
| q | 0.663 | 0.665 | 0.927 | 0.941 | 0.526 | 0.896 | 0.723 | 0.656 | 0.979 | 0.505 | 0.986 | 0.465 | 0.035 |
| Gr3*T4 | 0.124 | 0.019 | 0.093 | 0.043 | -0.211 | 0.016 | 0.020 | 0.152 | 0.197 | -0.520 | -0.163 | -0.402 | -0.615 |
| q | 0.598 | 0.960 | 0.954 | 0.941 | 0.526 | 0.974 | 0.880 | 0.366 | 0.669 | 0.047 | 0.645 | 0.224 | 0.035 |
| Gr3*T5 | 0.113 | 0.189 | 0.144 | -0.055 | -0.061 | 0.125 | 0.165 | 0.212 | 0.346 | -0.215 | 0.070 | -0.206 | -0.212 |
| q | 0.654 | 0.753 | 0.927 | 0.941 | 0.921 | 0.896 | 0.679 | 0.237 | 0.225 | 0.655 | 0.883 | 0.691 | 0.588 |
| Gr4*T2 | -0.174 | 0.113 | 0.273 | -0.027 | 0.063 | 0.101 | -0.112 | -0.214 | 0.014 | -0.028 | 0.087 | 0.118 | 0.168 |
| q | 0.163 | 0.799 | 0.288 | 0.941 | 0.875 | 0.896 | 0.679 | 0.076 | 0.979 | 0.971 | 0.734 | 0.731 | 0.588 |
| Gr4*T3 | -0.121 | 0.084 | -0.162 | 0.077 | -0.103 | 0.104 | -0.031 | -0.252 | 0.061 | -0.092 | -0.208 | -0.093 | 0.053 |
| q | 0.471 | 0.799 | 0.682 | 0.941 | 0.782 | 0.896 | 0.805 | 0.057 | 0.979 | 0.905 | 0.345 | 0.835 | 0.836 |
| Gr4*T4 | -0.205 | 0.113 | -0.029 | 0.153 | -0.113 | -0.083 | -0.227 | -0.289 | -0.010 | -0.387 | -0.414 | -0.319 | 0.067 |
| q | 0.102 | 0.799 | 0.954 | 0.920 | 0.782 | 0.896 | 0.184 | 0.027 | 0.979 | 0.049 | 0.038 | 0.224 | 0.830 |
| Gr4*T5 | -0.192 | 0.105 | 0.018 | -0.058 | 0.095 | 0.006 | -0.103 | -0.268 | -0.016 | 0.135 | 0.112 | -0.020 | 0.163 |
| q | 0.163 | 0.799 | 0.954 | 0.941 | 0.836 | 0.974 | 0.702 | 0.057 | 0.979 | 0.752 | 0.698 | 0.935 | 0.588 |

**Table S4: Results from linear mixed model analysis of longitudinal changes in circulating metabolites for local and locoregional radiation.**  The models include main effect for timepoints, treatment group, and the time-group interactions as fixed effects, and a random intercept was included for each patient. The time variable was reference coded to the baseline measurement (T1), and treatment was reference coded with local radiation as the reference group. RT, radiotherapy, q, Benjamini-Hochberg corrected p-values.

|  | **Lipid1** | **Leucine** | **Valine** | **Iso-**  **leucine** | **2-methyl-**  **glutarate** | **3-hydroxy-**  **butyrate** | **Alanine** | **Lipid2** | **Lysine** | **Acetate** | **Glutamate** | **Glutamine** | **Citrate** |
| --- | --- | --- | --- | --- | --- | --- | --- | --- | --- | --- | --- | --- | --- |
| Intercept | 3.391 | 4.199 | 4.339 | 3.375 | 3.925 | 4.076 | 4.782 | 1.754 | 7.026 | 3.122 | 4.621 | 4.431 | 4.507 |
| q | 0.000 | 0.000 | 0.000 | 0.000 | 0.000 | 0.000 | 0.000 | 0.000 | 0.000 | 0.000 | 0.000 | 0.000 | 0.000 |
| T2 | 0.067 | 0.080 | 0.197 | 0.108 | 0.071 | -0.138 | 0.262 | 0.070 | -0.010 | -0.097 | -0.059 | -0.096 | -0.204 |
| q | 0.456 | 0.378 | 0.037 | 0.269 | 0.556 | 0.226 | 0.010 | 0.366 | 0.905 | 0.437 | 0.739 | 0.468 | 0.072 |
| T3 | 0.114 | 0.077 | 0.172 | 0.124 | 0.072 | 0.202 | 0.207 | 0.043 | 0.322 | 0.016 | 0.440 | -0.184 | 0.052 |
| q | 0.217 | 0.378 | 0.053 | 0.248 | 0.556 | 0.050 | 0.055 | 0.622 | 0.000 | 0.845 | 0.000 | 0.156 | 0.858 |
| T4 | -0.037 | 0.036 | 0.180 | 0.098 | 0.092 | 0.071 | 0.189 | -0.028 | 0.365 | 0.118 | 0.832 | -0.569 | 0.214 |
| q | 0.696 | 0.614 | 0.053 | 0.269 | 0.556 | 0.661 | 0.073 | 0.660 | 0.000 | 0.355 | 0.000 | 0.000 | 0.072 |
| T5 | -0.383 | -0.116 | 0.102 | 0.029 | -0.067 | -0.302 | 0.035 | -0.182 | 0.208 | -0.017 | 1.072 | -1.444 | 0.258 |
| q | 0.000 | 0.340 | 0.382 | 0.711 | 0.592 | 0.007 | 0.800 | 0.056 | 0.075 | 0.845 | 0.000 | 0.000 | 0.072 |
| Radiation | 0.190 | 0.242 | 0.174 | 0.249 | 0.142 | 0.017 | 0.234 | 0.204 | 0.163 | -0.020 | 0.061 | 0.103 | -0.015 |
| q | 0.219 | 0.250 | 0.349 | 0.192 | 0.556 | 0.911 | 0.170 | 0.277 | 0.379 | 0.845 | 0.836 | 0.570 | 0.907 |
| RT*T2 | -0.201 | -0.126 | 0.073 | -0.057 | 0.002 | 0.046 | 0.010 | -0.136 | 0.074 | 0.072 | 0.150 | -0.184 | 0.060 |
| q | 0.217 | 0.378 | 0.634 | 0.703 | 0.987 | 0.911 | 0.946 | 0.366 | 0.743 | 0.803 | 0.583 | 0.468 | 0.858 |
| RT*T3 | -0.235 | -0.172 | -0.033 | -0.126 | 0.052 | -0.016 | -0.176 | -0.142 | -0.063 | 0.078 | 0.001 | -0.357 | -0.048 |
| q | 0.193 | 0.340 | 0.804 | 0.381 | 0.855 | 0.911 | 0.353 | 0.366 | 0.743 | 0.803 | 0.994 | 0.131 | 0.858 |
| RT*T4 | -0.265 | -0.221 | -0.145 | -0.172 | -0.122 | -0.173 | -0.201 | -0.130 | -0.179 | 0.090 | -0.039 | -0.466 | -0.132 |
| q | 0.154 | 0.256 | 0.384 | 0.269 | 0.556 | 0.477 | 0.305 | 0.366 | 0.379 | 0.803 | 0.904 | 0.040 | 0.854 |
| RT*T5 | -0.027 | -0.116 | -0.134 | -0.153 | 0.004 | 0.063 | -0.084 | -0.068 | -0.073 | 0.254 | -0.253 | 0.115 | 0.071 |
| q | 0.852 | 0.441 | 0.439 | 0.363 | 0.987 | 0.911 | 0.762 | 0.637 | 0.743 | 0.345 | 0.318 | 0.586 | 0.858 |

Table S4, continued:

|  | **Creatinine** | **Ornithine** | **Proline-**  **Betaine** | **Dimethyl-**  **sulfone** | **Glucose** | **Methanol** | **Glycine** | **Creatine** | **Lactate** | **Tyrosine** | **Histidine** | **Phenyl-**  **alanine** | **Formate** |
| --- | --- | --- | --- | --- | --- | --- | --- | --- | --- | --- | --- | --- | --- |
|  | 1.118 | 8.416 | 3.710 | 0.444 | 3.574 | 3.791 | 3.883 | 2.726 | 3.284 | 4.489 | 6.721 | 5.439 | 3.029 |
| Intercept | 0.000 | 0.000 | 0.000 | 0.000 | 0.000 | 0.000 | 0.000 | 0.000 | 0.000 | 0.000 | 0.000 | 0.000 | 0.000 |
| q | -0.116 | -0.157 | 0.064 | -0.028 | -0.170 | 0.012 | -0.250 | -0.014 | -0.037 | 0.382 | 0.193 | 0.083 | -0.020 |
| T2 | 0.068 | 0.212 | 0.947 | 0.957 | 0.104 | 0.978 | 0.001 | 0.834 | 0.657 | 0.001 | 0.181 | 0.744 | 0.960 |
| q | -0.034 | 0.374 | 0.152 | -0.004 | 0.117 | -0.108 | 0.082 | -0.134 | -0.233 | 0.316 | 0.249 | 0.023 | 0.959 |
| T3 | 0.573 | 0.001 | 0.405 | 0.957 | 0.342 | 0.617 | 0.286 | 0.100 | 0.015 | 0.007 | 0.074 | 0.813 | 0.000 |
| q | -0.064 | 0.339 | 0.324 | -0.005 | 0.209 | -0.094 | 0.063 | -0.100 | -0.249 | 0.307 | 0.166 | -0.066 | 1.209 |
| T4 | 0.321 | 0.002 | 0.005 | 0.957 | 0.069 | 0.635 | 0.399 | 0.197 | 0.013 | 0.008 | 0.269 | 0.744 | 0.000 |
| q | -0.111 | 0.197 | 0.007 | -0.026 | 0.028 | 0.002 | 0.010 | -0.149 | -0.245 | 0.180 | -0.166 | -0.246 | 1.346 |
| T5 | 0.123 | 0.198 | 0.947 | 0.957 | 0.860 | 0.979 | 0.892 | 0.100 | 0.020 | 0.244 | 0.303 | 0.136 | 0.000 |
| q | 0.288 | 0.035 | -0.048 | 0.095 | 0.136 | -0.023 | 0.306 | 0.374 | 0.388 | 0.166 | 0.156 | 0.049 | 0.083 |
| Radiation | 0.001 | 0.827 | 0.947 | 0.957 | 0.498 | 0.978 | 0.011 | 0.001 | 0.005 | 0.383 | 0.490 | 0.813 | 0.770 |
| q | -0.278 | 0.096 | -0.080 | 0.108 | -0.009 | 0.028 | -0.233 | -0.182 | -0.368 | -0.055 | 0.076 | -0.101 | 0.138 |
| RT*T2 | 0.014 | 0.816 | 0.947 | 0.957 | 0.951 | 0.978 | 0.066 | 0.188 | 0.020 | 0.817 | 0.832 | 0.744 | 0.770 |
| q | -0.301 | -0.038 | 0.030 | -0.020 | -0.109 | -0.039 | -0.434 | -0.095 | -0.283 | -0.214 | -0.102 | -0.092 | -0.105 |
| RT*T3 | 0.010 | 0.827 | 0.947 | 0.957 | 0.583 | 0.978 | 0.001 | 0.467 | 0.074 | 0.383 | 0.817 | 0.744 | 0.770 |
| q | -0.314 | -0.173 | -0.043 | -0.155 | -0.347 | -0.195 | -0.427 | -0.333 | -0.285 | 0.042 | 0.051 | 0.166 | -0.005 |
| RT*T4 | 0.009 | 0.535 | 0.947 | 0.957 | 0.069 | 0.617 | 0.001 | 0.016 | 0.074 | 0.817 | 0.864 | 0.744 | 0.982 |
| q | -0.274 | -0.057 | 0.022 | 0.011 | -0.122 | -0.040 | -0.496 | -0.149 | -0.234 | -0.072 | -0.001 | 0.130 | -0.367 |
| RT*T5 | 0.026 | 0.827 | 0.947 | 0.957 | 0.583 | 0.978 | 0.001 | 0.307 | 0.167 | 0.817 | 0.994 | 0.744 | 0.212 |
| q |  |  |  |  |  |  |  |  |  |  |  |  |  |

**Table S5: Results from linear mixed model analysis of longitudinal changes in lipoprotein subfractions for all-cause survivors and non-survivors.**  The models include main effect for timepoints, survival group, and the time-group interactions as fixed effects, and a random intercept was included for each patient. The time variable was reference coded to the baseline measurement (T1), and survival was reference coded with survivors as the reference group. q, Benjamini-Hochberg corrected p-values.

|  | **TPA1** | **TPA2** | **TPAB** | **TPCH** | **TPTG** | **VLAB** | **VLCH** | **VLFC** | **VLPL** | **VLTG** | **V1CH** | **V1FC** | **V1PL** |
| --- | --- | --- | --- | --- | --- | --- | --- | --- | --- | --- | --- | --- | --- |
| Intercept | 8.331 | 6.867 | 5.776 | 5.540 | 2.168 | 2.906 | 2.727 | 2.901 | 2.491 | 1.689 | 1.555 | 0.528 | 1.190 |
| q | 0.000 | 0.000 | 0.000 | 0.000 | 0.000 | 0.000 | 0.000 | 0.000 | 0.000 | 0.000 | 0.000 | 0.000 | 0.000 |
| T2 | -0.039 | -0.113 | -0.075 | -0.137 | 0.069 | 0.058 | -0.018 | 0.000 | 0.020 | 0.049 | 0.033 | 0.041 | 0.049 |
| q | 0.785 | 0.368 | 0.436 | 0.083 | 0.545 | 0.583 | 0.898 | 0.999 | 0.819 | 0.545 | 0.919 | 0.689 | 0.808 |
| T3 | 0.388 | 0.455 | 0.322 | 0.300 | 0.101 | 0.058 | 0.153 | 0.027 | -0.071 | 0.106 | 0.029 | 0.024 | 0.039 |
| q | 0.001 | 0.000 | 0.000 | 0.000 | 0.425 | 0.583 | 0.045 | 0.800 | 0.494 | 0.249 | 0.919 | 0.689 | 0.808 |
| T4 | 0.047 | 0.120 | 0.242 | 0.143 | 0.064 | -0.039 | 0.086 | -0.088 | -0.282 | 0.057 | 0.009 | -0.049 | -0.009 |
| q | 0.785 | 0.368 | 0.001 | 0.083 | 0.545 | 0.606 | 0.339 | 0.379 | 0.000 | 0.545 | 0.941 | 0.689 | 0.874 |
| T5 | -0.699 | -0.592 | 0.044 | -0.227 | -0.092 | -0.241 | -0.119 | -0.328 | -0.651 | -0.116 | -0.087 | -0.219 | -0.152 |
| q | 0.000 | 0.000 | 0.704 | 0.012 | 0.535 | 0.001 | 0.235 | 0.000 | 0.000 | 0.249 | 0.900 | 0.006 | 0.093 |
| Survival | -0.469 | -0.334 | -0.433 | -0.682 | 0.040 | 0.032 | -0.202 | -0.102 | 0.001 | 0.076 | -0.012 | 0.115 | 0.086 |
| q | 0.081 | 0.296 | 0.046 | 0.001 | 0.806 | 0.849 | 0.452 | 0.800 | 0.995 | 0.634 | 0.941 | 0.689 | 0.808 |
| survival*T2 | -0.099 | -0.080 | -0.144 | -0.046 | -0.086 | -0.080 | 0.006 | -0.051 | -0.109 | -0.089 | -0.073 | -0.178 | -0.112 |
| q | 0.785 | 0.760 | 0.583 | 0.890 | 0.642 | 0.637 | 0.966 | 0.800 | 0.618 | 0.634 | 0.919 | 0.689 | 0.808 |
| survival*T3 | -0.174 | -0.252 | -0.251 | -0.135 | -0.116 | -0.132 | -0.069 | -0.101 | -0.161 | -0.134 | -0.069 | -0.139 | -0.102 |
| q | 0.785 | 0.421 | 0.307 | 0.663 | 0.629 | 0.594 | 0.898 | 0.800 | 0.513 | 0.545 | 0.919 | 0.689 | 0.808 |
| survival*T4 | -0.320 | -0.367 | -0.085 | 0.016 | -0.088 | -0.105 | -0.038 | -0.063 | -0.114 | -0.088 | -0.038 | -0.065 | -0.061 |
| q | 0.494 | 0.368 | 0.704 | 0.933 | 0.642 | 0.606 | 0.898 | 0.800 | 0.618 | 0.634 | 0.941 | 0.689 | 0.808 |
| survival*T5 | 0.061 | -0.090 | -0.071 | 0.068 | 0.140 | 0.173 | 0.176 | 0.207 | 0.199 | 0.147 | 0.082 | 0.084 | 0.055 |
| q | 0.827 | 0.760 | 0.704 | 0.890 | 0.629 | 0.583 | 0.452 | 0.443 | 0.494 | 0.545 | 0.919 | 0.689 | 0.808 |

Table S5, continued:

|  | **V1TG** | **V2CH** | **V2FC** | **V2PL** | **V2TG** | **V3CH** | **V3FC** | **V3PL** | **V3TG** | **V4CH** | **V4FC** | **V4PL** | **V4TG** |
| --- | --- | --- | --- | --- | --- | --- | --- | --- | --- | --- | --- | --- | --- |
| Intercept | 1.071 | 2.633 | 2.112 | 2.892 | 3.073 | 2.218 | 1.730 | 1.928 | 3.003 | 3.032 | 1.930 | 3.086 | 2.826 |
| q | 0.000 | 0.000 | 0.000 | 0.000 | 0.000 | 0.000 | 0.000 | 0.000 | 0.000 | 0.000 | 0.000 | 0.000 | 0.000 |
| T2 | 0.073 | -0.052 | 0.013 | -0.075 | -0.016 | -0.132 | -0.049 | -0.052 | -0.085 | -0.118 | -0.022 | -0.030 | -0.032 |
| q | 0.644 | 0.717 | 0.921 | 0.496 | 0.829 | 0.092 | 0.795 | 0.521 | 0.357 | 0.172 | 0.874 | 0.749 | 0.830 |
| T3 | 0.021 | 0.322 | 0.296 | 0.509 | 0.613 | 0.327 | 0.206 | 0.302 | 0.414 | 0.057 | 0.236 | 0.105 | 0.078 |
| q | 0.897 | 0.000 | 0.000 | 0.000 | 0.000 | 0.000 | 0.003 | 0.000 | 0.000 | 0.538 | 0.000 | 0.279 | 0.489 |
| T4 | -0.006 | 0.277 | 0.332 | 0.422 | 0.629 | 0.296 | 0.195 | 0.211 | 0.399 | -0.137 | 0.217 | -0.025 | -0.049 |
| q | 0.920 | 0.000 | 0.000 | 0.000 | 0.000 | 0.000 | 0.003 | 0.003 | 0.000 | 0.126 | 0.001 | 0.749 | 0.691 |
| T5 | -0.111 | 0.009 | 0.244 | 0.038 | 0.331 | 0.066 | 0.026 | -0.117 | 0.157 | -0.519 | 0.015 | -0.333 | -0.335 |
| q | 0.437 | 0.911 | 0.002 | 0.719 | 0.000 | 0.551 | 0.862 | 0.257 | 0.121 | 0.000 | 0.874 | 0.000 | 0.000 |
| Survival | 0.147 | -0.353 | -0.091 | -0.199 | -0.258 | -0.173 | -0.034 | -0.023 | -0.164 | -0.288 | -0.141 | -0.124 | 0.007 |
| q | 0.700 | 0.112 | 0.876 | 0.496 | 0.288 | 0.551 | 0.862 | 0.925 | 0.541 | 0.203 | 0.857 | 0.749 | 0.970 |
| survival*T2 | -0.130 | 0.072 | 0.007 | 0.058 | 0.106 | 0.010 | -0.027 | -0.015 | 0.082 | -0.013 | -0.024 | -0.003 | -0.008 |
| q | 0.700 | 0.816 | 0.963 | 0.760 | 0.829 | 0.953 | 0.862 | 0.925 | 0.655 | 0.938 | 0.874 | 0.987 | 0.970 |
| survival*T3 | -0.120 | -0.059 | -0.069 | -0.117 | -0.056 | -0.080 | -0.082 | -0.139 | -0.088 | -0.162 | -0.080 | -0.123 | -0.142 |
| q | 0.700 | 0.816 | 0.876 | 0.709 | 0.829 | 0.699 | 0.862 | 0.521 | 0.655 | 0.538 | 0.857 | 0.749 | 0.691 |
| survival*T4 | -0.086 | -0.112 | -0.067 | -0.115 | -0.068 | -0.119 | -0.084 | -0.140 | -0.085 | -0.142 | -0.100 | -0.087 | -0.069 |
| q | 0.836 | 0.773 | 0.876 | 0.709 | 0.829 | 0.624 | 0.862 | 0.521 | 0.655 | 0.538 | 0.857 | 0.749 | 0.840 |
| survival*T5 | 0.036 | 0.189 | 0.165 | 0.271 | 0.314 | 0.153 | 0.182 | 0.239 | 0.297 | 0.091 | 0.124 | 0.241 | 0.314 |
| q | 0.910 | 0.615 | 0.689 | 0.454 | 0.272 | 0.551 | 0.660 | 0.340 | 0.247 | 0.682 | 0.857 | 0.365 | 0.188 |

Table S5, continued:

|  | **V5CH** | **V5FC** | **V5PL** | **V5TG** | **IDAB** | **IDCH** | **IDFC** | **IDPL** | **IDTG** | **LDAB** | **LDCH** | **LDFC** | **LDPL** |
| --- | --- | --- | --- | --- | --- | --- | --- | --- | --- | --- | --- | --- | --- |
| Intercept | 2.772 | 2.018 | 4.324 | 5.690 | 1.677 | 1.703 | 1.448 | 0.869 | 0.919 | 5.231 | 4.204 | 5.031 | 4.955 |
| q | 0.000 | 0.000 | 0.000 | 0.000 | 0.000 | 0.000 | 0.000 | 0.000 | 0.000 | 0.000 | 0.000 | 0.000 | 0.000 |
| T2 | 0.039 | 0.040 | 0.096 | 0.150 | 0.041 | -0.031 | -0.031 | -0.059 | 0.085 | -0.076 | -0.130 | -0.107 | -0.121 |
| q | 0.610 | 0.772 | 0.278 | 0.038 | 0.618 | 0.673 | 0.675 | 0.617 | 0.380 | 0.464 | 0.081 | 0.157 | 0.126 |
| T3 | -0.755 | -0.534 | -0.661 | -0.406 | 0.250 | 0.322 | 0.276 | 0.309 | 0.139 | 0.304 | 0.382 | 0.515 | 0.351 |
| q | 0.000 | 0.000 | 0.000 | 0.000 | 0.000 | 0.000 | 0.000 | 0.000 | 0.112 | 0.000 | 0.000 | 0.000 | 0.000 |
| T4 | -1.027 | -0.766 | -0.928 | -0.596 | 0.184 | 0.344 | 0.272 | 0.182 | 0.070 | 0.207 | 0.346 | 0.585 | 0.274 |
| q | 0.000 | 0.000 | 0.000 | 0.000 | 0.009 | 0.000 | 0.000 | 0.024 | 0.471 | 0.012 | 0.000 | 0.000 | 0.000 |
| T5 | -1.079 | -0.958 | -1.052 | -0.767 | -0.097 | 0.174 | 0.085 | -0.183 | -0.114 | 0.011 | 0.228 | 0.501 | 0.116 |
| q | 0.000 | 0.000 | 0.000 | 0.000 | 0.277 | 0.033 | 0.539 | 0.042 | 0.320 | 0.888 | 0.006 | 0.000 | 0.208 |
| Survival | -0.244 | 0.025 | -0.248 | -0.138 | 0.001 | -0.135 | -0.128 | -0.267 | 0.008 | -0.504 | -0.613 | -0.607 | -0.595 |
| q | 0.260 | 0.875 | 0.269 | 0.542 | 0.997 | 0.617 | 0.645 | 0.253 | 0.962 | 0.019 | 0.002 | 0.002 | 0.003 |
| survival*T2 | 0.105 | -0.144 | 0.125 | 0.025 | -0.189 | -0.104 | -0.086 | -0.059 | -0.072 | -0.142 | -0.062 | -0.053 | -0.076 |
| q | 0.610 | 0.772 | 0.522 | 0.883 | 0.333 | 0.642 | 0.675 | 0.782 | 0.800 | 0.628 | 0.719 | 0.838 | 0.739 |
| survival*T3 | 0.182 | -0.057 | 0.142 | 0.035 | -0.227 | -0.138 | -0.121 | -0.070 | -0.106 | -0.224 | -0.136 | -0.107 | -0.135 |
| q | 0.452 | 0.875 | 0.518 | 0.883 | 0.277 | 0.617 | 0.645 | 0.782 | 0.710 | 0.452 | 0.617 | 0.731 | 0.620 |
| survival*T4 | 0.262 | -0.032 | 0.251 | 0.140 | -0.242 | -0.148 | -0.137 | -0.051 | -0.043 | -0.043 | 0.087 | -0.006 | 0.050 |
| q | 0.302 | 0.875 | 0.278 | 0.542 | 0.277 | 0.617 | 0.645 | 0.782 | 0.885 | 0.888 | 0.719 | 0.973 | 0.781 |
| survival*T5 | 0.354 | 0.133 | 0.400 | 0.324 | -0.052 | -0.025 | 0.023 | 0.146 | 0.178 | -0.106 | -0.071 | -0.098 | -0.079 |
| q | 0.188 | 0.772 | 0.109 | 0.120 | 0.843 | 0.885 | 0.888 | 0.617 | 0.471 | 0.738 | 0.719 | 0.731 | 0.739 |

Table S5, continued:

|  | **LDTG** | **L1AB** | **L1CH** | **L1FC** | **L1PL** | **L1TG** | **L2AB** | **L2CH** | **L2FC** | **L2PL** | **L2TG** | **L3AB** | **L3CH** |
| --- | --- | --- | --- | --- | --- | --- | --- | --- | --- | --- | --- | --- | --- |
| Intercept | 4.142 | 3.819 | 3.130 | 3.224 | 3.645 | 3.415 | 4.441 | 3.946 | 4.697 | 4.227 | 3.590 | 3.043 | 2.574 |
| q | 0.000 | 0.000 | 0.000 | 0.000 | 0.000 | 0.000 | 0.000 | 0.000 | 0.000 | 0.000 | 0.000 | 0.000 | 0.000 |
| T2 | 0.073 | -0.051 | -0.098 | -0.103 | -0.078 | 0.108 | 0.048 | 0.000 | -0.055 | -0.002 | 0.206 | -0.002 | -0.038 |
| q | 0.341 | 0.491 | 0.188 | 0.164 | 0.380 | 0.130 | 0.547 | 0.998 | 0.539 | 0.973 | 0.007 | 0.975 | 0.754 |
| T3 | 0.482 | 0.044 | 0.008 | 0.163 | 0.010 | 0.195 | 0.351 | 0.274 | 0.391 | 0.290 | 0.429 | 0.697 | 0.627 |
| q | 0.000 | 0.525 | 0.991 | 0.025 | 0.865 | 0.008 | 0.000 | 0.000 | 0.000 | 0.000 | 0.000 | 0.000 | 0.000 |
| T4 | 0.541 | -0.063 | -0.100 | 0.108 | -0.113 | 0.217 | 0.546 | 0.439 | 0.551 | 0.450 | 0.445 | 0.958 | 0.874 |
| q | 0.000 | 0.425 | 0.188 | 0.164 | 0.155 | 0.003 | 0.000 | 0.000 | 0.000 | 0.000 | 0.000 | 0.000 | 0.000 |
| T5 | 0.426 | -0.201 | -0.219 | -0.036 | -0.260 | 0.180 | 0.879 | 0.737 | 0.722 | 0.731 | 0.377 | 1.249 | 1.170 |
| q | 0.000 | 0.019 | 0.007 | 0.736 | 0.001 | 0.031 | 0.000 | 0.000 | 0.000 | 0.000 | 0.000 | 0.000 | 0.000 |
| Survival | 0.065 | -0.344 | -0.476 | -0.526 | -0.425 | 0.080 | -0.222 | -0.306 | -0.405 | -0.316 | 0.141 | -0.331 | -0.389 |
| q | 0.735 | 0.173 | 0.022 | 0.014 | 0.053 | 0.663 | 0.399 | 0.190 | 0.071 | 0.161 | 0.461 | 0.152 | 0.069 |
| survival*T2 | -0.240 | -0.190 | -0.107 | -0.068 | -0.143 | -0.223 | -0.144 | -0.100 | -0.086 | -0.088 | -0.360 | -0.063 | -0.012 |
| q | 0.262 | 0.375 | 0.609 | 0.736 | 0.452 | 0.217 | 0.540 | 0.701 | 0.640 | 0.758 | 0.067 | 0.922 | 0.948 |
| survival*T3 | -0.350 | -0.241 | -0.158 | -0.121 | -0.190 | -0.279 | -0.144 | -0.085 | -0.163 | -0.068 | -0.447 | 0.011 | 0.065 |
| q | 0.106 | 0.319 | 0.515 | 0.628 | 0.380 | 0.130 | 0.540 | 0.701 | 0.539 | 0.770 | 0.026 | 0.975 | 0.805 |
| survival*T4 | -0.413 | -0.103 | -0.002 | -0.037 | -0.056 | -0.298 | 0.111 | 0.193 | 0.093 | 0.186 | -0.377 | 0.112 | 0.191 |
| q | 0.076 | 0.536 | 0.991 | 0.825 | 0.818 | 0.130 | 0.567 | 0.431 | 0.640 | 0.438 | 0.067 | 0.823 | 0.551 |
| survival*T5 | -0.251 | -0.214 | -0.143 | -0.134 | -0.176 | -0.169 | -0.309 | -0.244 | -0.254 | -0.225 | -0.355 | -0.179 | -0.103 |
| q | 0.262 | 0.375 | 0.551 | 0.628 | 0.420 | 0.372 | 0.225 | 0.323 | 0.329 | 0.369 | 0.079 | 0.624 | 0.754 |

Table S5, continued:

|  | **L3FC** | **L3PL** | **L3TG** | **L4AB** | **L4CH** | **L4FC** | **L4PL** | **L4TG** | **L5AB** | **L5CH** | **L5FC** | **L5PL** | **L5TG** |
| --- | --- | --- | --- | --- | --- | --- | --- | --- | --- | --- | --- | --- | --- |
| Intercept | 3.623 | 2.972 | 5.576 | 0.938 | 0.839 | 2.145 | 1.107 | 1.522 | 1.987 | 1.646 | 2.651 | 1.903 | 1.711 |
| q | 0.000 | 0.000 | 0.000 | 0.000 | 0.000 | 0.000 | 0.000 | 0.000 | 0.000 | 0.000 | 0.000 | 0.000 | 0.000 |
| T2 | -0.100 | -0.031 | 0.185 | 0.001 | -0.081 | -0.103 | -0.078 | 0.101 | -0.066 | -0.080 | -0.170 | -0.041 | 0.050 |
| q | 0.263 | 0.812 | 0.023 | 0.995 | 0.547 | 0.302 | 0.612 | 0.425 | 0.738 | 0.849 | 0.128 | 0.996 | 0.591 |
| T3 | 0.607 | 0.622 | -0.156 | 0.908 | 0.806 | 0.871 | 0.811 | 0.683 | 0.289 | 0.278 | 0.381 | 0.272 | 0.302 |
| q | 0.000 | 0.000 | 0.065 | 0.000 | 0.000 | 0.000 | 0.000 | 0.000 | 0.020 | 0.023 | 0.000 | 0.026 | 0.005 |
| T4 | 0.856 | 0.853 | -0.346 | 1.022 | 0.928 | 1.032 | 0.907 | 0.752 | 0.001 | -0.003 | 0.256 | -0.006 | 0.125 |
| q | 0.000 | 0.000 | 0.000 | 0.000 | 0.000 | 0.000 | 0.000 | 0.000 | 0.989 | 0.978 | 0.021 | 0.996 | 0.350 |
| T5 | 1.078 | 1.129 | -0.519 | 1.011 | 0.969 | 1.048 | 0.917 | 0.609 | -0.504 | -0.485 | -0.139 | -0.462 | -0.339 |
| q | 0.000 | 0.000 | 0.000 | 0.000 | 0.000 | 0.000 | 0.000 | 0.000 | 0.000 | 0.000 | 0.310 | 0.000 | 0.005 |
| Survival | -0.493 | -0.387 | -0.054 | -0.366 | -0.346 | -0.462 | -0.424 | -0.053 | -0.423 | -0.488 | -0.502 | -0.463 | -0.118 |
| q | 0.019 | 0.062 | 0.775 | 0.152 | 0.200 | 0.023 | 0.062 | 0.791 | 0.127 | 0.056 | 0.032 | 0.076 | 0.591 |
| survival*T2 | 0.046 | -0.008 | -0.339 | -0.013 | 0.055 | 0.054 | 0.078 | -0.111 | -0.045 | -0.016 | 0.031 | 0.011 | -0.151 |
| q | 0.829 | 0.962 | 0.116 | 0.995 | 0.822 | 0.877 | 0.856 | 0.703 | 0.964 | 0.978 | 0.897 | 0.996 | 0.591 |
| survival*T3 | 0.040 | 0.058 | -0.269 | -0.118 | -0.146 | -0.068 | -0.041 | -0.226 | -0.210 | -0.157 | -0.156 | -0.153 | -0.305 |
| q | 0.829 | 0.829 | 0.191 | 0.778 | 0.684 | 0.877 | 0.856 | 0.477 | 0.738 | 0.917 | 0.657 | 0.996 | 0.350 |
| survival*T4 | 0.124 | 0.167 | -0.245 | -0.154 | -0.109 | -0.005 | -0.064 | -0.405 | -0.185 | -0.069 | -0.161 | -0.097 | -0.450 |
| q | 0.756 | 0.629 | 0.241 | 0.775 | 0.746 | 0.982 | 0.856 | 0.204 | 0.738 | 0.978 | 0.657 | 0.996 | 0.199 |
| survival*T5 | -0.063 | -0.120 | -0.303 | -0.157 | -0.225 | -0.075 | -0.109 | -0.164 | -0.049 | 0.022 | -0.050 | -0.001 | -0.156 |
| q | 0.829 | 0.755 | 0.184 | 0.775 | 0.547 | 0.877 | 0.856 | 0.637 | 0.964 | 0.978 | 0.897 | 0.996 | 0.591 |

Table S5, continued:

|  | **L6AB** | **L6CH** | **L6FC** | **L6PL** | **L6TG** | **HDA1** | **HDA2** | **HDCH** | **HDFC** | **HDPL** | **HDTG** | **H1A1** | **H1A2** |
| --- | --- | --- | --- | --- | --- | --- | --- | --- | --- | --- | --- | --- | --- |
| Intercept | 3.197 | 3.110 | 3.896 | 3.144 | 4.121 | 7.855 | 7.134 | 4.745 | 7.383 | 4.883 | 4.037 | 1.879 | 0.936 |
| q | 0.000 | 0.000 | 0.000 | 0.000 | 0.000 | 0.000 | 0.000 | 0.000 | 0.000 | 0.000 | 0.000 | 0.000 | 0.000 |
| T2 | -0.128 | -0.167 | -0.268 | -0.158 | -0.082 | -0.041 | -0.080 | -0.109 | -0.061 | -0.022 | 0.115 | 0.086 | 0.118 |
| q | 0.172 | 0.087 | 0.003 | 0.118 | 0.544 | 0.766 | 0.602 | 0.473 | 0.670 | 0.898 | 0.133 | 0.377 | 0.173 |
| T3 | -0.201 | -0.212 | 0.067 | -0.241 | 0.021 | 0.287 | 0.362 | -0.084 | 0.454 | 0.003 | 0.369 | 0.088 | 0.149 |
| q | 0.022 | 0.031 | 0.828 | 0.013 | 0.784 | 0.021 | 0.001 | 0.617 | 0.000 | 0.973 | 0.000 | 0.377 | 0.105 |
| T4 | -0.394 | -0.475 | -0.049 | -0.511 | -0.151 | -0.104 | -0.003 | -0.469 | 0.479 | -0.381 | 0.299 | -0.073 | -0.015 |
| q | 0.000 | 0.000 | 0.839 | 0.000 | 0.163 | 0.523 | 0.974 | 0.000 | 0.000 | 0.000 | 0.000 | 0.447 | 0.839 |
| T5 | -0.765 | -0.935 | -0.434 | -0.992 | -0.599 | -0.864 | -0.703 | -1.118 | 0.118 | -1.039 | 0.101 | -0.541 | -0.463 |
| q | 0.000 | 0.000 | 0.000 | 0.000 | 0.000 | 0.000 | 0.000 | 0.000 | 0.322 | 0.000 | 0.195 | 0.000 | 0.000 |
| Survival | -0.134 | -0.226 | -0.398 | -0.205 | 0.077 | -0.439 | -0.274 | -0.406 | -0.716 | -0.272 | 0.291 | 0.019 | 0.210 |
| q | 0.624 | 0.374 | 0.087 | 0.457 | 0.769 | 0.128 | 0.398 | 0.140 | 0.000 | 0.500 | 0.133 | 0.911 | 0.294 |
| survival*T2 | -0.047 | -0.038 | 0.089 | -0.038 | -0.173 | -0.122 | -0.123 | -0.071 | 0.082 | -0.109 | -0.279 | -0.311 | -0.368 |
| q | 0.810 | 0.862 | 0.839 | 0.861 | 0.544 | 0.766 | 0.709 | 0.775 | 0.749 | 0.898 | 0.133 | 0.234 | 0.105 |
| survival*T3 | -0.108 | -0.108 | -0.026 | -0.099 | -0.194 | -0.191 | -0.296 | -0.103 | 0.062 | -0.077 | -0.352 | -0.166 | -0.222 |
| q | 0.727 | 0.773 | 0.903 | 0.814 | 0.544 | 0.678 | 0.432 | 0.775 | 0.749 | 0.898 | 0.100 | 0.464 | 0.303 |
| survival*T4 | -0.071 | -0.057 | -0.053 | -0.059 | -0.198 | -0.310 | -0.379 | -0.073 | -0.066 | -0.184 | -0.527 | -0.338 | -0.389 |
| q | 0.810 | 0.862 | 0.903 | 0.861 | 0.544 | 0.523 | 0.398 | 0.775 | 0.749 | 0.898 | 0.014 | 0.234 | 0.105 |
| survival*T5 | 0.162 | 0.144 | 0.101 | 0.179 | 0.083 | 0.056 | -0.138 | 0.152 | 0.143 | 0.126 | -0.296 | -0.072 | -0.137 |
| q | 0.624 | 0.763 | 0.839 | 0.634 | 0.769 | 0.845 | 0.709 | 0.775 | 0.692 | 0.898 | 0.133 | 0.796 | 0.551 |

Table S5, continued:

|  | **H1CH** | **H1FC** | **H1PL** | **H1TG** | **H2A1** | **H2A2** | **H2CH** | **H2FC** | **H2PL** | **H2TG** | **H3A1** | **H3A2** | **H3CH** |
| --- | --- | --- | --- | --- | --- | --- | --- | --- | --- | --- | --- | --- | --- |
| Intercept | 1.958 | 2.928 | 2.051 | 2.049 | 3.600 | 0.974 | 3.676 | 3.395 | 3.659 | 3.105 | 7.098 | 3.540 | 6.029 |
| q | 0.000 | 0.000 | 0.000 | 0.000 | 0.000 | 0.000 | 0.000 | 0.000 | 0.000 | 0.000 | 0.000 | 0.000 | 0.000 |
| T2 | -0.036 | -0.046 | 0.027 | 0.119 | 0.121 | 0.110 | -0.044 | -0.046 | 0.114 | 0.130 | 0.066 | 0.052 | -0.130 |
| q | 0.681 | 0.740 | 0.991 | 0.074 | 0.346 | 0.200 | 0.823 | 0.821 | 0.548 | 0.094 | 0.700 | 0.615 | 0.358 |
| T3 | -0.075 | 0.456 | -0.007 | 0.254 | 0.004 | 0.219 | 0.031 | 0.866 | 0.039 | 0.452 | 0.323 | 0.527 | 0.316 |
| q | 0.599 | 0.000 | 0.995 | 0.000 | 0.967 | 0.016 | 0.823 | 0.000 | 0.718 | 0.000 | 0.004 | 0.000 | 0.005 |
| T4 | -0.288 | 0.509 | -0.209 | 0.210 | -0.350 | -0.008 | -0.307 | 0.996 | -0.338 | 0.445 | -0.020 | 0.294 | -0.010 |
| q | 0.000 | 0.000 | 0.011 | 0.004 | 0.000 | 0.912 | 0.004 | 0.000 | 0.001 | 0.000 | 0.840 | 0.003 | 0.921 |
| T5 | -0.739 | 0.153 | -0.678 | 0.011 | -0.933 | -0.484 | -0.951 | 0.665 | -0.961 | 0.336 | -0.659 | -0.304 | -0.700 |
| q | 0.000 | 0.122 | 0.000 | 0.882 | 0.000 | 0.000 | 0.000 | 0.000 | 0.000 | 0.000 | 0.000 | 0.006 | 0.000 |
| Survival | -0.130 | -0.328 | 0.001 | 0.408 | 0.082 | 0.262 | -0.232 | -0.337 | 0.076 | 0.322 | -0.252 | -0.063 | -0.562 |
| q | 0.681 | 0.122 | 0.995 | 0.032 | 0.788 | 0.200 | 0.650 | 0.124 | 0.718 | 0.119 | 0.609 | 0.743 | 0.021 |
| survival*T2 | -0.222 | -0.088 | -0.275 | -0.372 | -0.266 | -0.302 | -0.103 | 0.023 | -0.234 | -0.240 | -0.097 | -0.136 | 0.096 |
| q | 0.578 | 0.794 | 0.330 | 0.037 | 0.422 | 0.200 | 0.823 | 0.993 | 0.614 | 0.189 | 0.840 | 0.615 | 0.921 |
| survival*T3 | -0.104 | -0.020 | -0.135 | -0.373 | -0.213 | -0.279 | -0.082 | 0.002 | -0.207 | -0.332 | -0.180 | -0.375 | -0.050 |
| q | 0.681 | 0.916 | 0.773 | 0.037 | 0.519 | 0.200 | 0.823 | 0.993 | 0.614 | 0.094 | 0.700 | 0.175 | 0.921 |
| survival*T4 | -0.129 | -0.195 | -0.238 | -0.504 | -0.366 | -0.330 | -0.013 | -0.197 | -0.202 | -0.368 | -0.249 | -0.393 | -0.052 |
| q | 0.681 | 0.539 | 0.440 | 0.011 | 0.344 | 0.200 | 0.958 | 0.647 | 0.614 | 0.094 | 0.697 | 0.175 | 0.921 |
| survival*T5 | 0.004 | -0.021 | -0.044 | -0.276 | -0.093 | -0.076 | 0.250 | -0.042 | 0.096 | -0.252 | 0.081 | -0.190 | 0.225 |
| q | 0.984 | 0.916 | 0.995 | 0.132 | 0.788 | 0.798 | 0.665 | 0.993 | 0.718 | 0.189 | 0.840 | 0.615 | 0.673 |

Table S5, continued:

|  | **H3FC** | **H3PL** | **H3TG** | **H4A1** | **H4A2** | **H4CH** | **H4FC** | **H4PL** | **H4TG** |
| --- | --- | --- | --- | --- | --- | --- | --- | --- | --- |
| Intercept | 3.809 | 4.511 | 3.776 | 7.509 | 6.764 | 5.052 | 4.447 | 4.766 | 6.033 |
| q | 0.000 | 0.000 | 0.000 | 0.000 | 0.000 | 0.000 | 0.000 | 0.000 | 0.000 |
| T2 | -0.072 | 0.035 | 0.105 | -0.164 | -0.204 | -0.182 | -0.234 | -0.101 | -0.015 |
| q | 0.522 | 0.814 | 0.178 | 0.126 | 0.060 | 0.055 | 0.001 | 0.442 | 0.849 |
| T3 | 0.760 | 0.129 | 0.340 | 0.163 | 0.124 | -0.054 | 0.409 | -0.081 | -0.040 |
| q | 0.000 | 0.551 | 0.000 | 0.126 | 0.305 | 0.655 | 0.000 | 0.568 | 0.814 |
| T4 | 0.742 | -0.310 | 0.255 | -0.159 | -0.201 | -0.350 | 0.389 | -0.399 | -0.298 |
| q | 0.000 | 0.010 | 0.001 | 0.126 | 0.060 | 0.000 | 0.000 | 0.000 | 0.001 |
| T5 | 0.380 | -1.020 | 0.094 | -0.638 | -0.682 | -0.733 | 0.195 | -0.809 | -0.623 |
| q | 0.000 | 0.000 | 0.292 | 0.000 | 0.000 | 0.000 | 0.022 | 0.000 | 0.000 |
| Survival | -0.482 | -0.216 | 0.197 | -0.605 | -0.526 | -0.530 | -0.527 | -0.420 | -0.059 |
| q | 0.018 | 0.672 | 0.317 | 0.012 | 0.047 | 0.020 | 0.004 | 0.086 | 0.842 |
| survival*T2 | 0.063 | -0.058 | -0.179 | 0.111 | 0.093 | 0.137 | 0.185 | 0.113 | -0.091 |
| q | 0.763 | 0.828 | 0.317 | 0.779 | 0.870 | 0.655 | 0.428 | 0.736 | 0.814 |
| survival*T3 | -0.134 | -0.158 | -0.389 | 0.009 | -0.034 | 0.083 | 0.026 | 0.095 | -0.246 |
| q | 0.630 | 0.796 | 0.062 | 0.968 | 0.947 | 0.781 | 0.938 | 0.736 | 0.455 |
| survival*T4 | -0.265 | -0.225 | -0.423 | -0.093 | -0.131 | 0.034 | -0.099 | -0.013 | -0.438 |
| q | 0.384 | 0.711 | 0.062 | 0.779 | 0.863 | 0.881 | 0.751 | 0.954 | 0.104 |
| survival*T5 | -0.127 | 0.107 | -0.309 | 0.111 | 0.017 | 0.160 | 0.015 | 0.191 | -0.217 |
| q | 0.630 | 0.814 | 0.178 | 0.779 | 0.947 | 0.655 | 0.938 | 0.584 | 0.528 |

**Table S6: Results from linear mixed model analysis of longitudinal changes in circulating metabolites for all-cause survivors and non-survivors.**  The models include main effect for timepoints, survival group, and the time-group interactions as fixed effects, and a random intercept was included for each patient. The time variable was reference coded to the baseline measurement (T1), and survival was reference coded with survivors as the reference group. q, Benjamini-Hochberg corrected p-values.

|  | **Lipid1** | **Leucine** | **Valine** | **Iso-**  **leucine** | **2-methyl-**  **glutarate** | **3-hydroxy-**  **butyrate** | **Alanine** | **Lipid2** | **Lysine** | **Acetate** | **Glutamate** | **Glutamine** | **Citrate** |
| --- | --- | --- | --- | --- | --- | --- | --- | --- | --- | --- | --- | --- | --- |
| Intercept | 3.448 | 4.258 | 4.385 | 3.438 | 3.928 | 4.035 | 4.827 | 1.794 | 7.070 | 3.022 | 4.593 | 4.528 | 4.488 |
| q | 0.000 | 0.000 | 0.000 | 0.000 | 0.000 | 0.000 | 0.000 | 0.000 | 0.000 | 0.000 | 0.000 | 0.000 | 0.000 |
| T2 | 0.032 | 0.072 | 0.249 | 0.121 | 0.133 | -0.071 | 0.275 | 0.053 | 0.027 | -0.007 | -0.013 | -0.148 | -0.139 |
| q | 0.784 | 0.375 | 0.001 | 0.231 | 0.049 | 0.362 | 0.001 | 0.474 | 0.769 | 0.916 | 0.974 | 0.258 | 0.228 |
| T3 | 0.080 | 0.050 | 0.179 | 0.104 | 0.140 | 0.257 | 0.212 | 0.031 | 0.324 | 0.103 | 0.474 | -0.282 | 0.029 |
| q | 0.363 | 0.483 | 0.026 | 0.317 | 0.049 | 0.003 | 0.020 | 0.642 | 0.000 | 0.126 | 0.000 | 0.012 | 0.819 |
| T4 | -0.084 | -0.010 | 0.150 | 0.067 | 0.107 | 0.067 | 0.154 | -0.040 | 0.329 | 0.208 | 0.820 | -0.700 | 0.191 |
| q | 0.363 | 0.871 | 0.059 | 0.370 | 0.117 | 0.362 | 0.088 | 0.593 | 0.000 | 0.003 | 0.000 | 0.000 | 0.081 |
| T5 | -0.395 | -0.130 | 0.065 | -0.009 | -0.036 | -0.239 | 0.036 | -0.206 | 0.224 | 0.179 | 1.021 | -1.416 | 0.332 |
| q | 0.000 | 0.357 | 0.482 | 0.893 | 0.616 | 0.014 | 0.758 | 0.006 | 0.022 | 0.017 | 0.000 | 0.000 | 0.003 |
| Survival | 0.041 | 0.180 | 0.174 | 0.194 | 0.399 | 0.394 | 0.271 | 0.192 | 0.184 | 0.637 | 0.245 | -0.097 | 0.269 |
| q | 0.901 | 0.375 | 0.482 | 0.370 | 0.038 | 0.039 | 0.211 | 0.350 | 0.472 | 0.000 | 0.375 | 0.806 | 0.228 |
| survival*T2 | -0.197 | -0.203 | -0.167 | -0.204 | -0.423 | -0.394 | -0.025 | -0.185 | -0.057 | -0.460 | 0.040 | -0.062 | -0.316 |
| q | 0.363 | 0.375 | 0.482 | 0.370 | 0.038 | 0.060 | 0.899 | 0.350 | 0.769 | 0.009 | 0.974 | 0.898 | 0.245 |
| survival*T3 | -0.258 | -0.207 | -0.155 | -0.168 | -0.388 | -0.422 | -0.399 | -0.229 | -0.157 | -0.458 | -0.204 | -0.140 | 0.033 |
| q | 0.345 | 0.375 | 0.482 | 0.370 | 0.038 | 0.055 | 0.088 | 0.350 | 0.523 | 0.009 | 0.490 | 0.806 | 0.878 |
| survival*T4 | -0.293 | -0.194 | -0.142 | -0.194 | -0.419 | -0.410 | -0.223 | -0.224 | -0.170 | -0.437 | 0.004 | -0.269 | -0.159 |
| q | 0.345 | 0.375 | 0.482 | 0.370 | 0.038 | 0.060 | 0.409 | 0.350 | 0.523 | 0.017 | 0.987 | 0.529 | 0.610 |
| survival*T5 | 0.012 | -0.159 | -0.085 | -0.105 | -0.237 | -0.300 | -0.183 | -0.020 | -0.243 | -0.677 | -0.245 | 0.014 | -0.314 |
| q | 0.947 | 0.452 | 0.642 | 0.597 | 0.196 | 0.174 | 0.479 | 0.896 | 0.472 | 0.000 | 0.473 | 0.960 | 0.249 |

Table S6, continued:

|  | **Creatinine** | **Ornithine** | **Proline-**  **Betaine** | **Dimethyl-**  **sulfone** | **Glucose** | **Methanol** | **Glycine** | **Creatine** | **Lactate** | **Tyrosine** | **Histidine** | **Phenyl-**  **alanine** | **Formate** |
| --- | --- | --- | --- | --- | --- | --- | --- | --- | --- | --- | --- | --- | --- |
| Intercept | 1.208 | 8.370 | 3.691 | 0.420 | 3.554 | 3.789 | 3.962 | 2.839 | 3.365 | 4.547 | 6.801 | 5.426 | 3.037 |
| q | 0.000 | 0.000 | 0.000 | 0.000 | 0.000 | 0.000 | 0.000 | 0.000 | 0.000 | 0.000 | 0.000 | 0.000 | 0.000 |
| T2 | -0.208 | -0.119 | 0.042 | -0.019 | -0.140 | 0.040 | -0.342 | -0.065 | -0.151 | 0.354 | 0.244 | 0.047 | 0.005 |
| q | 0.000 | 0.280 | 0.695 | 0.945 | 0.101 | 0.730 | 0.000 | 0.535 | 0.085 | 0.000 | 0.017 | 0.876 | 0.964 |
| T3 | -0.125 | 0.372 | 0.214 | -0.005 | 0.124 | -0.161 | -0.056 | -0.184 | -0.289 | 0.266 | 0.268 | 0.029 | 0.937 |
| q | 0.041 | 0.000 | 0.050 | 0.945 | 0.151 | 0.091 | 0.544 | 0.008 | 0.001 | 0.010 | 0.017 | 0.876 | 0.000 |
| T4 | -0.171 | 0.286 | 0.348 | -0.007 | 0.149 | -0.174 | -0.081 | -0.227 | -0.343 | 0.344 | 0.247 | 0.024 | 1.208 |
| q | 0.003 | 0.004 | 0.000 | 0.945 | 0.101 | 0.081 | 0.460 | 0.001 | 0.000 | 0.001 | 0.017 | 0.876 | 0.000 |
| T5 | -0.202 | 0.226 | 0.074 | 0.083 | 0.067 | 0.019 | -0.128 | -0.196 | -0.255 | 0.135 | -0.143 | -0.200 | 1.244 |
| q | 0.002 | 0.057 | 0.644 | 0.535 | 0.436 | 0.881 | 0.218 | 0.010 | 0.007 | 0.385 | 0.241 | 0.219 | 0.000 |
| Survival | 0.019 | 0.320 | 0.094 | 0.351 | 0.441 | 0.024 | 0.088 | 0.055 | 0.298 | -0.051 | 0.117 | 0.287 | 0.100 |
| q | 0.962 | 0.144 | 0.695 | 0.155 | 0.049 | 0.881 | 0.690 | 0.849 | 0.088 | 0.796 | 0.553 | 0.424 | 0.921 |
| survival*T2 | 0.013 | -0.015 | -0.024 | 0.188 | -0.219 | -0.112 | 0.150 | -0.041 | -0.029 | 0.101 | -0.139 | 0.035 | 0.141 |
| q | 0.962 | 0.973 | 0.915 | 0.535 | 0.292 | 0.730 | 0.544 | 0.880 | 0.882 | 0.741 | 0.553 | 0.876 | 0.921 |
| survival*T3 | -0.036 | -0.055 | -0.336 | -0.067 | -0.301 | 0.252 | 0.011 | 0.109 | -0.234 | -0.108 | -0.325 | -0.245 | -0.081 |
| q | 0.962 | 0.973 | 0.270 | 0.945 | 0.156 | 0.444 | 0.946 | 0.684 | 0.288 | 0.741 | 0.241 | 0.555 | 0.956 |
| survival*T4 | 0.035 | -0.008 | -0.300 | -0.367 | -0.394 | 0.108 | 0.055 | 0.132 | 0.034 | -0.173 | -0.484 | -0.266 | -0.013 |
| q | 0.962 | 0.973 | 0.343 | 0.168 | 0.101 | 0.730 | 0.824 | 0.684 | 0.882 | 0.741 | 0.103 | 0.555 | 0.964 |
| survival*T5 | 0.007 | -0.296 | -0.367 | -0.585 | -0.492 | -0.157 | -0.148 | 0.011 | -0.374 | 0.121 | -0.184 | -0.047 | -0.184 |
| q | 0.962 | 0.313 | 0.270 | 0.019 | 0.061 | 0.730 | 0.544 | 0.945 | 0.101 | 0.741 | 0.553 | 0.876 | 0.921 |
